# Targeting LAPTM5 enhances AML sensitivity to cytarabine through autophagy inhibition

**DOI:** 10.1038/s41419-026-08654-9

**Published:** 2026-03-30

**Authors:** Yuqing Zeng, Chao He, Hongbo Chen, Fang Cheng

**Affiliations:** 1 School of Pharmaceutical Sciences (Shenzhen), Sun Yat-sen University, Shenzhen, PR China; 2 Department of Endocrinology, Southwest Hospital, Army Medical University (The Third Military Medical University), Chongqing, PR China

**Keywords:** Cancer therapeutic resistance, Acute myeloid leukaemia

## Abstract

Upregulation of autophagy in acute myeloid leukemia (AML) cells contributes to the development of resistance to cytarabine (AraC). LAPTM5 is mainly expressed in hematopoietic and immune cells, and has been associated with the progression of multiple cancers; however, its role in AML drug resistance remains uncharacterized. Here, we reanalyzed publicly available single-cell RNA sequencing (scRNA-seq) data from AML patients and found distinct gene expression profiles between AraC-resistant AML cells and untreated controls. Differentially expressed genes were significantly enriched in lysosome-related pathways, with LAPTM5 being highly expressed in drug-resistant cells, suggesting that it may be a key mediator of AraC resistance in AML. Mechanistically, AraC-resistant cells exhibited enhanced autophagic flux supported by LAPTM5-mediated upregulation of LAMP1 and LAMP2. Conversely, LAPTM5 knockdown impaired autophagolysosome formation by disrupting lysosomal biogenesis, thereby sensitizing resistant cells to AraC. These findings indicate that targeting LAPTM5 could enhance AraC sensitivity in AML by modulating autophagy. In vivo experiments further confirmed that the depletion of LAPTM5 inhibited tumor growth and synergistically suppressed AML progression with AraC. Collectively, our study identifies LAPTM5 as a critical regulator of AraC resistance via autophagy modulation, highlighting its potential as a therapeutic target for AML.

**Figure:**
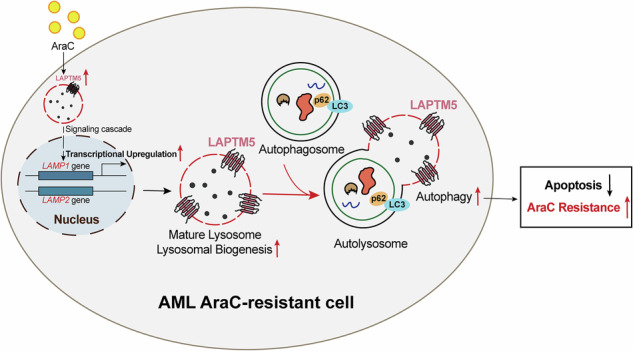
In AML, AraC treatment induces LAPTM5 upregulation, which promotes *LAMP1/2* transcription and lysosomal biogenesis. This facilitates autophagolysosome formation and enhances autophagic flux to reduce AraC-induced apoptosis, resulting in drug resistance. Targeting LAPTM5 represents a promising strategy to overcome this autophagy-mediated resistance.

In AML, AraC treatment induces LAPTM5 upregulation, which promotes *LAMP1/2* transcription and lysosomal biogenesis. This facilitates autophagolysosome formation and enhances autophagic flux to reduce AraC-induced apoptosis, resulting in drug resistance. Targeting LAPTM5 represents a promising strategy to overcome this autophagy-mediated resistance.

## Highlights

- Single-cell analysis identified LAPTM5 as a lysosomal transmembrane protein that drives intrinsic AraC resistance in AML.
- LAPTM5 promotes lysosome-dependent autophagy, thereby conferring AraC resistance in AML cells.
- Targeting LAPTM5 disrupts autophagy and acts synergistically with AraC to suppress AML progression.

## Introduction

Acute myeloid leukemia (AML) is a genetically heterogeneous hematologic malignancy and the most common type of leukemia in adults [1]. Without timely therapeutic intervention, most patients experience aggressive disease progression and survive for less than one year [2, 3]. For over five decades, cytarabine (AraC)-based combination therapy has remained the cornerstone of clinical treatment for AML [4]. Within this regimen, about 60% of patients respond positively to treatment, with induction therapy achieving an 80% complete remission rate. However, the long-term outcomes remain unsatisfactory: only 35–40% of patients under 60 years and 5–15% of patients over 60 achieve extended survival [5–7]. The majority of AML deaths are attributed to chemotherapy resistance [8–11]. Thus, elucidating the mechanisms underlying AraC resistance in AML remains an urgent scientific challenge.

The development of drug resistance in tumor cells arises from the cumulative acquisition of driver gene mutations over an extended period [12, 13]. To clarify the molecular mechanisms underlying drug resistance in AML, it is essential to characterize the temporal gene expression status of AML cells across stages of resistance development. Conventional sequencing technologies operate at the bulk genomic level, in which they capture inter-sample gene expression but mask the inherent heterogeneity of individual cells. In contrast, single-cell RNA sequencing (scRNA-seq) overcomes this limitation by enabling analysis of dynamic biological processes at single-cell resolution [14, 15]. By comparing transcriptional profiles between AraC-sensitive and resistant tumor cell populations using scRNA-seq, we can gain deeper insights into the evolutionary trajectory of drug resistance in AML.

Currently, numerous studies have revealed that the upregulated autophagic activity in AML cells contributes to the development of AraC resistance [16, 17]. Autophagy, an intracellular catabolic process crucial for maintaining cellular homeostasis, has been shown to be associated with drug resistance in various cancers [18–20]. Research has shown that AML cells exhibit metabolic changes following chemotherapy, and increased expression levels of key autophagy-related genes ATG3 and ATG7 are associated with poor clinical prognosis in AML patients. These findings suggest that autophagy supports AML cell proliferation in vitro and promotes AML progression in vivo [21–23]. Consistently, studies have shown that inhibiting the expression of autophagy related genes ATG5 and ATG7 in AML cells enhances the survival rate of AML-bearing mice [24, 25]. Therefore, blocking autophagy holds promise as a new strategy to overcome drug resistance in AML treatment [26]. However, autophagy-blocking drugs often cause severe side effects due to the lack of specific targets. Consequently, there is a need to identify key proteins involved in regulating autophagic activity in drug-resistant cells, which could serve as potential therapeutic targets to overcome AraC resistance.

Here, we identified a significant enrichment of genes upregulated in lysosome-related pathways in AraC-resistant cells compared to their sensitive counterparts based on KEGG analysis. Notably, we identified LAPTM5—a lysosomal transmembrane protein—as a crucial driver of AraC resistance, with its expression markedly elevated in AraC-resistant cells. Functional studies demonstrated that downregulation of LAPTM5 enhanced the sensitivity of AML cells to AraC by disrupting lysosomal homeostasis, thereby blocking autolysosome fusion and reversing chemotherapy resistance. Collectively, these findings highlight LAPTM5 as a promising therapeutic target to improve AraC efficacy in AML.

## Results

### ScRNA-seq analysis identified LAPTM5 as a potential driver of AraC resistance in AML

**Fig. 1:**
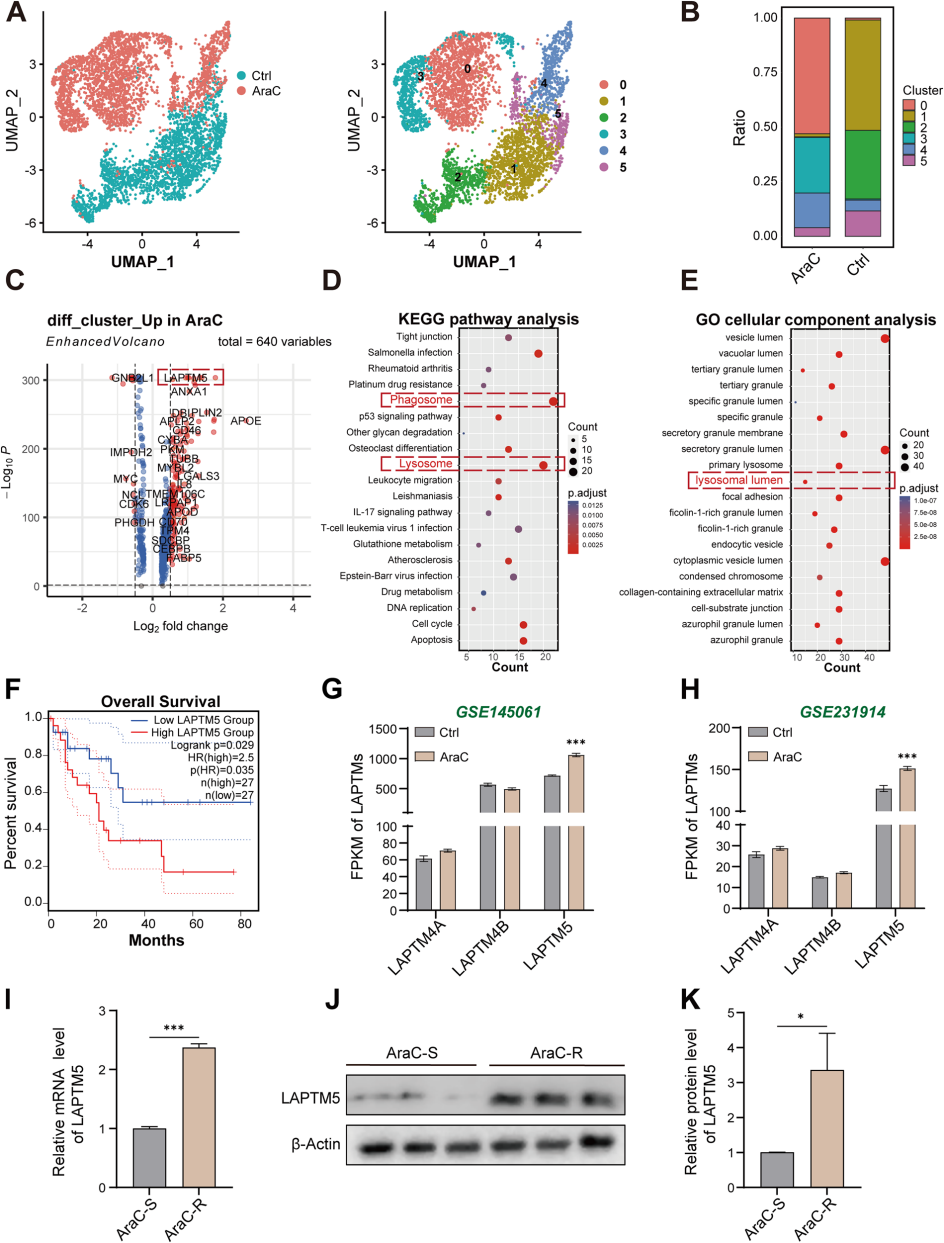
ScRNA-seq analysis identifies LAPTM5 as a potential driver of AraC resistance in AML. **A** Uniform Manifold Approximation and Projection (UMAP) visualization of 4,981 AML cells from the GSE146590 dataset, colored by treatment group (AraC vs. Ctrl). **B** Proportional distribution of cell clusters within AraC-treated and control groups. **C** Volcano plot of differentially expressed genes (DEGs) between AraC-resistant and sensitive clusters. The red box highlights *LAPTM5*. KEGG pathway (**D**) and GO cellular component (**E**) enrichment analyses of genes upregulated in AraC-resistant cells. **F** Kaplan–Meier overall survival analysis of AML patients from the GEPIA2 database, stratified by *LAPTM5* expression levels (high vs. low). **G**, **H** Expression levels of LAPTM5 and its homologs (*LAPTM4A*, *LAPTM4B*) in two independent AML bulk RNA-seq datasets (GSE145061 and GSE231914). **I** RT-qPCR quantification of *LAPTM5* mRNA in AraC-sensitive (AraC-S) and resistant (AraC-R) HL60 cells. *n* = 3. Western blot analysis (**J**) and quantification (**K**) of LAPTM5 protein levels in AraC-S and AraC-R cells. *n* = 3. Data are presented as the means ± SD. *p < 0.05, **p < 0.01, ***p < 0.001. NS not significant.

First, we retrieved single-cell RNA sequencing (scRNA-seq) data from the GEO database (accession GSE146590). The merged dataset comprised a total of 34,738 genes from 6278 AML cells, including those subjected to AraC treatment (AraC) and untreated controls (Ctrl), which included 3480 cells in the AraC group and 2798 cells in the Ctrl group. After rigorous quality control and filtering (see Methods), a total of 4981 high-quality cells with expression data for 32,738 genes were retained for downstream analysis (Supplementary Fig. S1A–D). Principal component analysis (PCA) based on these variable genes revealed 6 distinct clusters among the cells (Fig. 1A). Notably, the AraC-treated and control cells formed largely separate groups, with AraC-surviving cells predominantly enriched in clusters 0 (53%) and 3 (25%), while the untreated cells were mainly found in clusters 1 (51%) and 2 (31%) (Fig. 1B). A small number of both treated and untreated cells were found in clusters 4 and 5, suggesting the presence of intrinsic (primary) resistance in some cells. The top 10 marker genes for each cluster are summarized in Supplementary Fig. S1E.

To investigate the gene expression dynamics associated with AraC resistance, we conducted pseudotime trajectory analysis using the Monocle R package (v2.24.0). Cluster-defining genes were used to construct developmental trajectories, with dimensionality reduction achieved via the DDRTree algorithm. As shown in Supplementary Fig. S2A, B, all cells were distributed in an ordered pattern along the pseudotime axis. The inferred trajectory suggests that cells transition from cluster 2 toward either clusters 0/3 or 4/5. Based on these patterns, we categorized cells into three states: adaptive resistance (clusters 0 and 3), primary resistance (clusters 4 and 5), and drug-sensitive (clusters 1 and 2). Expression trends of top cluster-specific marker genes (Supplementary Fig. S1E) along the pseudotime path were further confirmed by gene expression dynamics (Supplementary Fig. S2C), which aligned well with the resistance progression model.

To further elucidate the molecular drivers of AraC resistance, we conducted differential expression analysis to identify resistance-associated genes. As depicted in Fig. 1C, a total of 640 variable genes were identified. Subsequent Kyoto Encyclopedia of Genes and Genomes (KEGG) pathway and Gene Ontology (GO) cellular component analysis revealed lysosome-related genes were significantly enriched, indicating that lysosomal function or formation was reprogrammed in AraC-R AML cells (Fig. 1D, E). Upon reviewing scRNA-seq data, we found that the LAPTM5 gene was highly expressed in drug-resistant cells (Fig. 1C). LAPTM5 is a transmembrane protein located in late endosomes and lysosomes, involved in protein transport from the Golgi to lysosomes, and affects lysosomes and the cytoplasmic membrane by interacting with the E3 ubiquitin ligase-Nedd4 and mediating protein sorting [27, 28]. These observations led us to hypothesize that LAPTM5 may serve as a critical contributor to AraC resistance mechanisms in AML.

The GEPIA2 analysis revealed that high expression of LAPTM5 was associated with reduced overall survival in AML patients, indicating a correlation between elevated LAPTM5 levels and poor prognosis (Fig. 1F). Further analysis of two independent AML datasets from the GEO database revealed that LAPTM5 expression is significantly upregulated following AraC treatment, while its homologs LAPTM4A and LAPTM4B show no significant changes (Fig. 1G, H). These findings indicated that the LAPTM protein family responds differently to AraC and highlight a specific role for LAPTM5 in the progression of AraC resistance in AML.

To substantiate the role of LAPTM5 in the development of drug resistance, we induced and established a stable AraC-resistant AML cell line (AraC-R). The IC_50_ of AraC in AraC-R cells was more than 10-fold that of sensitive cells (AraC-S) (Supplementary Fig. S3A). Under AraC treatment at 5 μM, the AraC-R cells did not undergo significant cell death (Supplementary Fig. S3B, C). The levels of apoptotic proteins, including cleaved Caspase-3 and the ratio of cleaved to total Poly (ADP-ribose) polymerase (PARP), were significantly reduced in the AraC-R cells (Supplementary Fig. S3D–F). These results collectively indicate that we have successfully established AraC-resistant AML cell lines. Subsequently, LAPTM5 levels were examined in the AraC-R, and it was found that *LAPTM5* mRNA and protein levels were up-regulated 2-fold compared to AraC-S cells (Fig. 1I–K), which was consistent with the results of scRNA-seq.

### Inhibiting LAPTM5 expression enhanced the sensitivity of AML cells to AraC

**Fig. 2:**
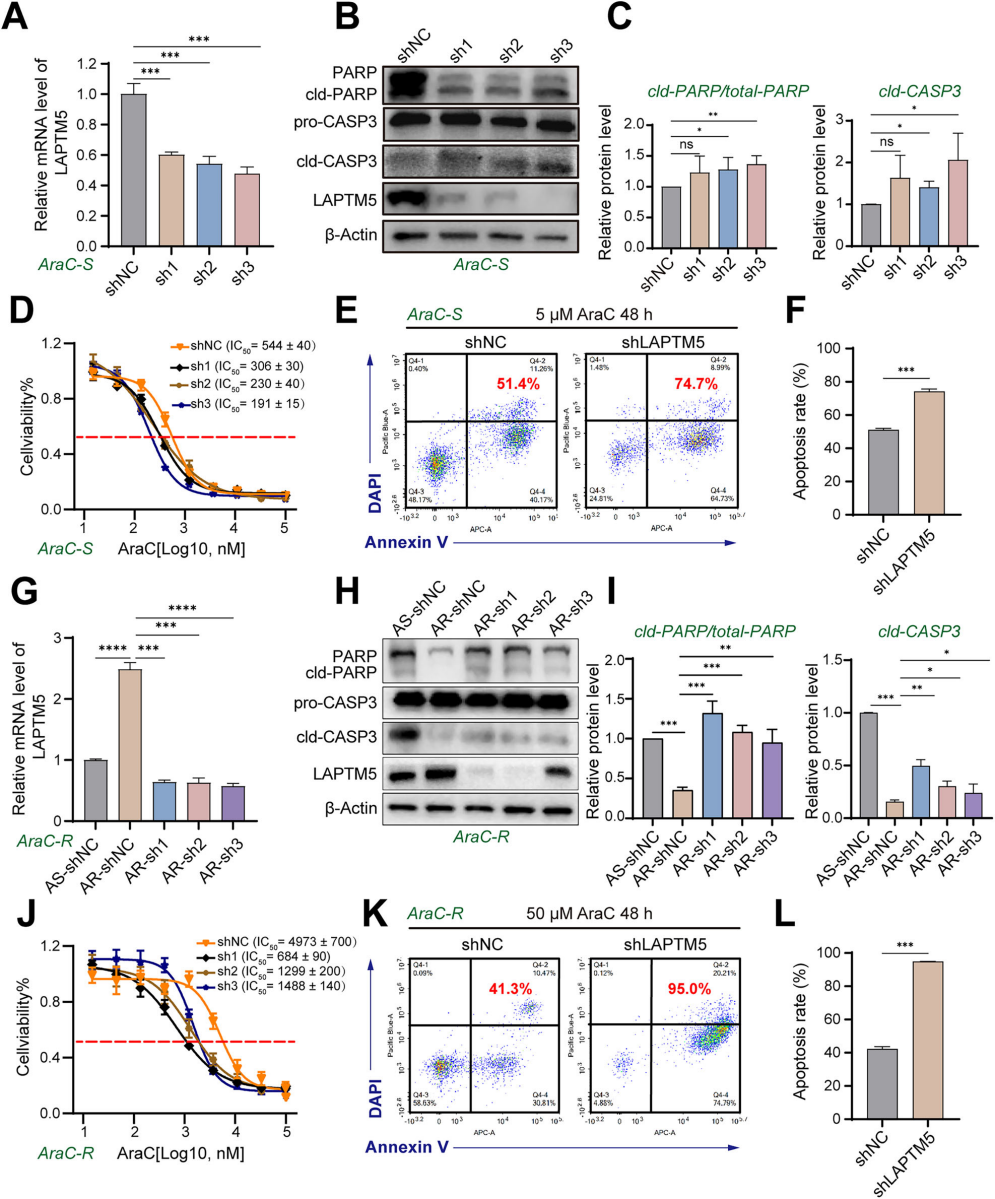
LAPTM5 knockdown sensitizes both sensitive and resistant AML cells to AraC-induced apoptosis. **A** RT-qPCR verification of *LAPTM5* knockdown efficiency in AraC-sensitive (AraC-S) HL60 cells transfected with scramble control (shNC) or *LAPTM5*-targeting shRNAs (sh1, sh2, sh3). *n* = 3. Western blot analysis (**B**) and quantification (**C**) of apoptotic markers (PARP, Caspase-3) in AraC-S cells treated with 5 μM AraC for 48 h. *n* = 3. **D** Dose-response viability curves (CCK-8 assay) of AraC-S cells expressing shNC or shLAPTM5. Statistical analysis of the corresponding IC_50_ values is provided in Supplementary Fig. S4A. Flow cytometry analysis (**E**) and quantification (**F**) of apoptosis (Annexin V/DAPI staining) in AraC-S cells treated with 5 μM AraC for 48 h. *n* = 3. **G** RT-qPCR verification of *LAPTM5* knockdown efficiency in AraC-resistant (AraC-R) HL60 cells. *n* = 3. Western blot analysis (**H**) and quantification (**I**) of apoptotic markers in AraC-R cells treated with 50 μM AraC for 48 h. *n* = 3. **J** Dose-response viability curves of AraC-R cells expressing shNC or shLAPTM5. Statistical analysis of the corresponding IC_50_ values is provided in Supplementary Fig. S4B. Flow cytometry analysis (**K**) and quantification (**L**) of apoptosis in AraC-R cells treated with 50 μM AraC for 48 h. *n* = 3. cld cleaved, pro pro-form, shLAPTM5 short hairpin RNA targeting LAPTM5.

To investigate whether LAPTM5 acts as an active modulator of drug response rather than a passive bystander, we first examined its role in AraC-sensitive (AraC-S) AML cells. This approach allowed us to assess LAPTM5’s contribution to cell survival under chemotherapeutic stress independently of acquired resistance mechanisms. We knocked down LAPTM5 in AraC-S cells using three independent short hairpin RNA (shRNA) constructs. Knockdown efficiency in LAPTM5-deficient sensitive cells (referred to as AS-shLAPTM5 in figures) was validated by RT-qPCR (mRNA) and western blotting (protein) (Fig. 2A, B).

Since the half-maximal inhibitory concentration (IC_50_) is a well-established quantitative metric of drug responsiveness—reflecting the concentration required to reduce cell viability by 50%—changes in IC_50_ values directly indicate altered drug sensitivity. Notably, LAPTM5 silencing caused a pronounced leftward shift of the AraC dose-response curve in AraC-S cells, signifying enhanced susceptibility to AraC (Fig. 2D and Supplementary Fig. S4A). This shift strongly suggested that LAPTM5 contributes to intrinsic drug insensitivity.

Next, we evaluated whether LAPTM5 knockdown sensitizes AML cells to AraC-induced cell death. Live/dead cell staining revealed a marked increase in AraC-induced apoptosis in LAPTM5-knockdown cells compared to controls, indicating a pro-death synergism between LAPTM5 suppression and AraC treatment (Supplementary Fig. S4C). As a pyrimidine analog, AraC exerts cytotoxicity after intracellular metabolism to Ara-CTP, which incorporates into DNA, blocks replication, induces DNA damage, and ultimately triggers apoptosis. Consistent with this mechanism, we analyzed apoptotic markers post-AraC treatment. Western blotting showed elevated levels of cleaved PARP (cld-PARP) and cleaved Caspase-3 (cld-CASP3)—hallmark apoptotic effectors—with signal intensities increasing by >2-fold upon LAPTM5 knockdown (Fig. 2B, C). These findings confirm enhanced apoptosis and biochemically correlate with reduced IC_50_ values, reinforcing a mechanistic link between LAPTM5 expression and the apoptotic threshold in AraC-exposed cells. Flow cytometry-based apoptosis assays further validated these results, demonstrating a 1.5-fold increase in apoptotic cells in LAPTM5-knockdown cells relative to controls (Fig. 2E, F).

Having established a baseline function of LAPTM5 in AraC-S cells, we next extended our investigation to AraC-R cells to explore its contribution to acquired resistance. In parallel, we generated LAPTM5-knockdown AraC-R HL60 cell lines (referred to as AR-shLAPTM5 in figures) using three distinct shLAPTM5 constructs (AR-sh1, AR-sh2, AR-sh3), with knockdown efficiency confirmed by RT-qPCR (Fig. 2G). Similar to AraC-S cells, LAPTM5 knockdown in AraC-R cells significantly reduced IC_50_ (Fig. 2J and Supplementary Fig. S4B), upregulated apoptotic markers (Fig. 2H, I), and increased apoptotic cell proportions (Fig. 2K, L), indicating effective re-sensitization to AraC. Strikingly, AraC-induced apoptosis in LAPTM5-knockdown AraC-R cells was approximately 2-fold higher than in LAPTM5-knockdown AraC-S cells, suggesting a heightened dependency on LAPTM5 for survival in the resistant context. These data demonstrate that LAPTM5 inhibition enhances AraC sensitivity in both sensitive and resistant AML cells, with a more profound synergistic cytotoxic effect in AraC-R cells—highlighting LAPTM5’s critical role in maintaining the drug-tolerant state of resistant clones.

To validate specificity, we performed reciprocal overexpression experiments. Ectopic LAPTM5 expression in HL60 cells significantly increased AraC IC_50_ and attenuated drug-induced apoptosis, further supporting its pro-survival role in AML (Supplementary Fig. S4D, E). To confirm generalizability across genetic backgrounds, we repeated knockdown and overexpression experiments in THP1 cells—a monocytic AML subtype distinct from the promyelocytic HL60. Results in THP1 cells mirrored those in HL60 (Supplementary Fig. S4F–H), indicating that LAPTM5-mediated modulation of AraC response is conserved across AML subtypes and underscoring the robustness of our findings.

Collectively, coordinated changes in IC_50_ values, apoptosis rates, and key apoptotic markers (cld-PARP, cld-CASP3) consistently implicate LAPTM5 as a key negative regulator of AraC-induced apoptosis, thereby promoting drug resistance. Targeting LAPTM5 may thus represent a promising strategy to overcome chemoresistance and restore drug sensitivity in refractory AML.

### LAPTM5 enhances autophagy in AML cells and contributes to the development of AraC resistance

**Fig. 3:**
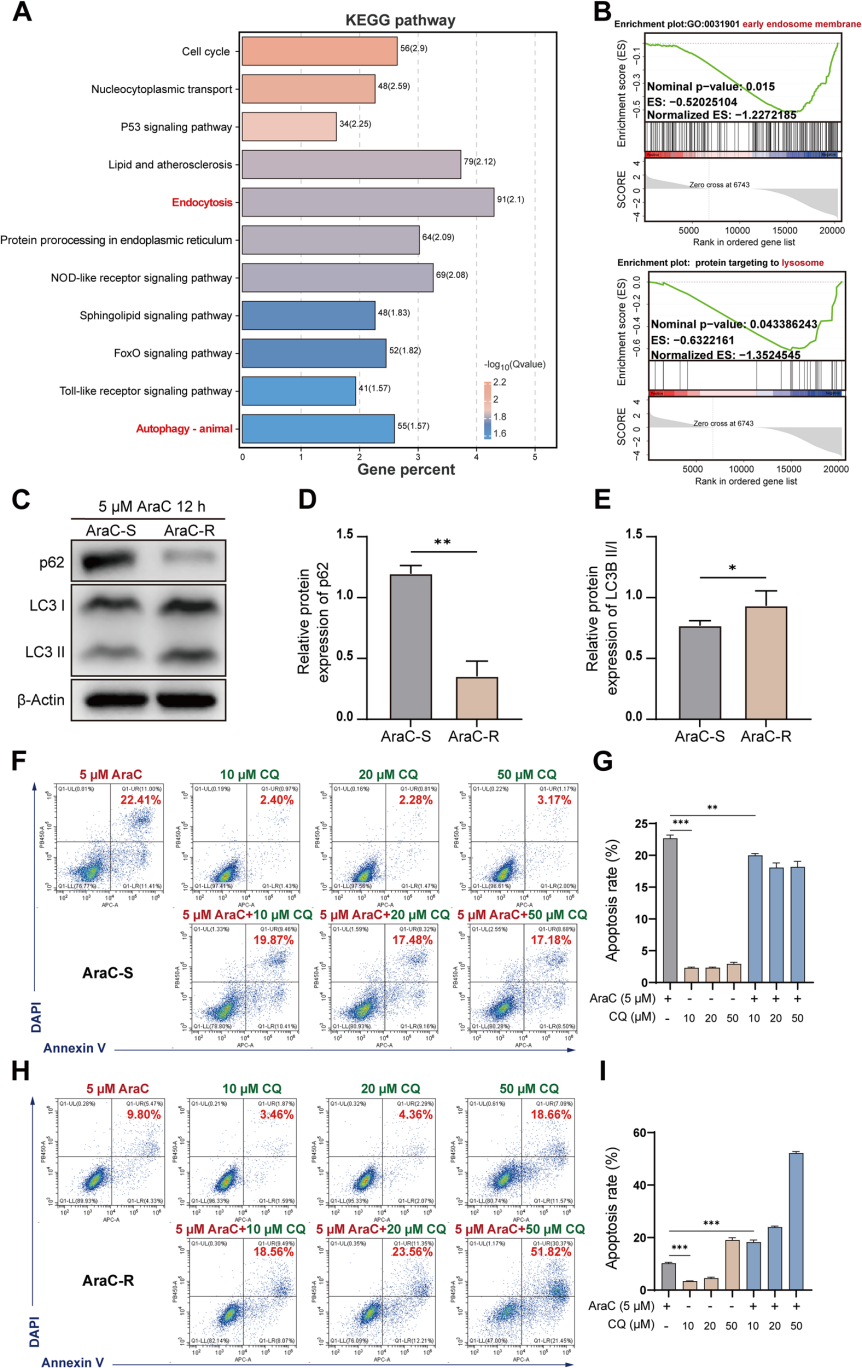
Enhanced autophagy drives AraC resistance in AML cells. **A** KEGG pathway enrichment analysis of genes significantly downregulated in *LAPTM5*-knockdown AraC-R cells compared to scramble control. **B** Gene Set Enrichment Analysis (GSEA) of gene sets related to the early endosome membrane and protein targeting to lysosomes in the *LAPTM5*-knockdown group. Western blot analysis (**C**) and quantification of p62 (**D**) and LC3-II/LC3-I ratio (**E**) in AraC-S and AraC-R cells treated with 5 μM AraC for 12 h. *n* = 3. Flow cytometric analysis (**F**, **H**) and quantification of apoptosis (**G**, **I**) in AraC-S and AraC-R cells treated with 5 μM AraC alone or in combination with Chloroquine (CQ) at indicated concentrations (10, 20, 50 μM) for 48 h. *n* = 3.

To explore how LAPTM5 contributes to AraC resistance, we performed RNA sequencing on AraC-R cells following LAPTM5 knockdown. Principal component analysis (PCA) revealed distinct gene expression profiles in LAPTM5-depleted cells compared to scramble controls (Supplementary Fig. S5A), indicating broad transcriptional remodeling induced by LAPTM5 silencing. To confirm target specificity and exclude off-target effects on homologs, we assessed LAPTM4A and LAPTM4B expression, which remained unchanged upon LAPTM5 knockdown (Supplementary Fig. S5B). Differential expression analysis identified 4,533 genes significantly downregulated upon LAPTM5 knockdown compared to scramble controls (Supplementary Fig. S5C). KEGG enrichment revealed that downregulated genes were mainly involved in cell cycle regulation, suggesting that LAPTM5 may influence AML cell proliferation through cell cycle control (Fig. 3A). Notably, autophagy-related pathways were also enriched (Fig. 3B). As lysosomal membrane proteins frequently regulate drug resistance via intracellular trafficking, drug sequestration, and stress responses, with autophagy emerging as an evolutionarily conserved survival mechanism under metabolic or drug-induced stress, we hypothesized autophagy as a critical mediator of LAPTM5-dependent AraC resistance.

To evaluate autophagy’s role in AraC resistance, we first compared autophagic activity between AraC-sensitive (AraC-S) and AraC-R HL60 cells. Autophagy is characterized by autophagosome formation, during which cytosolic LC3-I undergoes lipidation to form LC3-II (a marker of autophagosome membranes), and degradation of p62 (a selective autophagy substrate degraded in autolysosomes). Western blotting showed a higher LC3-II/LC3-I ratio and reduced p62 levels in AraC-R cells relative to AraC-S cells (Fig. 3C–E), indicating enhanced autophagic flux. Concordantly, AraC-R cells exhibited reduced apoptosis (9.80%) compared to AraC-S cells (22.41%) following AraC treatment (Fig. 3F–I). To test if autophagy directly protects against AraC-induced death, we treated cells with chloroquine (CQ), an autophagy inhibitor that blocks autophagosome-lysosome fusion. CQ alone moderately increased apoptosis in AraC-R cells (18.66%) but had minimal effect on AraC-S cells (3.17%). Co-treatment with AraC and CQ further elevated apoptosis in AraC-R cells to 51.82% (Fig. 3F–I), confirming autophagy’s protective role in AraC resistance. These findings were robustly validated in the THP1 model (Supplementary Fig. S6). While the combination of CQ and AraC did not significantly enhance cytotoxicity in THP1 AraC-S cells compared to AraC alone (indicating a lack of autophagy dependency in sensitive cells), it triggered a synergistic surge in apoptosis in THP1 AraC-R cells (reaching ~43.55%), effectively overcoming the acquired resistance.

**Fig. 4:**
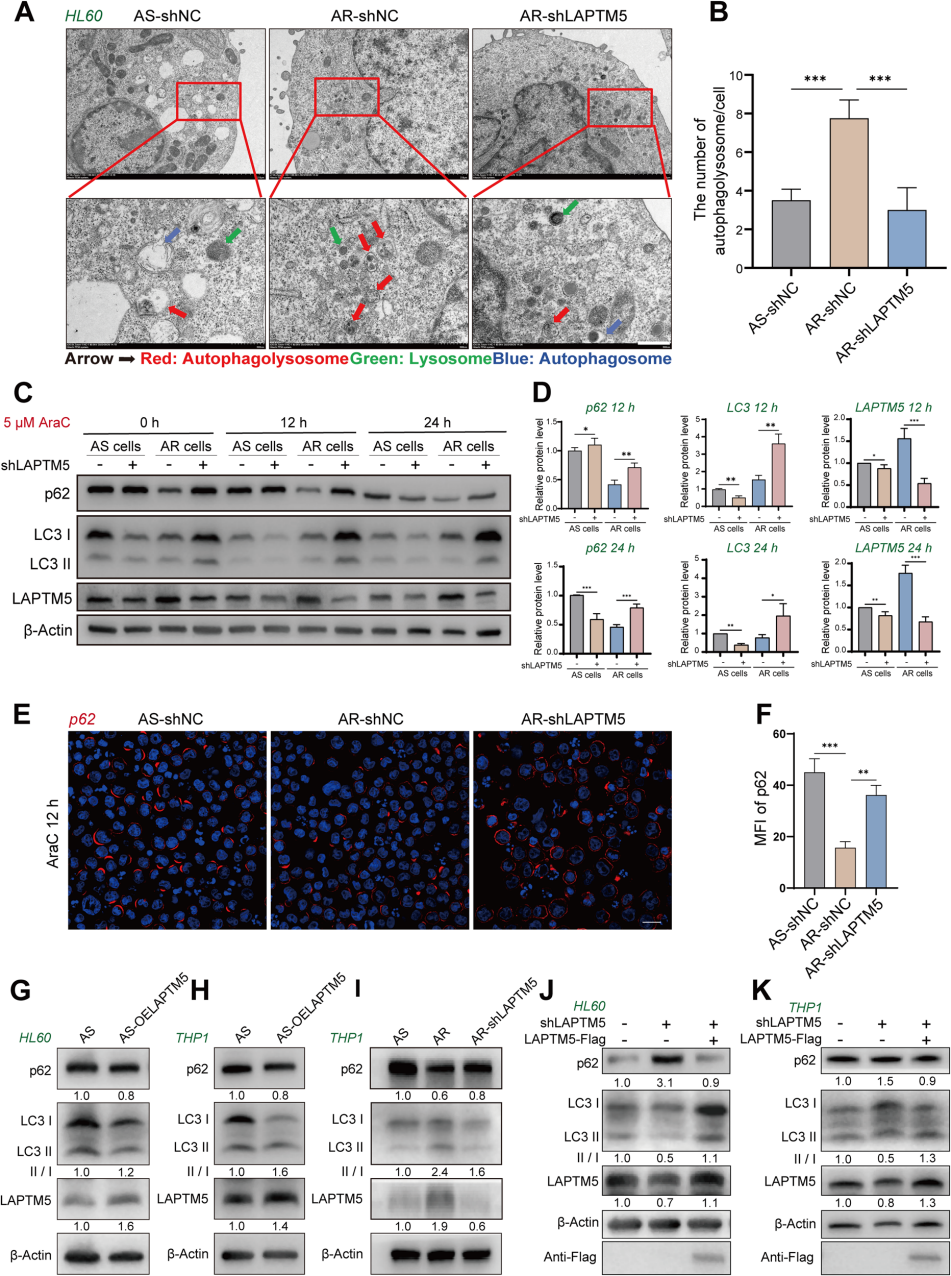
LAPTM5 enhances autophagy in AML cells and contributes to the development of AraC resistance. Representative Transmission Electron Microscopy (TEM) images (**A**) and quantification (**B**) of autophagic structures in HL60 AraC-S (shNC), AraC-R (shNC), and LAPTM5-knockdown AraC-R cells (AR-shLAPTM5). Red arrows indicate autophagolysosomes; green arrows indicate lysosomes; blue arrows indicate autophagosomes. *n* = 3. Scale bars: 500 nm. Western blot analysis (**C**) and quantification (**D**) of p62, LC3, and LAPTM5 protein levels in HL60 cell lines treated with 5 μM AraC for 0, 12, and 24 h. *n* = 3. Immunofluorescence (IF) staining (**E**) and mean fluorescence intensity (MFI) quantification (**F**) of p62 (red) in HL60 cell lines treated with 5 μM AraC for 12 h. Nuclei were stained with DAPI (blue). *n* = 3. Scale bars: 20 μm. **G** Western blot analysis and quantification of p62, LC3, and LAPTM5 in HL60 AraC-S cells overexpressing LAPTM5 (AS-OELAPTM5). *n* = 3. **H** Western blot analysis and quantification of p62, LC3, and LAPTM5 in THP1 AraC-S cells overexpressing LAPTM5 (AS-OELAPTM5). *n* = 3. **I** Western blot analysis and quantification of p62, LC3, and LAPTM5 protein levels in THP1 AraC-sensitive (AS), AraC-resistant (AR), and LAPTM5-knockdown AraC-resistant (AR-shLAPTM5) cells. *n* = 3. **J**, **K** Rescue experiments validating the regulation of autophagic flux by LAPTM5. Western blot analysis of p62, LC3, and LAPTM5 levels in AraC-resistant HL60 (**J**) and THP1 (**K**) cells transduced with shNC, shLAPTM5, or co-transduced with shLAPTM5 and a Flag-tagged LAPTM5 overexpression vector (shLAPTM5 + OELAPTM5). The numbers below the bands indicate the relative densitometric quantification normalized to control. MFI mean fluorescence intensity, OE overexpression, shLAPTM5 short hairpin RNA targeting LAPTM5.

To determine if LAPTM5 directly modulates autophagy, transmission electron microscopy (TEM) was used to quantify autophagic structures. AraC-R cells displayed a 3-fold increase in autophagosomes and lysosomes compared to AraC-S cells (Fig. 4A, B). Importantly, LAPTM5 knockdown in AraC-R cells led to a marked reduction in autolysosomes (Fig. 4A, B), indicating impaired late-stage autophagy. To investigate the dynamic impact of LAPTM5 on autophagic flux, we analyzed the levels of LAPTM5, LC3, and p62 at both 12 h and 24 h post-AraC treatment (Fig. 4C, D). Time-course analysis showed that LAPTM5 expression was consistently higher in AraC-R cells compared to AraC-S cells throughout the 24 h AraC treatment. At 12 h, LAPTM5 knockdown significantly blocked autophagic flux in AraC-R cells, evidenced by the marked accumulation of p62 and LC3 compared to control cells. This confirms that LAPTM5 is essential for maintaining efficient autophagic turnover under drug stress. By 24 h, while LAPTM5 levels remained consistently suppressed in knockdown cells—highlighting the sustained silencing efficiency—the profile of autophagy markers shifted. In the sensitized cells (AS-shLAPTM5), p62 levels decreased sharply compared to the 12 h time point. This decline likely reflects global protein degradation due to cell lysis or potential p62 cleavage by caspases associated with the initiation of irreversible cell death, rather than a restoration of autophagic flux. Collectively, these data demonstrate that LAPTM5 inhibition initially blocks autophagic flux (12 h), thereby accelerating the transition to apoptotic cell death (24 h). Immunofluorescence analysis further corroborated this blockage, revealing a striking accumulation of cytoplasmic p62 puncta in LAPTM5-depleted cells. This observation was quantitatively supported by a significant increase in the mean fluorescence intensity (MFI) of p62 compared to controls, reflecting defective autophagic clearance (Fig. 4E, F).

To validate the role of LAPTM5 in regulating autophagy, we overexpressed LAPTM5 in two wild-type AML cell lines. In both cell models, LAPTM5 overexpression reduced p62 levels and increased the LC3-II/LC3-I ratio, indicating enhanced autophagy (Fig. 4G, H). Conversely, knockdown of LAPTM5 in THP1 AraC-R cells increased p62 accumulation and decreased the LC3-II/LC3-I ratio (Fig. 4I), reflecting a blockade in autophagic flux. To confirm that the observed impairment in autophagy and increased sensitivity to AraC were specifically caused by LAPTM5 loss, we performed rescue experiments by re-expressing Flag-tagged LAPTM5 in knockdown cells. Western blot analysis showed that LAPTM5 re-expression effectively reversed the autophagic defect, as evidenced by the clearance of accumulated p62 and the restoration of LC3 turnover (Fig. 4J, K). Furthermore, functional rescue assays demonstrated that re-introducing LAPTM5 significantly attenuated AraC-induced apoptosis compared to the knockdown group (Supplementary Fig. S7). These findings confirm that LAPTM5 is essential for sustaining autophagic flux and conferring chemotherapeutic resistance.

Finally, to functionally validate that the survival advantage conferred by LAPTM5 is strictly dependent on this enhanced autophagic flux, we performed rescue experiments using the autophagy inhibitor chloroquine (CQ) in LAPTM5-overexpressing cells (HL60-OE and THP-1-OE) (Supplementary Fig. S8). While LAPTM5 overexpression significantly attenuated AraC-induced apoptosis compared to wild-type controls, co-treatment with 50 µM CQ significantly overcame this protective effect. The combination treatment elicited an apoptotic response that was statistically superior to either agent alone (*p* < 0.05) and exceeded the sum of their individual effects, confirming that LAPTM5-driven resistance relies on autophagy. Notably, although CQ successfully re-sensitized these acutely overexpressing models (reaching ~43% apoptosis; Supplementary Fig. S8), the magnitude of apoptosis was lower than that observed in the acquired resistance models (reaching ~51.8%; Fig. 3). This distinction likely arises because LAPTM5 overexpression in naive cells provides a supplementary survival advantage, and its inhibition merely reverts the cells to their baseline sensitivity. In contrast, resistant cells have likely undergone fundamental metabolic rewiring during long-term selection, evolving an obligatory reliance (‘addiction’) on autophagy. Consequently, lysosomal blockade in resistant cells triggers a catastrophic system collapse that exceeds simple re-sensitization.

Collectively, these findings demonstrate that LAPTM5 promotes autophagy in AML cells, particularly under drug stress. By supporting late-stage autophagic flux, LAPTM5 enhances cell survival during chemotherapy, thereby contributing to AraC resistance.

### LAPTM5 drives transcriptional lysosomal biogenesis to facilitate autophagy

Based on our observations that LAPTM5 depletion suppresses lysosome-related gene signatures (Fig. 3B) and reduces lysosomal accumulation in AraC-resistant cells (Fig. 4A, B), we hypothesized that LAPTM5 regulates autophagy by directly maintaining lysosomal homeostasis. Given that LAPTM5 is a lysosomal transmembrane protein localized to late endosomes and lysosomes, which are key organelles responsible for the final degradation step in autophagy, we therefore sought to dissect whether LAPTM5 modulates lysosomal abundance and integrity, thereby contributing to the elevated autophagic activity observed in resistant cells.

**Fig. 5:**
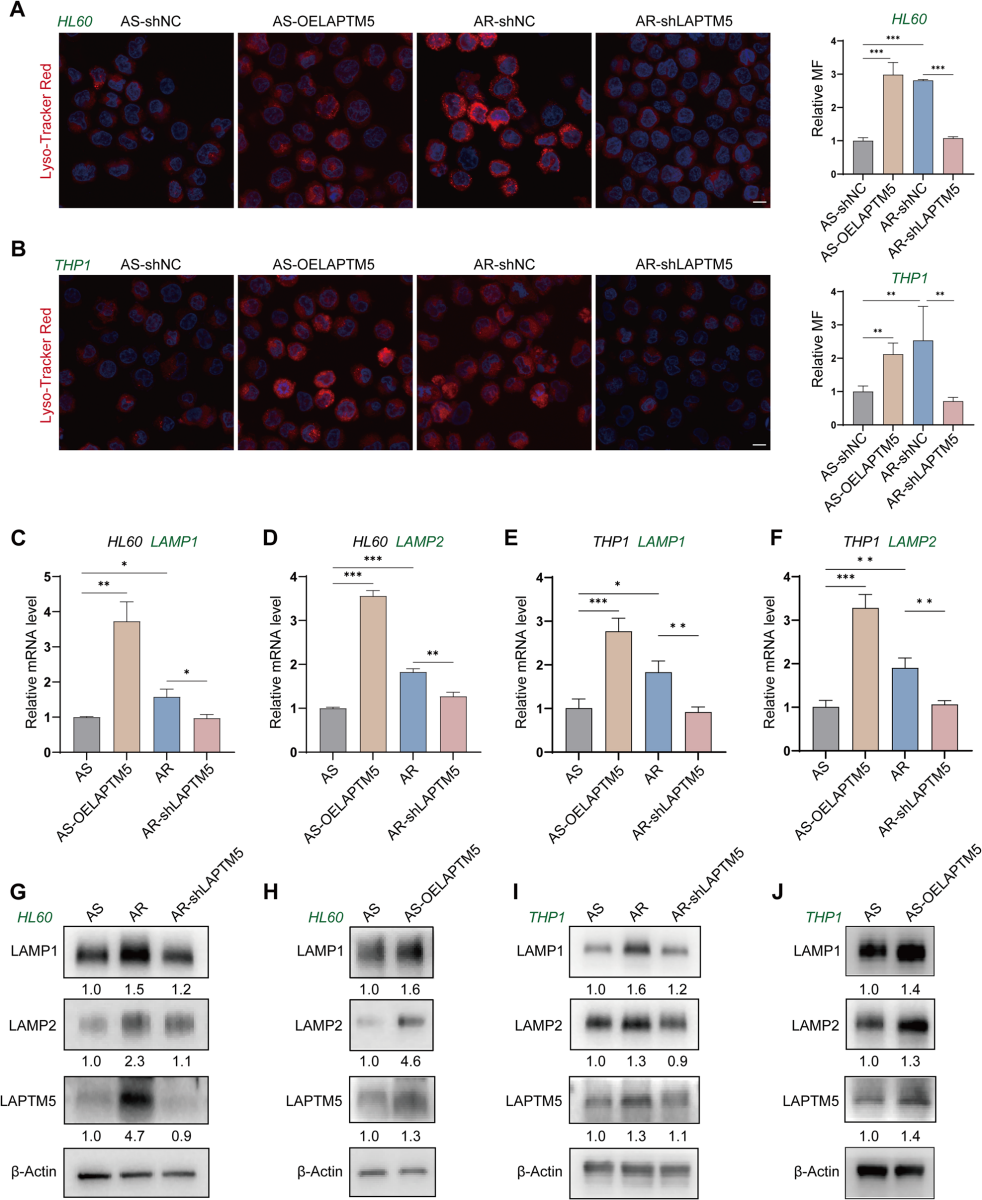
LAPTM5 promotes lysosomal biogenesis to facilitate autophagy. Confocal Laser Scanning Microscopy (CLSM) analysis of lysosome distribution and abundance using Lyso-Tracker Red staining in HL60 (**A**) and THP1 (**B**) cell lines. *n* = 3. Scale bars: 10 μm. RT-qPCR quantification of *LAMP1* (**C**) and *LAMP2* (**D**) mRNA levels in HL60 cells. *n* = 3. RT-qPCR quantification of *LAMP1* (**E**) and *LAMP2* (**F**) mRNA levels in THP1 cells. *n* = 3. Western blot analysis of lysosomal membrane proteins LAMP1 and LAMP2 in HL60 (**G**, **H**) and THP1 (**I**, **J**) cells under indicated conditions, including AraC-sensitive (AS), AraC-resistant (AR), LAPTM5-knockdown (AR-shLAPTM5), and LAPTM5-overexpressing (AS-OELAPTM5) cells. *n* = 3. CLSM confocal laser scanning microscopy, OE overexpression, shLAPTM5 short hairpin RNA targeting LAPTM5.

To further validate these ultrastructural findings in living cells, we stained intracellular lysosomes with a red fluorescent probe and visualized them using confocal laser scanning microscopy (CLSM). In untreated AraC-S cells, lysosomes appeared as sparse red punctate structures with low fluorescence intensity, consistent with a relatively limited lysosomal compartment under basal conditions (Fig. 5A, B). In contrast, AraC-R cells exhibited a marked increase in both the quantity and fluorescence intensity of lysosomal puncta, indicating significant expansion of the lysosomal compartment (Fig. 5A, B). This observation suggests that lysosomal accumulation represents an adaptive response to sustained chemotherapeutic stress. Notably, LAPTM5-knockdown AraC-R cells showed a significant reduction in both lysosome number and fluorescence intensity compared to parental AraC-R cells. Conversely, LAPTM5 overexpression in wild-type AML cells further increased lysosomal abundance and fluorescence intensity beyond that observed in AraC-S cells. These findings establish a direct correlation between LAPTM5 expression and lysosomal abundance.

To elucidate the molecular mechanism underlying this regulation, we examined the expression of lysosomal-associated membrane proteins 1 and 2 (LAMP1 and LAMP2). Given that LAMP1 and LAMP2 are the major constituents of the lysosomal membrane that maintain its structural integrity and facilitate autophagosome-lysosome fusion, they serve as critical markers for lysosomal biogenesis and function. To ensure the robustness of our findings, we performed parallel analyses in both HL60 and THP1 cell lines. RT-qPCR analysis first revealed that LAPTM5 modulation significantly altered the transcriptional levels of LAMP1 and LAMP2 (Fig. 5C–F). Specifically, LAPTM5 overexpression markedly elevated, while LAPTM5 knockdown significantly suppressed, the mRNA abundance of these markers in both cell lines. Consistent with the transcriptional changes, Western blotting confirmed a concomitant alteration in protein levels (Fig. 5G–J). This synchronized upregulation of mRNA and protein indicates that LAPTM5 regulates lysosomal homeostasis primarily at the transcriptional level, driving lysosomal biogenesis rather than merely stabilizing lysosomal membrane proteins. By promoting the biogenesis of these structural components, LAPTM5 ensures lysosomal capacity and stability under chemotherapeutic stress, thereby enabling efficient autophagic flux and promoting AML cell survival during cytotoxic stress.

### Targeting LAPTM5 suppressed AML progression in SCID xenograft models

**Fig. 6:**
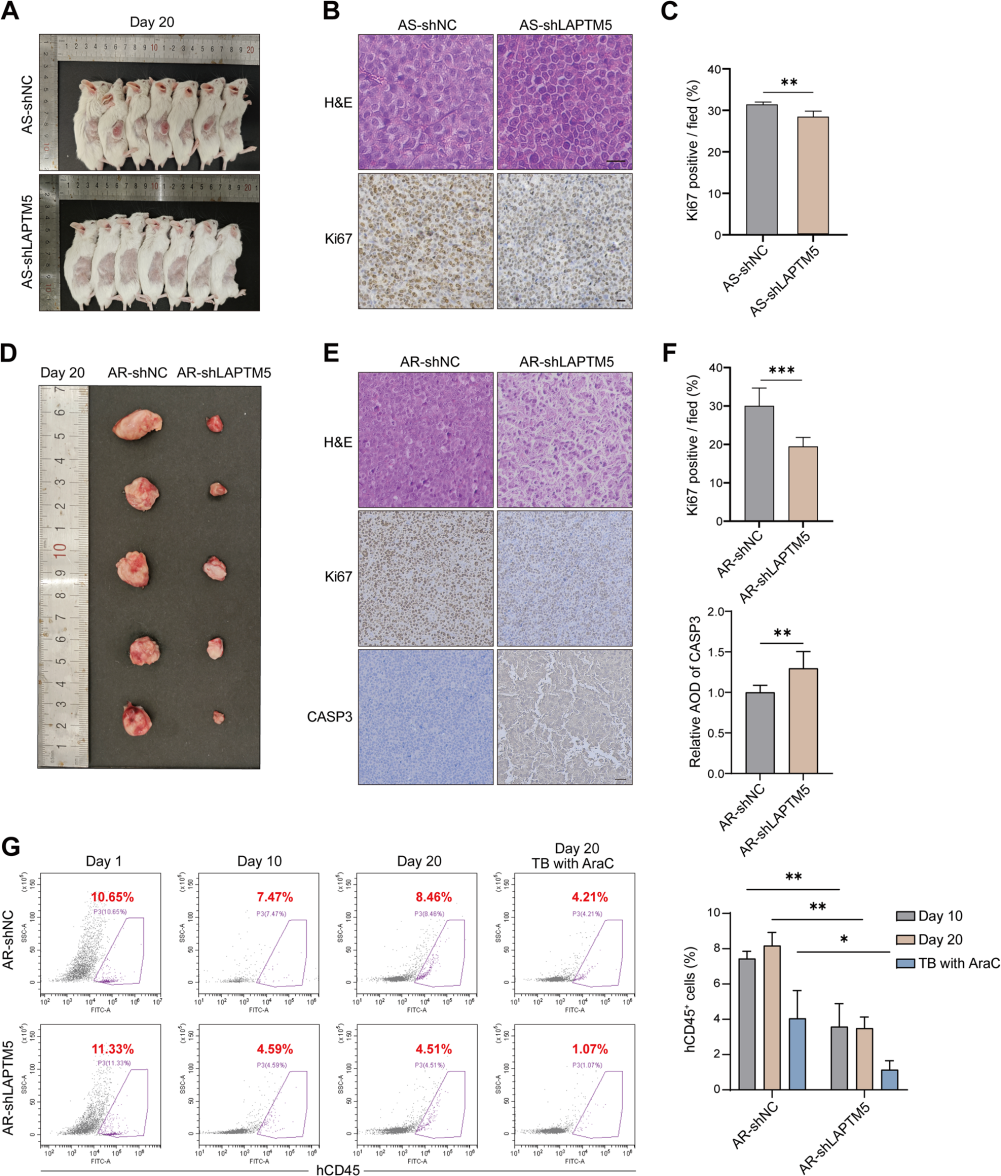
Targeting LAPTM5 suppresses AML progression in SCID xenograft models. **A** Representative images of subcutaneous tumors harvested on day 20 from SCID mice inoculated with HL60 AraC-S cells expressing shNC or shLAPTM5. *n* = 7. Representative hematoxylin and eosin (H&E) staining and Ki67 immunohistochemistry (IHC) of tumor sections (**B**), and quantification of Ki67-positive cells (**C**) in the AraC-S model. *n* = 7. Scale bars: 20 μm. **D** Representative images of subcutaneous tumors harvested on day 20 from the HL60 AraC-R xenograft model treated with AraC (12 mg/kg). *n* = 5. Representative H&E, Ki67, and cleaved Caspase-3 IHC staining of tumor sections (**E**), with quantification of Ki67 positivity and relative AOD of Caspase-3 (**F**) in the AraC-R model. *n* = 5. Scale bars: 50 μm. **G** Flow cytometric quantification of human CD45^+^ (hCD45^+^) cells in the peripheral blood of mice in the hematologic AML xenograft model. Data were collected at days 1, 10 (pre-treatment), and day 20. For day 20, mice were analyzed in two subgroups: untreated (‘Day 20’) and treated with AraC (‘TB with AraC’) from day 11 to 17. *n* = 5. AOD average optical density, TB treated by.

To validate the therapeutic potential of targeting LAPTM5 and extend our in vitro observations, we established AML xenograft models in severe combined immunodeficient (SCID) mice using both AraC-S and AraC-R cells. In the AraC-S model, SCID mice were subcutaneously inoculated with HL60 AraC-S cells expressing either scramble control shRNA (AS-shNC) or LAPTM5-targeting shRNA (AS-shLAPTM5) at a dose of 1 × 10^6^ cells per mouse. Notably, by day 10 post-injection, palpable tumors were observed in the AS-shNC group, whereas no visible tumor growth was detected in the AS-shLAPTM5 group. By day 20, all mice in the AS-shNC group developed tumors (100% incidence), while tumor incidence in the AS-shLAPTM5 group was markedly reduced to 14.3% (Fig. 6A), indicating that LAPTM5 depletion impairs in vivo tumor initiation. For the few tumors that formed in the AS-shLAPTM5 group, tumor growth rate and volume were significantly reduced compared to controls (Supplementary Fig. S9A). Consistently, immunohistochemical (IHC) analysis revealed a notable reduction in the expression of the proliferation marker Ki67 in shLAPTM5 tumors (Fig. 6B, C), supporting the notion that LAPTM5 contributes to AML cell proliferation in vivo.

To extend these findings to AraC-R cells, we established subcutaneous xenografts with HL60 AraC-R cells (AR-shNC *vs*. AR-shLAPTM5) (Supplementary Fig. S9B). As expected, AR-shLAPTM5 tumors grew significantly slower than AR-shNC tumors (Supplementary Fig. S9C), highlighting LAPTM5’s role in driving the aggressive growth of AraC-resistant cells. Upon AraC treatment, AR-shNC tumors continued to grow rapidly. In contrast, AR-shLAPTM5 tumors exhibited a transient increase in volume during the first 3 days of treatment, followed by a gradual reduction after day 3 (Supplementary Fig. S9C and Fig. 6D). Ultimately, the final tumor burden in the knockdown group was reduced by >90% compared to controls, indicating that LAPTM5 knockdown significantly sensitizes resistant cells to AraC in vivo. Supporting this, IHC analysis showed further reduced Ki67 expression in AR-shLAPTM5 tumors (Fig. 6E, F), reflecting suppressed proliferation, and a substantial increase in cleaved Caspase-3 levels, consistent with enhanced apoptotic activity in LAPTM5-knockdown tumors under chemotherapeutic stress.

To mimic the systemic nature of AML, we established a disseminated model by tail vein injection of HL60 cells into SCID mice (Supplementary Fig. S9E). Disease progression was monitored via flow cytometric quantification of human CD45^+^ (hCD45^+^) cells in peripheral blood. On day 1 post-transplantation, comparable frequencies of hCD45^+^ cells were detected across groups (Fig. 6G), confirming successful engraftment. By day 10, leukemia burden in the AR-shNC group was significantly higher than in the AR-shLAPTM5 group (7.47% *vs*. 4.59%, *p* < 0.05) (Fig. 6G), indicating compromised survival of LAPTM5-knockdown leukemia cells in vivo. Starting on day 11, mice were divided into untreated and AraC-treated cohorts. By day 20, the leukemia burden in the untreated cohorts continued to progress. In contrast, AraC treatment markedly reduced the hCD45^+^ cell proportions in both groups. Notably, the AR-shLAPTM5 group treated with AraC exhibited the lowest leukemia burden compared to the treated control group (1.07% vs. 4.21%) and their untreated counterparts (4.51%), validating that LAPTM5 depletion significantly enhances AraC efficacy in vivo (Fig. 6G).

Collectively, these data demonstrate that LAPTM5 promotes AML progression and chemoresistance in vivo. Depletion of LAPTM5 attenuates tumor growth, and enhances AraC efficacy in both subcutaneous and systemic models, supporting LAPTM5 as a promising therapeutic target for AML.

## Discussion and conclusion

Cytarabine (AraC), a cornerstone of AML chemotherapy, exerts cytotoxicity by inhibiting DNA synthesis [29]; however, acquired resistance to AraC remains a major barrier to curative outcomes, with most refractory patients experiencing disease progression and poor survival [30]. While targeted therapies (e.g., FLT3 or BCL-2 inhibitors) have improved precision in subset-specific AML [31, 32], their efficacy is often limited by intrinsic or adaptive resistance, necessitating the identification of novel molecular drivers of treatment failure. Here, we identify LAPTM5 as a critical regulator of AraC resistance in AML, highlighting its potential as a therapeutic target to overcome chemoresistance.

To dissect the molecular basis of AraC resistance, we reanalyzed single-cell RNA sequencing (scRNA-seq) data from AML patients treated with AraC (GEO database), focusing on transcriptional differences between resistant and sensitive leukemic blasts. This unbiased approach revealed enrichment of lysosome-related pathways in resistant cells, with LAPTM5—a lysosomal transmembrane protein—emerging as a top candidate. While LAPTM5 has been implicated in drug resistance in solid tumors (e.g., hepatocellular carcinoma sensitivity to Lenvatinib [33], renal cancer cell self-renewal [34]), and lung adenocarcinoma progression [35], emerging evidence suggests a conserved role in modulating therapeutic sensitivity across different contexts, including hematologic malignancies. Notably, recent studies have demonstrated that LAPTM5 confers resistance to venetoclax in multiple myeloma by promoting autophagosome-lysosome fusion [36], and mediates cisplatin resistance in non-small cell lung cancer (NSCLC) by suppressing LAMP1 ubiquitination to stabilize lysosomal membranes and sustain autophagic flux [37]. These observations strongly corroborate our findings in AML, where we identified a similar LAPTM5-dependent mechanism involving lysosomal maintenance and autophagic flux support. By establishing LAPTM5 as a key mediator of AraC resistance in AML, our study extends its functional relevance beyond solid tumors and immune cells (e.g., B cells [38, 39], T cells [40, 41], macrophages [42]) to malignant hematopoiesis, and highlights LAPTM5 as a universal guardian of lysosomal integrity under chemotherapeutic stress.

However, the precise upstream mechanisms driving LAPTM5 overexpression upon AraC exposure remain to be fully elucidated. Our pathway analysis revealed a significant enrichment of stress-response signaling, including p53 and FoxO pathways, in AraC-resistant cells. Given that AraC induces DNA damage, it is plausible that LAPTM5 upregulation is part of a broad adaptive transcriptional program driven by these stress sensors, or potentially through the activation of master lysosomal regulators. For instance, the Transcription Factor EB (TFEB) is known to translocate to the nucleus in response to lysosomal stress or chemotherapy to activate the coordinated lysosomal expression and regulation gene network [43]. It is likely that AraC treatment triggers a TFEB-mediated transcriptional program that includes LAPTM5 to compensate for lysosomal stress. Alternatively, epigenetic remodeling or the downregulation of specific microRNAs that repress LAPTM5 could also contribute to its accumulation in resistant cells, a hypothesis consistent with the high epigenetic plasticity observed in relapsed AML. Elucidating the detailed upstream regulatory network linking drug-induced stress to LAPTM5 expression remains an important subject for future investigation. Crucially, regardless of the specific upstream driver, the functional consequence of this stress-induced LAPTM5 accumulation appears to be the reinforcement of lysosomal capacity to support high levels of autophagy, thereby countering AraC-induced cytotoxicity.

Mechanistically, our study clarifies that LAPTM5 regulates lysosomal homeostasis primarily at the transcriptional level. We observed that LAPTM5 modulation resulted in concomitant changes in both the mRNA and protein levels of LAMP1 and LAMP2 across multiple cell lines (HL60 and THP1) (Fig. 5). This suggests that LAPTM5 triggers a signaling cascade that activates the transcriptional program of lysosomal biogenesis, rather than merely stabilizing lysosomal membrane proteins post-translationally. Nevertheless, targeting LAPTM5 post-translational stability offers an alternative therapeutic avenue. Previous reports identified the HIV-1 viral protein R (Vpr) as a specific negative regulator that induces LAPTM5 degradation via the lysosomal pathway in macrophages [44]. Consistent with this, in our unpublished studies, we found that ectopic Vpr expression in AraC-resistant AML cells led to a marked reduction in LAPTM5 protein levels and restored AraC sensitivity (data not shown). These findings provide proof of concept that while LAPTM5 is upregulated transcriptionally to drive resistance, its protein stability remains a critical vulnerability amenable to targeted strategies (e.g., gene therapy or small-molecule inhibitors).

Adaptive upregulation of autophagy is a well-documented hallmark of AML chemoresistance, enabling cells to survive under chemotherapy-induced stress by degrading and recycling damaged organelles and proteins [45, 46]. Our mechanistic studies demonstrate that LAPTM5 promotes AraC resistance by sustaining late-stage autophagic flux in AML cells. Specifically, LAPTM5 maintains lysosomal homeostasis by regulating lysosomal abundance and integrity: AraC-resistant (AraC-R) cells exhibit expanded lysosomal compartments and elevated expression of lysosomal membrane proteins LAMP1 and LAMP2, which are critical for autophagosome-lysosome fusion and lysosomal stability. Conversely, LAPTM5 depletion impairs autophagosome-lysosome fusion, reduces autophagic flux (as evidenced by accumulated p62 and decreased LC3-II/LC3-I ratios), and re-sensitizes resistant cells to AraC-induced apoptosis. These observations align with prior reports linking lysosomal function to drug resistance and suggest that LAPTM5 may protect AML cells from lysosome-mediated cell death, a hypothesis warranting further investigation into downstream apoptotic signaling cascades.

While our data establish autophagy as a central pathway through which LAPTM5 mediates resistance, the molecular links between LAPTM5, autophagy, and broader cellular processes (e.g., metabolism) remain incompletely defined. Emerging evidence highlights metabolic reprogramming as a key driver of AML chemoresistance: resistant blasts often exhibit elevated oxidative phosphorylation (OXPHOS) [47–49], and targeting OXPHOS or fatty acid metabolism enhances AraC efficacy [50]. Autophagy is tightly intertwined with metabolic homeostasis, providing substrates for energy production under stress [51]. Whether LAPTM5 coordinates autophagy with metabolic pathways (e.g., by regulating nutrient recycling to support OXPHOS) to sustain resistance is unknown and represents a critical future direction. Elucidating these connections could uncover combinatorial strategies (e.g., LAPTM5 inhibition + metabolic targeting) to maximize therapeutic efficacy.

The dual role of autophagy in promoting resistance in blasts whereas supporting homeostasis in normal hematopoietic stem cells (HSCs) complicates the clinical translation of global autophagy inhibitors in AML. For example, ATG7 deficiency in HSCs triggers malignant myelodysplasia [52], and autophagy defects upregulate NOTCH signaling to accelerate leukemogenesis [53], underscoring the need for context-specific autophagy targets. Our study positions LAPTM5 as a promising candidate: LAPTM5 is selectively upregulated in AraC-R AML cells, and its depletion impairs tumor growth and enhances AraC sensitivity in both subcutaneous and disseminated xenograft models without overt disruption of normal hematopoiesis (unpublished data). Building on our finding that Vpr-mediated degradation effectively restored chemosensitivity, future clinical translation could explore strategies that mimic this viral mechanism. For instance, developing therapeutic modalities that recapitulate Vpr’s mode of action—such as specific protein degraders (e.g., PROTACs) or Vpr-based gene therapies—represents a viable strategy to selectively eliminate chemoresistant blasts while sparing normal hematopoietic stem cells.

In summary, our findings identify LAPTM5 as a critical regulator of AraC resistance in AML, acting by maintaining lysosomal homeostasis and promoting late-stage autophagic flux. By linking LAPTM5 to autophagy-dependent survival under chemotherapeutic stress, we uncover a novel mechanism of treatment failure and highlight LAPTM5 as a precision target to overcome AraC resistance. Future studies will focus on defining LAPTM5’s interplay with metabolic pathways and developing LAPTM5-directed therapies to improve outcomes for refractory AML patients.

## Materials and methods

### Cell lines and cell culture

HEK-293T (human embryonic kidney cell line) and THP1 (human acute monocytic leukemia) cells were purchased from the American Type Culture Collection (ATCC). HL60 cells (human AML cell line) were purchased from Cellcook (Guangzhou, China). Cells were cultured in DMEM or RPMI 1640 (Gibco) supplemented with 10% fetal bovine serum (ExCell Bio). All cells were incubated at 37 °C in a 5% CO_2_ atmosphere.

### Reagents and antibodies

Cytarabine (AraC) was purchased from Selleck Chemicals (S1648). Anti-LAPTM5 (A17995), anti-LAMP1 (A21194), and anti-LAMP2 (A0593) antibodies were purchased from ABclonal. Anti-ACTIN (P30002), anti-SQSTM1/p62 (T55546), anti-CASP3 (TA7022), anti-PARP (T40050), and anti-LC3 (T55992) antibodies were purchased from Abmart. Lyso-Tracker Red (C1046) was purchased from Beyotime Biotechnology. Calcein-AM/PI reagent kit (CA1630) was purchased from Solarbio Life Sciences.

### scRNA-seq data processing and quality control

Raw scRNA-seq data were processed using the Seurat R package (v4.1.1). To ensure data quality, cells were filtered based on the following criteria: the number of detected genes (nFeature_RNA) was required to be between 200 and 5000, and the percentage of mitochondrial gene expression (percent.mt) was restricted to below 0.3. Following quality control, data normalization and the identification of highly variable genes were performed using the FindVariableFeatures function within Seurat.

### Establishment of AraC-resistant cell lines

AraC-resistant HL60 and THP1 cells (AraC-R) were generated by exposing the parental sensitive cells to gradually increasing concentrations of AraC. The induction process lasted for approximately 2 months. Initially, cells were cultured in medium containing 2 nM AraC. Once the cells recovered their growth rate and viability (>90%), the drug concentration was increased. This process was repeated until the cells could proliferate stably in the presence of 5 μM AraC. During this period, cellular drug sensitivity was monitored every two weeks using the CCK-8 assay. Stable resistance was confirmed when the IC_50_ value of the resistant cells was at least 10-fold higher than that of the parental cells. To maintain the resistant phenotype, AraC-R cells were continuously cultured with 5 μM AraC. For all experiments, cells were passaged in drug-free medium for 48 h prior to use to eliminate immediate drug effects.

### Plasmids and stable cell lines

The plasmids pLV3-U6-LAPTM5(human)-shRNA-GFP-Puro, pLV3-U6-GFP-Puro, and pLV3-CMV-LAPTM5(human)-3×FLAG-Blast were obtained from MiaoLing Bio, China. To obtain stable cell lines, the target plasmids were transfected into HEK-293T cells using PEI 25K (Aladdin, China) and the supernatant was harvested after 48 h and 72 h. HL60 cells were then infected with lentivirus and selected with puromycin (0.5 μg/ml) to obtain stable cell lines.

### Cell viability assay

Cell Counting Kit-8 (CCK-8, APExBIO Technology, K1018) and apoptosis detection kit (Elabscience Biotechnology, E-CK-A258) were applied to analyze HL60 cell viability. For the CCK8 assay, HL60 cells (8000 cells/well) were seeded in 96-well plates and treated with AraC for 48 h. Cell viability was subsequently determined using the CCK-8 assay. For apoptosis detection, HL60 cells were exposed to AraC for 48 h, followed by staining with APC Annexin-V and DAPI reagent in the dark for 15 min. Flow cytometry was utilized to quantitatively assess the apoptosis levels of HL60 cells.

### Western blotting

Cells were treated with PBS or indicated concentrations of AraC, then suspended with cell lysis buffer (Epizyme Biotech, PC101) containing protease inhibitor (Epizyme Biotech, GRF101). Protein concentration was quantified using the Bradford assay (Bio-Rad, 5000201). The protein samples were separated by 10–12% SDS–PAGE and transferred to PVDF membrane (Bio-Rad, 1620177). Then membranes were blocked with 5% skimmed milk and incubated with primary antibodies. Signals were detected using an imaging system (Baygene Biotech, BG-gdsAUTO 730) at room temperature.

### RNA isolation and quantitative reverse transcription PCR (RT-qPCR)

Total RNA was extracted by RNA extraction reagent (Vazyme, R401-01). cDNA was synthesized using cDNA Synthesis SuperMix (TransGen Biotech, AE341-02). RT-qPCR was performed using SYBR Green RT-qPCR SuperMix (TransGen Biotech, AQ101-01) on LightCycler 96 Instrument (Roche). Relative mRNA expression fold changes were calculated using 2^−ΔΔCt^ method. The sequences of the RT-qPCR primers were provided in the Supporting Information (Supplementary Table S1).

### Immunofluorescence assay (IF)

The cell suspension was affixed to a glass slide using a Cytocentrifuge. Following this, 4% paraformaldehyde was added, and the slide was fixed for 15 min at room temperature, then aspirated and washed three times with PBS for 5 min each time. To the slide, 0.5% Triton X-100 was added, incubated at room temperature for 10 min, washed three times with PBS, followed by blocking at room temperature for 45 min in the presence of 10% goat serum. The corresponding primary antibody was added and incubated overnight at 4 °C. After 3 washes with PBST solution, the fluorescent secondary antibody was added and incubated for 1 h at room temperature, protected from light. Finally, an anti-fade mounting medium was applied, and the slides were observed as soon as possible. Images were acquired using a Zeiss LSM 880 confocal microscope equipped with a 40× /1.3 Oil DIC objective lens. Image processing and analysis were performed using ZEN software (Carl Zeiss).

### Calcein/PI live/dead assay

Cells from different groups were seeded in 96-well plates with the same cell density and incubated with the same concentration of AraC. After drug treatment, cells were collected into centrifuge tubes, washed twice with PBS, and resuspended in Assay Buffer. An appropriate proportion of Calcein-AM and PI dyes was added according to the instructions and incubate at 37 °C in the dark for 20 min. After staining, the dye was removed by centrifugation, and the cells were resuspended with PBS, and observed as soon as possible. Images were captured using a Nikon fluorescence microscope equipped with a 10× objective lens.

### RNA sequencing and bioinformatics analysis

Total RNA was extracted from AraC-resistant AML cells (transfected with shNC or shLAPTM5) using Trizol reagent (Invitrogen, Carlsbad, CA, USA) according to the manufacturer’s protocol. RNA quality and integrity were assessed using an Agilent 2100 Bioanalyzer (Agilent Technologies, Palo Alto, CA, USA). mRNA was enriched using Oligo(dT) beads and fragmented into short fragments. cDNA libraries were constructed using the NEBNext Ultra RNA Library Prep Kit for Illumina (NEB, Ipswich, MA, USA) and sequenced on an Illumina NovaSeq 6000 platform by Gene Denovo Biotechnology Co. (Guangzhou, China).

For data analysis, raw reads were filtered using fastp (version 0.18.0) to obtain high-quality clean reads. Clean reads were mapped to the human reference genome using HISAT2. Gene expression levels were quantified using StringTie (version 1.3.1) and RSEM software. Differential expression analysis between groups was performed using DESeq2 software. Genes with a false discovery rate (FDR) < 0.05 and an absolute fold change ≥2 were considered differentially expressed genes (DEGs). To identify biological functions and pathways, GO and KEGG enrichment analyses were performed on the DEGs. Significance was determined using a hypergeometric test with FDR correction (FDR ≤ 0.05). Furthermore, GSEA was conducted to identify significant differences in gene sets between groups using GSEA software and the MSigDB database.

### Lysosome detection in living cells

Cells were collected into centrifuge tubes, centrifuged to remove supernatant, washed twice with PBS, and then suspended with culture medium. According to the instructions, Lyso-Tracker Red was added at the recommended concentration, incubated at 37 °C in the dark for 30 min, and after staining, the dye was removed by centrifugation. The cells were resuspended in the culture medium and observed using a confocal microscope. Live-cell imaging was performed using a Zeiss LSM 880 confocal microscope equipped with a 63 × /1.4 Oil DIC objective lens. Images were captured and analyzed using ZEN software.

### In vivo study

In the AML subcutaneous xenograft model, four-week-old male SCID mice were randomly divided into two groups. HL60 cells (1 × 10^6^ cells/mouse) were subcutaneously injected into the right flank of mice. Tumor growth and body weight were observed and recorded daily. Tumor volume was calculated using the formula: (Length × Width^2^)/2. For the AraC treatment group, mice were intravenously injected with AraC (12 mg/kg). All mice were euthanized after treatment. In all experiments, the volume of the subcutaneous tumor did not exceed 1000 mm^3^.

In the AML hematologic xenograft model, four-week-old male SCID mice were intraperitoneally injected with 200 μL of cyclophosphamide (CTX) (50 mg/kg) once daily for two days. HL60 cells (2 × 10⁶ cells/mouse) were then injected *via* the tail vein. On day 10, to evaluate chemosensitivity, mice in each group (AR-shNC and AR-shLAPTM5) were randomized into two subgroups. From day 11 to day 17, one subgroup received daily intravenous injections of AraC (12 mg/kg), while the other received vehicle control (PBS). The percentage of hCD45-positive cells in peripheral blood was measured by flow cytometry on days 1, 10 (pre-treatment), and 20 (post-treatment).

### Statistical analysis

All in vitro experiments were performed with three independent replicates. Data were analyzed using GraphPad Prism software (version 9). Comparisons between two groups were performed using the Student’s *t* test. For comparisons among more than two groups, one-way analysis of variance (ANOVA) followed by Dunnett’s multiple comparisons test was utilized. All data are presented as mean ± SD. *p* < 0.05 was considered statistically significant. NS: no significant difference, when *p* > 0.05; *: *p* < 0.05; **: *p* < 0.01; ***: *p* < 0.001.

### Ethics statement

All animal studies were conducted in accordance with institutional guidelines and were approved by the Institutional Animal Care and Use Committee at Sun Yat-Sen University (SYSU-IACUC-2023-001717).

## Supplementary information

SUPPLEMENTAL MATERIAL

Related Manuscript File WB Data

## Data Availability

The RNA sequencing data generated in this study have been deposited in the Genome Sequence Archive for Human (GSA-Human) under accession code HRA013201 (BioProject: PRJCA045916). Other data supporting the findings of this study are available from the corresponding author upon reasonable request.
