# Supplementary material for: Targeting LAPTM5 enhances AML sensitivity to cytarabine through autophagy inhibition
Source: Cell Death Dis. 2026 Mar 30;17(1):432. doi: 10.1038/s41419-026-08654-9 (PMC13158302; doi:10.1038/s41419-026-08654-9)
Supplement: Supplementary file 1 — SUPPLEMENTAL MATERIAL [file 41419_2026_8654_MOESM1_ESM.docx]

**Supplementary Information**

**Targeting LAPTM5 enhances AML sensitivity to cytarabine through autophagy inhibition**

Yuqing Zeng^1┲^, Chao He^1,2┲^, Hongbo Chen^1, *^, Fang Cheng^1, *^

1. School of Pharmaceutical Sciences (Shenzhen), Sun Yat-sen University, Shenzhen, 518107, P.R. China.
2. Department of Endocrinology, Southwest Hospital, Army Medical University (The Third Military Medical University), Chongqing 400038, China.

* Correspondence to: [chengf9@mail.sysu.edu.cn](mailto:chengf9@mail.sysu.edu.cn) (F. Cheng) #

[chenhb7@mail.sysu.edu.cn](mailto:chenhb7@mail.sysu.edu.cn) (H. Chen)

# Fang Cheng will handle correspondence at all stages of refereeing and publication, also post-publication.

[Tel: +86-15527709102](Tel:+86-15527709102);

^┲^ Contributed equally.

Competing Interests statement

The authors declare no competing financial interest.

**Table S1: Primers used for qPCR.**

| **Human Gene** | **Primer sequence** | |
| --- | --- | --- |
|  | **Forward (5′–3′)** | **Reverse (5′–3′)** |
| ACTB | CATGTACGTTGCTATCCAGGC | CTCCTTAATGTCACGCACGAT |
| LAPTM5 | GCGTCTTGTTGTTCATCGAGC | CGATCCTGAGGTAGCCCAT |
| LAMP1 | TCTCAGTGAACTACGACACCA | AGTGTATGTCCTCTTCCAAAAGC |
| LAMP2 | GCACAGTGAGCACAAATGAGT | CAGTGGTGTGTATGGTGGGT |

**Supplementary Figures**


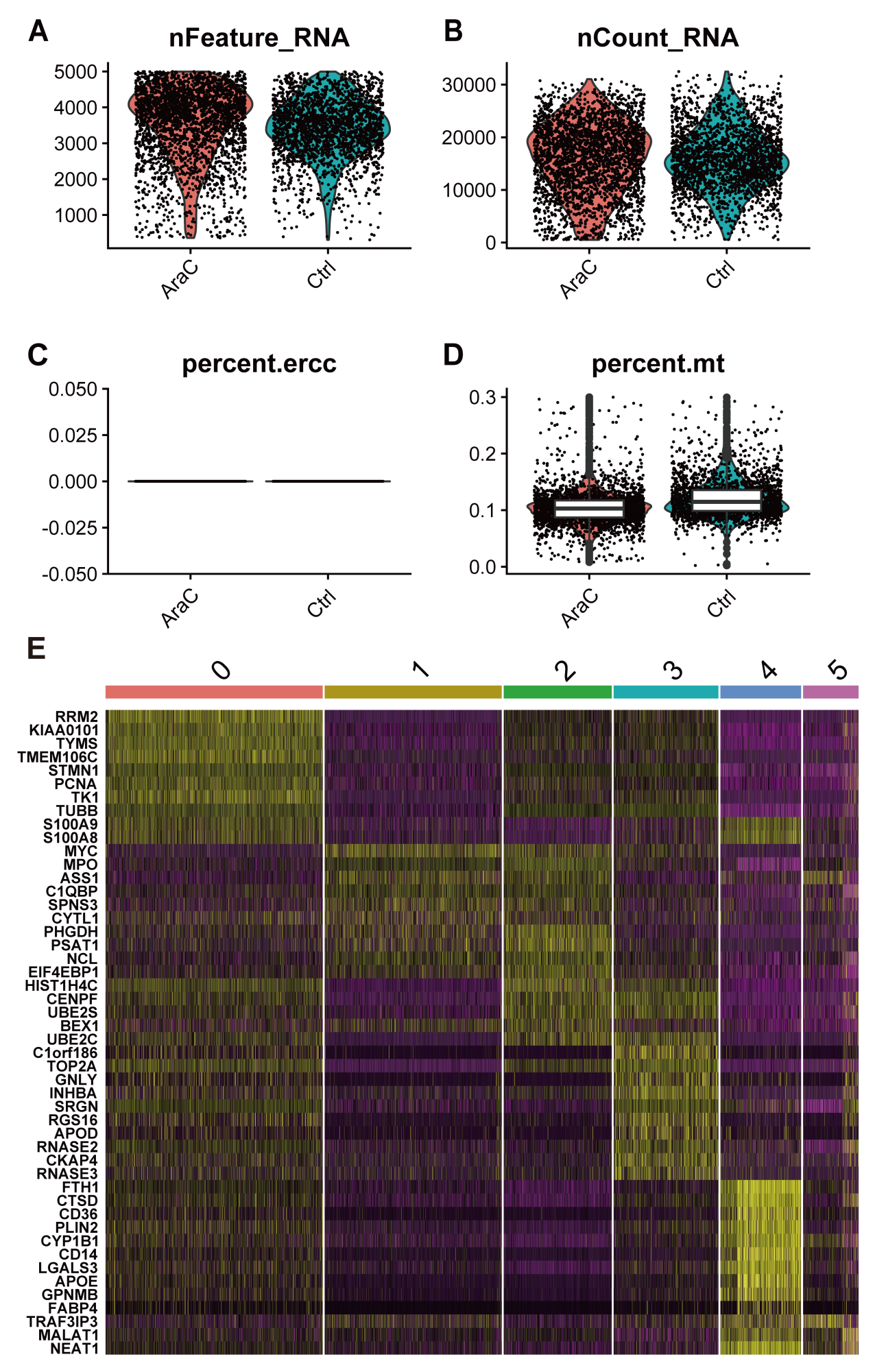


**Fig. S1. Quality control metrics and cluster markers for scRNA-seq analysis.**

(**A-B**) Violin plots showing the distribution of the number of expressed genes (nFeature_RNA) (**A**) and unique molecular identifier (UMI) counts (nCount_RNA) (**B**) in AraC-treated and control AML cells. (**C**) Distribution of External RNA Controls Consortium (ERCC) spike-in percentages in the two groups. (**D**) Distribution of mitochondrial gene content percentages (percent.mt). (**E**) Heatmap displaying the expression levels of the top 10 marker genes for each of the 6 identified cell clusters (0-5).

**
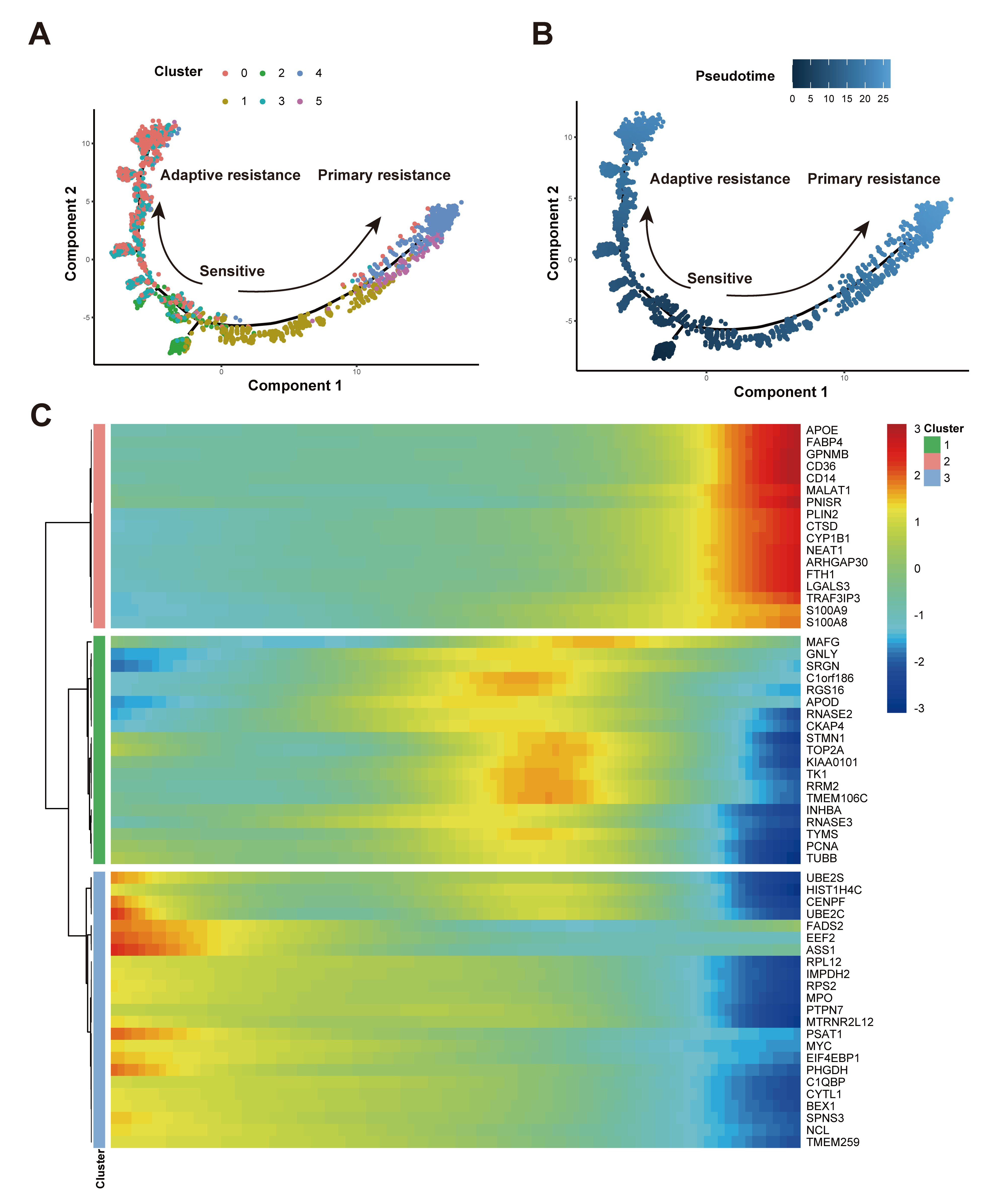
**

**Fig. S2. Pseudotime trajectory analysis of AraC resistance.**

(**A**) Monocle pseudotime trajectory of AML cells colored by cell cluster. (**B**) Pseudotime trajectory colored by pseudotime values (dark to light blue). (**C**) Heatmap of gene expression dynamics along the pseudotime axis, illustrating distinct expression patterns corresponding to the three cell populations: Sensitive (green bar), Adaptive Resistance (pink bar), and Primary Resistance (blue bar).

**
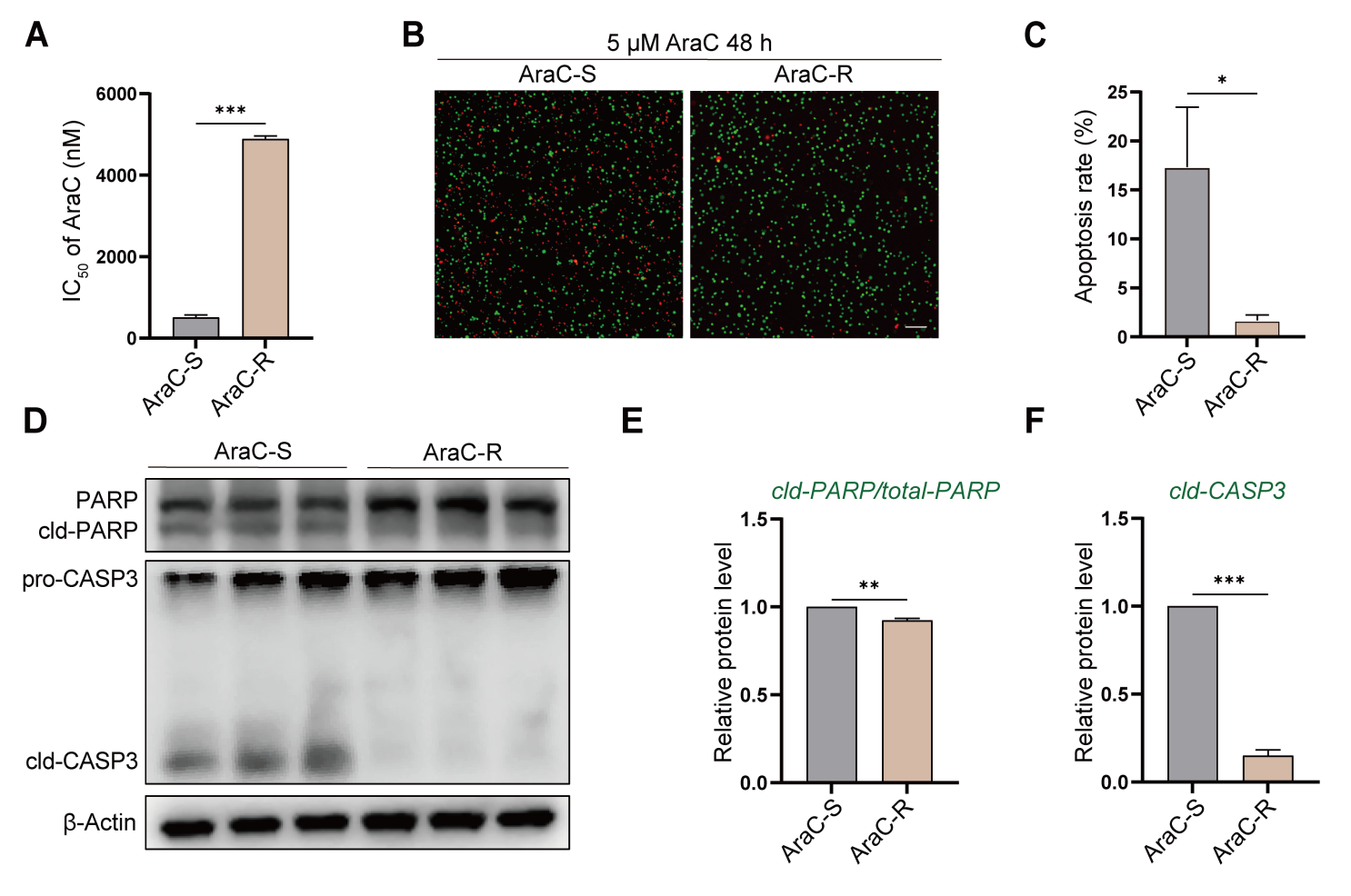
**

**Fig. S3. Characterization of the AraC-resistant HL60 cell line.**

(**A**) IC_50_ determination of AraC in HL60 AraC-sensitive (AraC-S) and resistant (AraC-R) cells using CCK-8 assay (48 h treatment). *n =* 6. (**B-C**) Representative Live/Dead staining images (Calcein-AM/PI) (**B**) and quantification of the apoptosis rate (**C**) in HL60 AraC-S and AraC-R cells treated with 5 μM AraC for 48 h. Green fluorescence indicates live cells; red fluorescence indicates apoptotic/dead cells. The apoptosis rate was calculated as: red fluorescent cell counts / total cell counts. *n =* 3. Scale bars: 200 μm. (**D-F**) Western blot analysis (**D**) and quantification (**E, F**) of PARP and Caspase-3 cleavage in HL60 AraC-S and AraC-R cells treated with 5 μM AraC for 48 h. *n =* 3. **Abbreviations**: cld, cleaved; pro, pro-form.

**
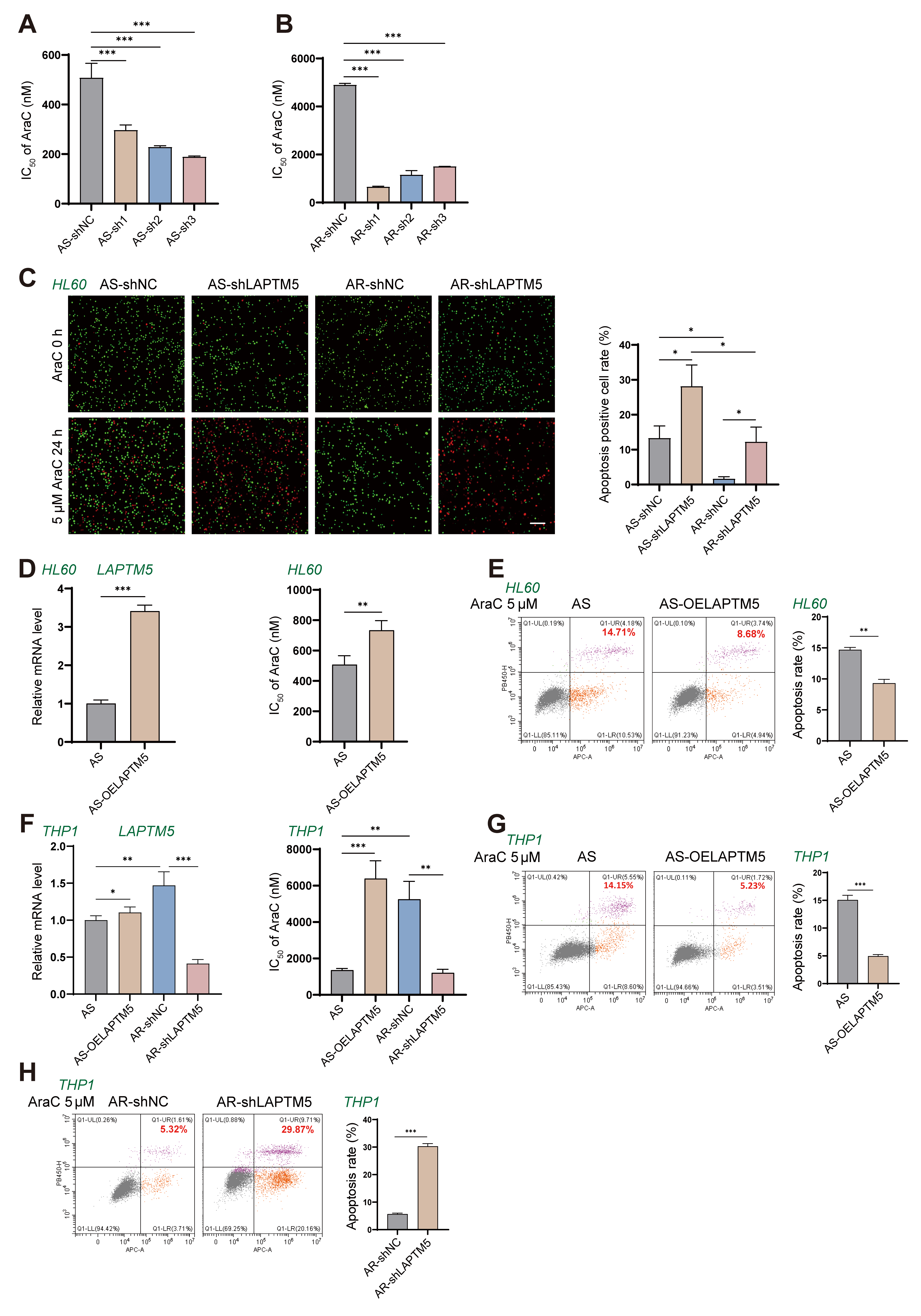
**

**Fig. S4. Validation of LAPTM5-mediated drug sensitivity in HL60 and THP1 cells.**

(**A-B**) IC_50_ values of AraC in HL60 AraC-S (A) and AraC-R (B) cells transfected with scramble control (shNC) or LAPTM5-targeting shRNAs (sh1, sh2, sh3) (CCK-8 assay, 48 h). *n =* 6. (**C**) Representative Live/Dead staining images (Calcein-AM/PI) and quantification of apoptosis in HL60 AraC-S and AraC-R cells transduced with shNC or shLAPTM5 and treated with 5 μM AraC for 48 h. *n =* 3. Scale bars: 200 μm. (**D**) *LAPTM5* mRNA levels (left) and AraC IC_50_ values (right) in HL60 AraC-S cells overexpressing LAPTM5 (AS-OE). *n =* 3. (**E**) Flow cytometric quantification of apoptosis in HL60 AS-OE cells treated with 5 μM AraC for 48 h. *n =* 3. (**F**) *LAPTM5* mRNA levels and AraC IC_50_ values in THP1 AraC-S cells overexpressing LAPTM5 (AS-OELAPTM5) and THP1 AraC-R cells with LAPTM5 knockdown (AR-shLAPTM5), compared to their respective controls (AS and AR-shNC). *n =* 3. (**G-H**) Flow cytometric quantification of apoptosis in THP1 AraC-S (OE) (**G**) and THP1 AraC-R (shLAPTM5) (**H**) cells treated with 5 μM AraC for 48 h. *n =* 3. **Abbreviations**: AS, AraC-sensitive; AR, AraC-resistant; OE, overexpression.

**
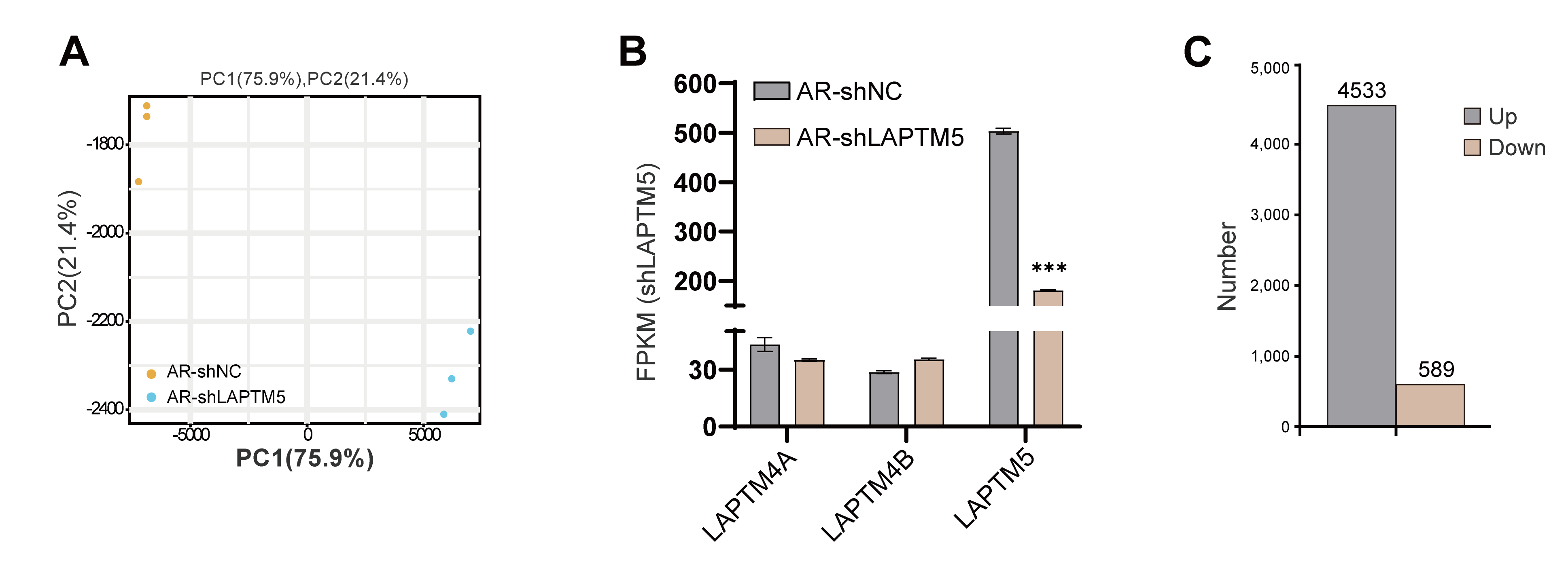
**

**Fig. S5. Transcriptional profiling of *LAPTM5*-knockdown cells.**

(**A**) Principal Component Analysis (PCA) of gene expression profiles showing distinct gene expression profiles between AraC-R shNC and AraC-R shLAPTM5 cells. *n* = 3. (**B**) FPKM values of LAPTM5 and its homologs (LAPTM4A, LAPTM4B) derived from RNA-seq data. (**C**) Bar chart showing the number of significantly upregulated and downregulated genes in *LAPTM5*-knockdown cells compared to control. **Abbreviations**: FPKM, fragments per kilobase of transcript per million mapped reads.


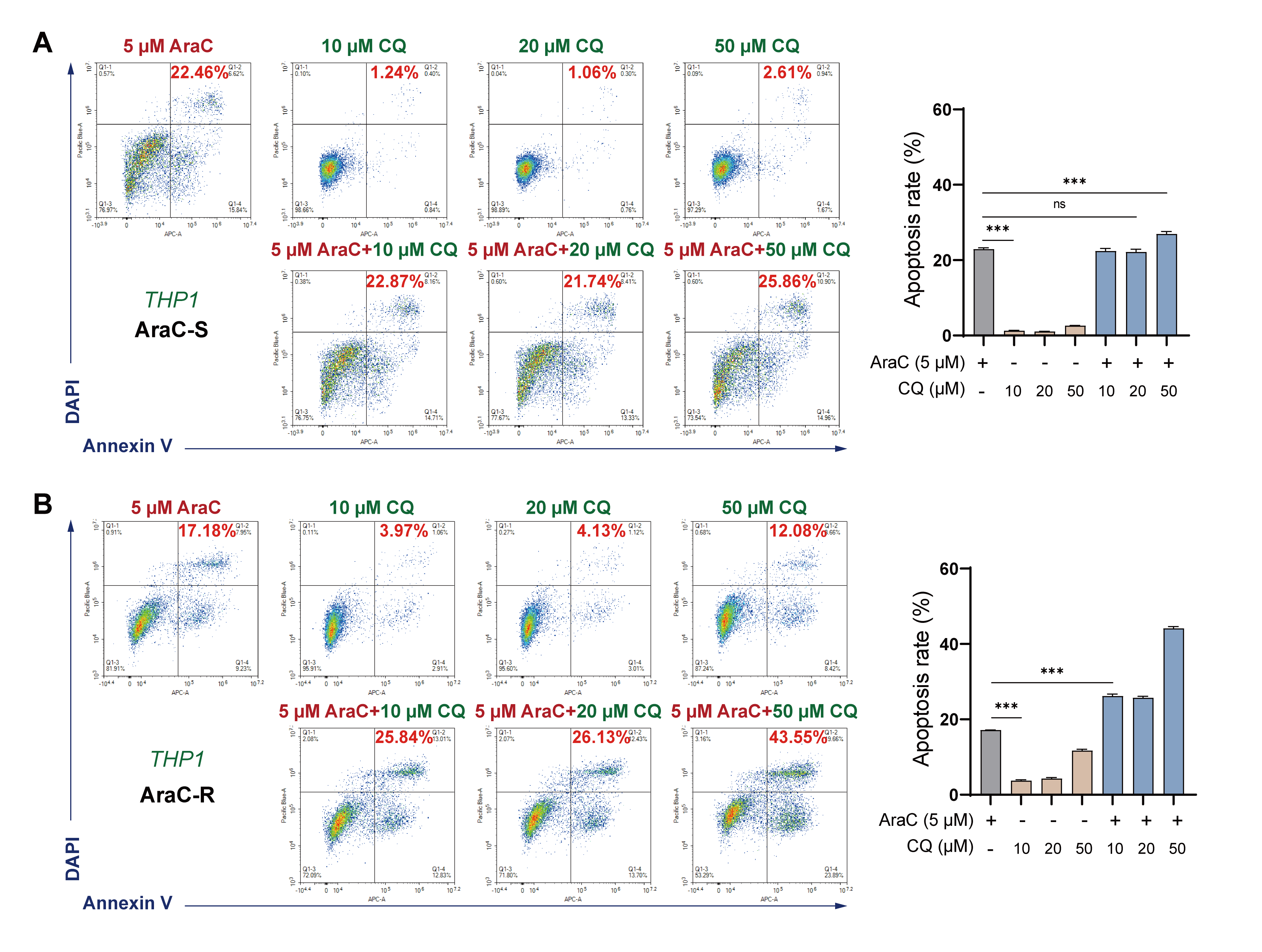


**Fig. S6. Validation of autophagy inhibition in THP1 models.**

(**A, B**) Flow cytometric analysis and quantification of apoptosis in THP1 AraC-S (**A**) and THP1 AraC-R (**B**) cells treated with 5 μM AraC alone or in combination with Chloroquine (CQ) at indicated concentrations (10, 20, 50 μM) for 48 h. *n* = 3.

**
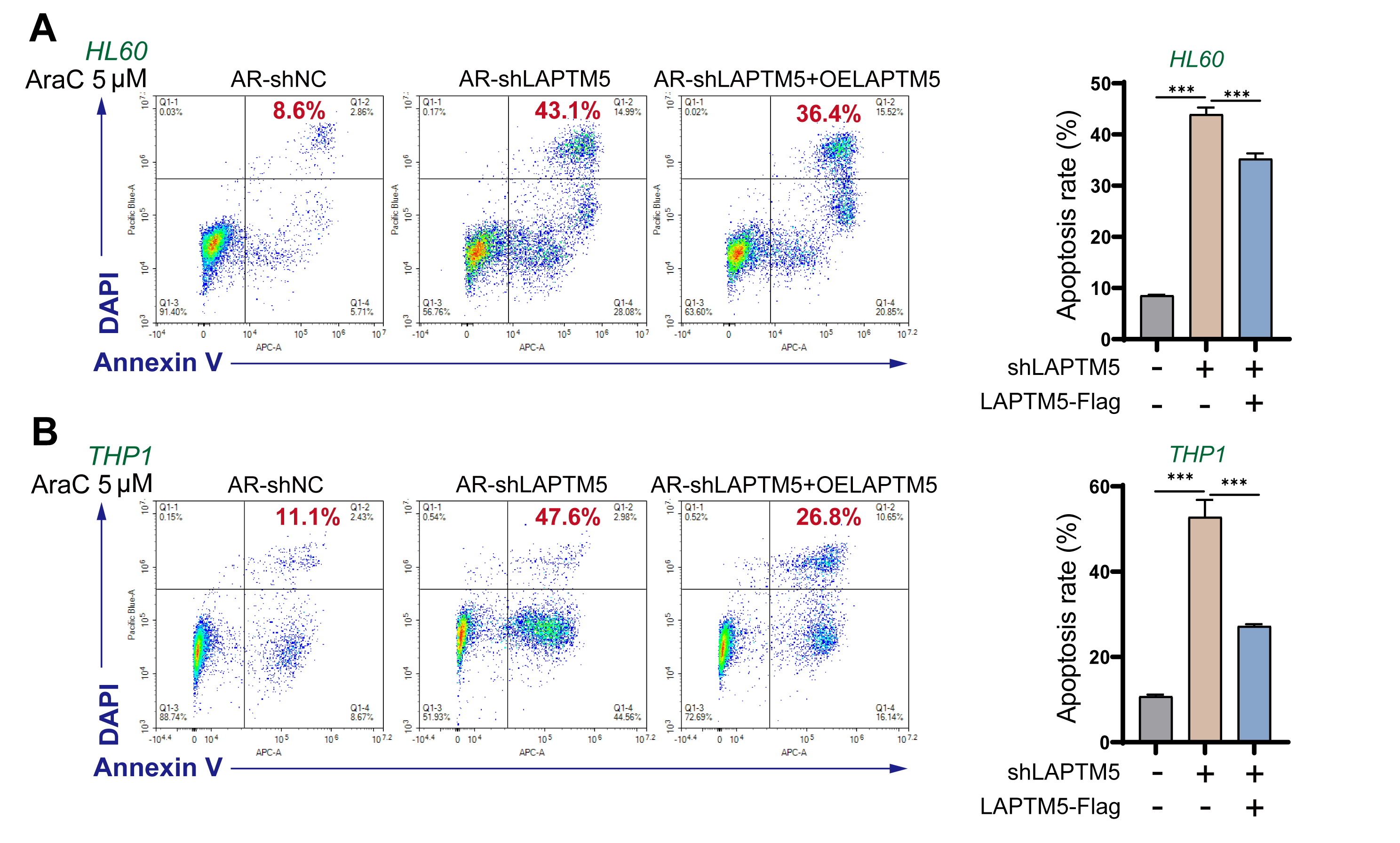
**

**Fig. S7. LAPTM5 re-expression rescues AraC resistance in knockdown cells.**

(A, B) Flow cytometric analysis and quantification of apoptosis in HL60 (A) and THP1 (B) AraC-resistant (AR) cells. Cells were transduced with control shRNA (shNC), LAPTM5-targeting shRNA (shLAPTM5), or co-transduced with shLAPTM5 and a Flag-tagged LAPTM5 overexpression vector (shLAPTM5+OELAPTM5), followed by treatment with 5 μM AraC for 48 h. Representative flow cytometry plots (left) and statistical quantification of apoptosis rates (right) are shown. *n* = 3.


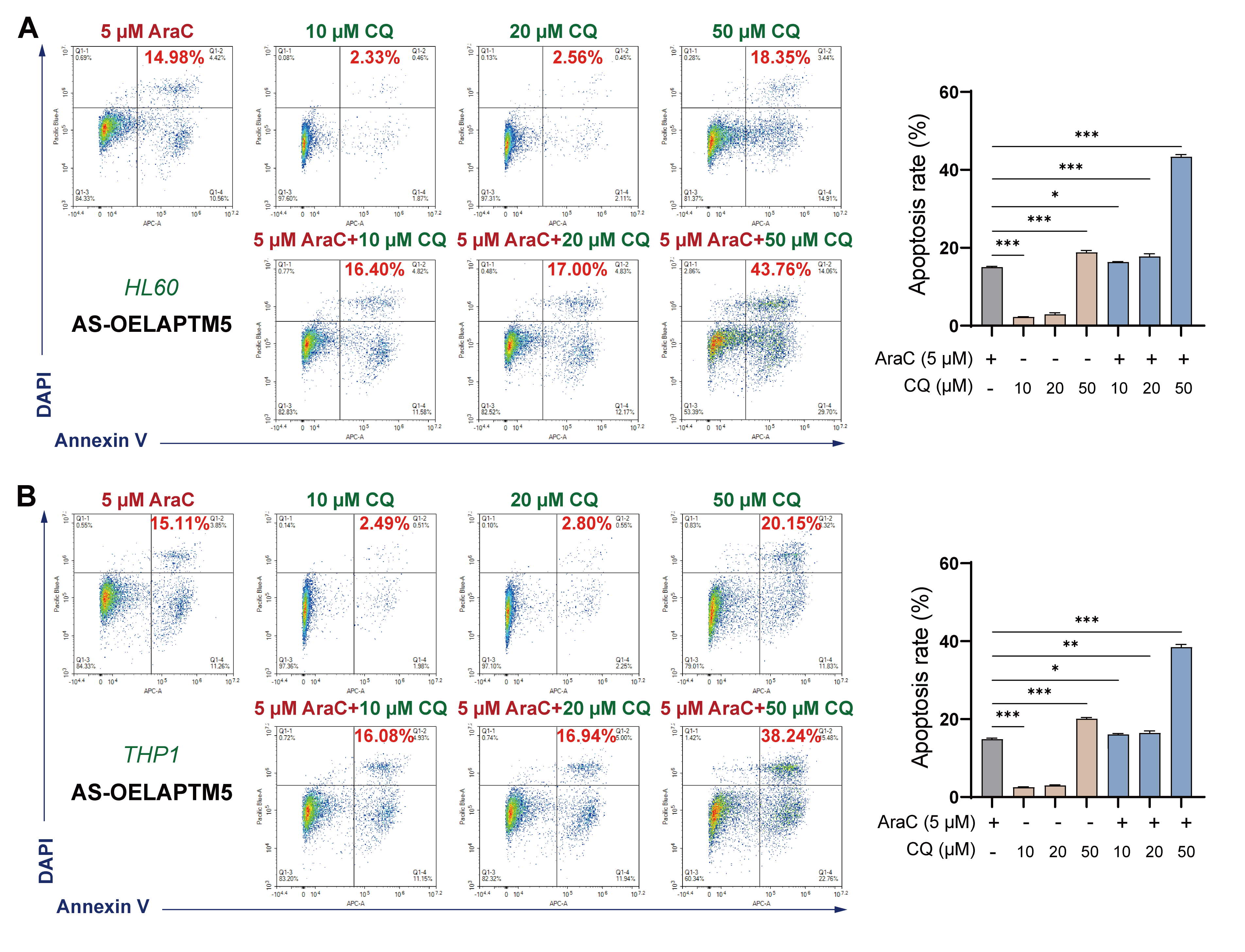


**Fig. S8. Validation of autophagy inhibition in LAPTM5-overexpressing AML cells.**

(**A, B**) Flow cytometric analysis and quantification of apoptosis in LAPTM5-overexpressing HL60 (**A**) and THP1 (**B**) cells (AS-OELAPTM5) treated with 5 μM AraC alone or in combination with Chloroquine (CQ) at indicated concentrations (10, 20, 50 μM) for 48 h. *n* = 3.

**
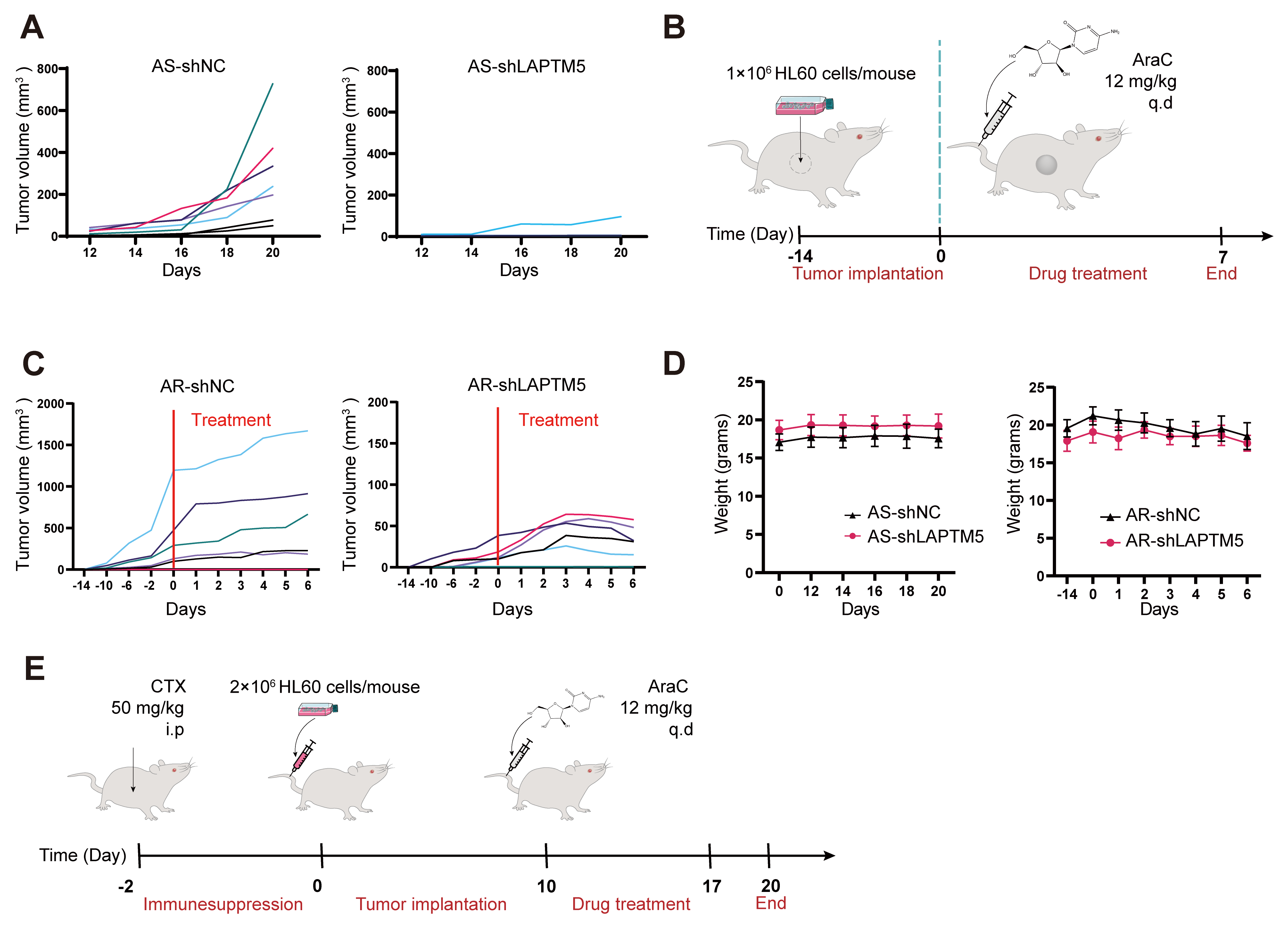
**

**Fig. S9. In vivo tumor metrics and experimental schedules.**

(**A**) Individual tumor growth curves (volume) for SCID mice in the subcutaneous AraC-sensitive model (AS-shNC vs. AS-shLAPTM5). (**B**) Schematic diagram of the experimental design and AraC dosing regimen for the subcutaneous AraC-resistant (AraC-R) xenograft model. (**C**) Individual tumor growth curves for SCID mice in the AraC-R model (AR-shNC vs. AR-shLAPTM5). The red vertical line indicates the start of AraC treatment. (**D**) Body weight monitoring of tumor-bearing mice throughout the experiment. (**E**) Schematic diagram of the hematologic AML xenograft model (tail vein injection), including immunosuppression (CTX), tumor implantation, and AraC treatment schedule. **Abbreviations**: CTX, cyclophosphamide; i.p, intraperitoneal injection; q.d, once daily.
