# Supplementary material for: Targeting LAPTM5 enhances AML sensitivity to cytarabine through autophagy inhibition
Source: Cell Death Dis. 2026 Mar 30;17(1):432. doi: 10.1038/s41419-026-08654-9 (PMC13158302; doi:10.1038/s41419-026-08654-9)
Supplement: Supplementary file 2 — Related Manuscript File WB Data [file 41419_2026_8654_MOESM2_ESM.docx]

**Fig 1J**

**LAPTM5**

**
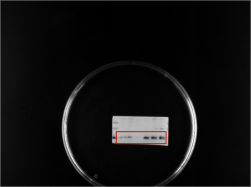
**

**β-Actin**

**
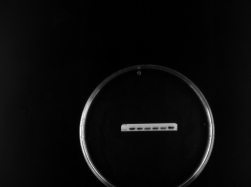
**

**Fig 2B**

**PARP**

**
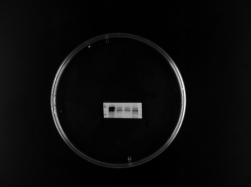
**

**Pro-CASP3/Cld-CASP3**

**
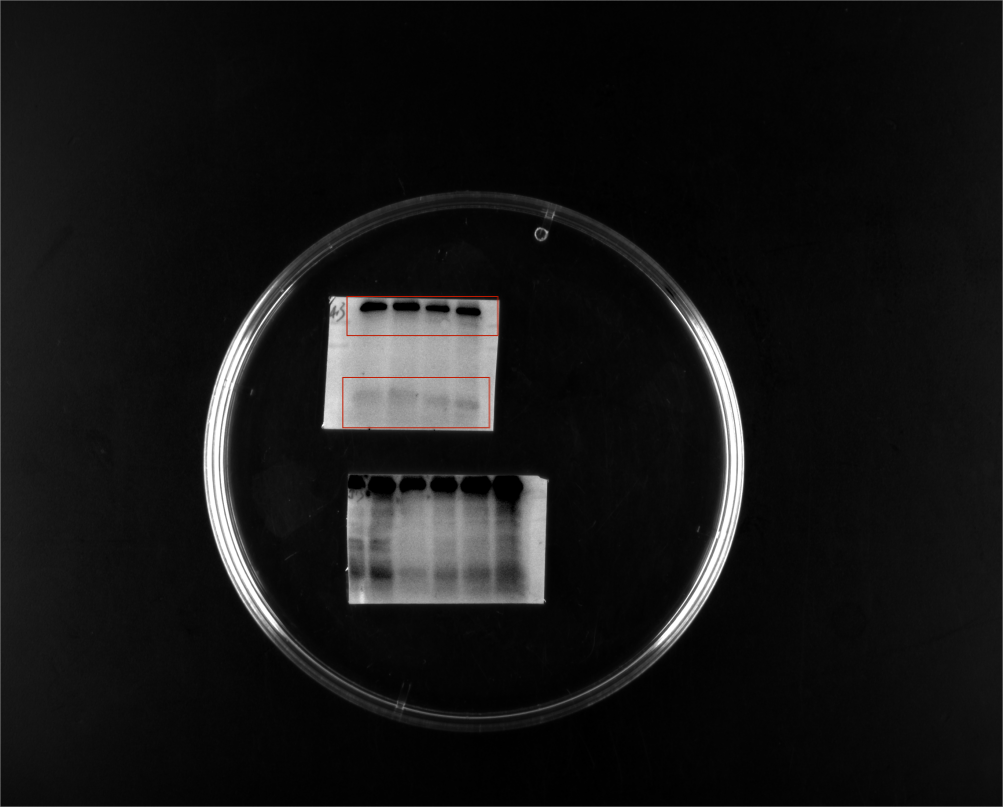
**

**LAPTM5**

**
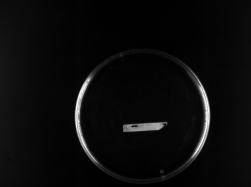
**

**β-Actin**

**
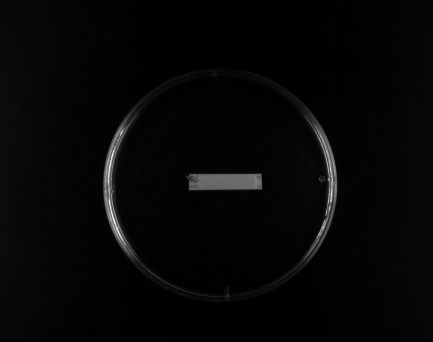

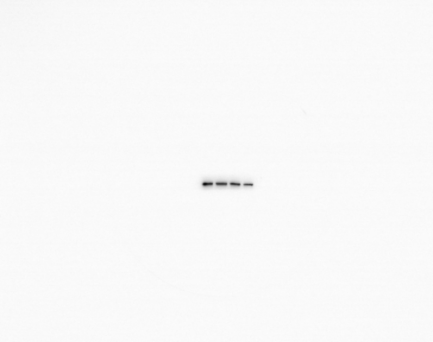
**

**Fig 2H**

**PARP**

**
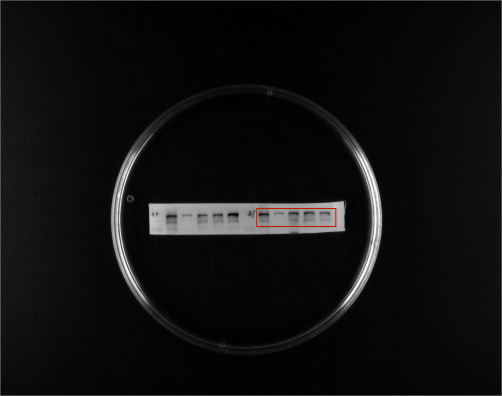
**

**Pro-CASP3/Cld-CASP3**

**
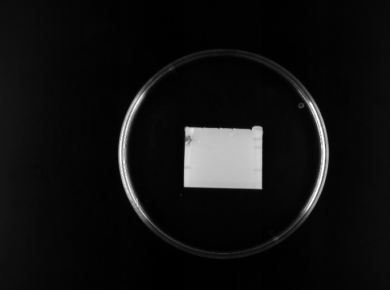

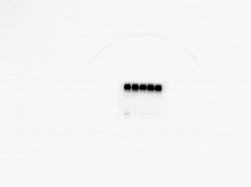

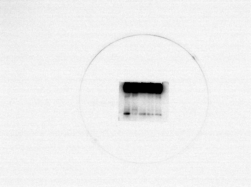
**

**LAPTM5**

**
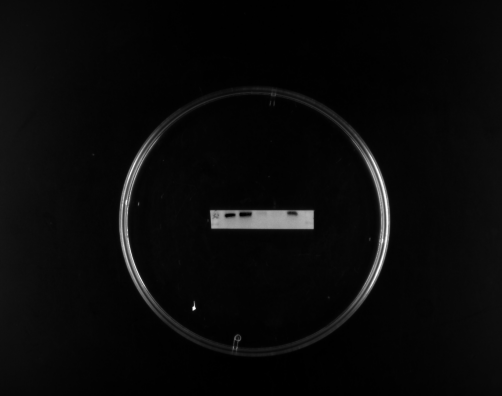
**

**β-Actin**

**
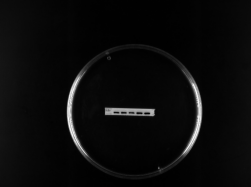
**

**Fig 3C**

**p62**

**
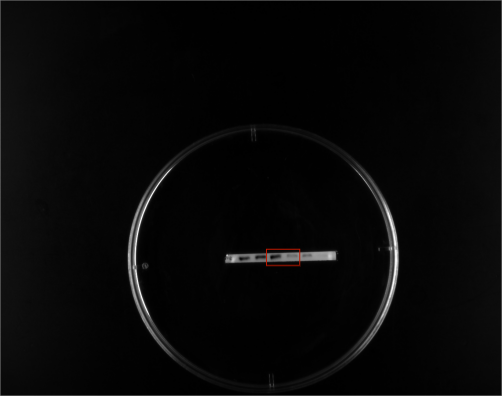
**

**LC3I/LC3II**

**
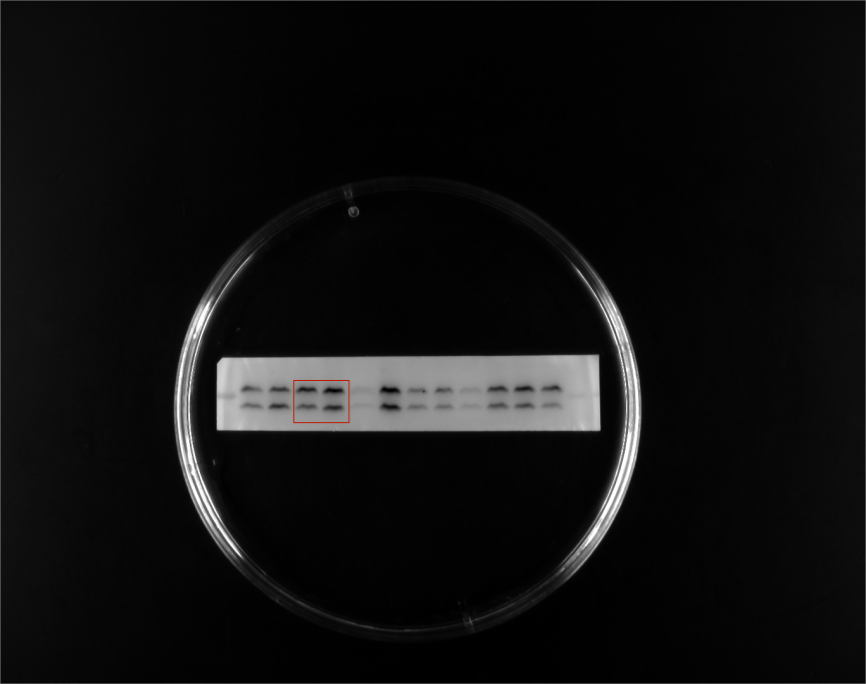
**

**β-Actin**

**
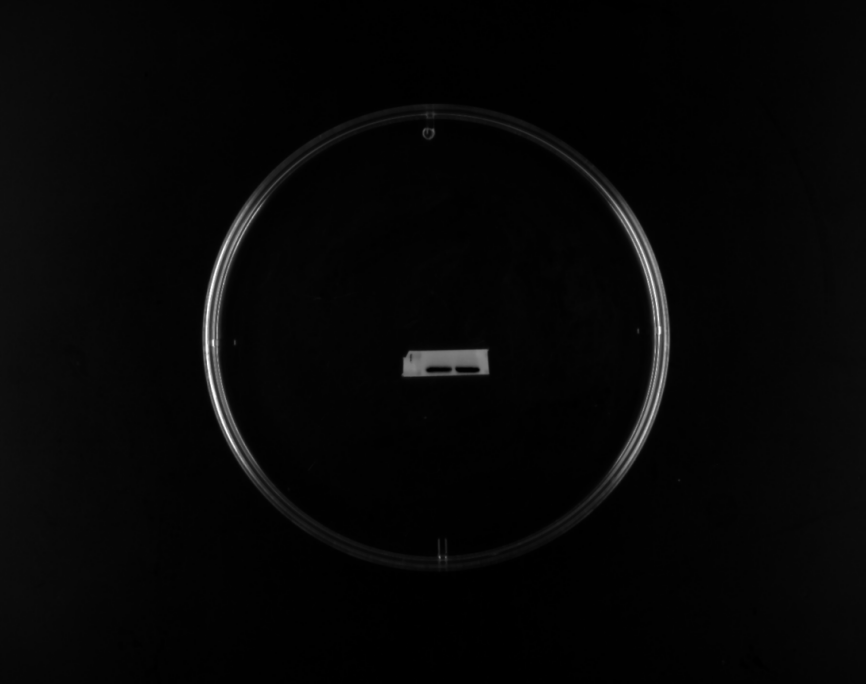
**

**Fig 4C**

**p62**

**
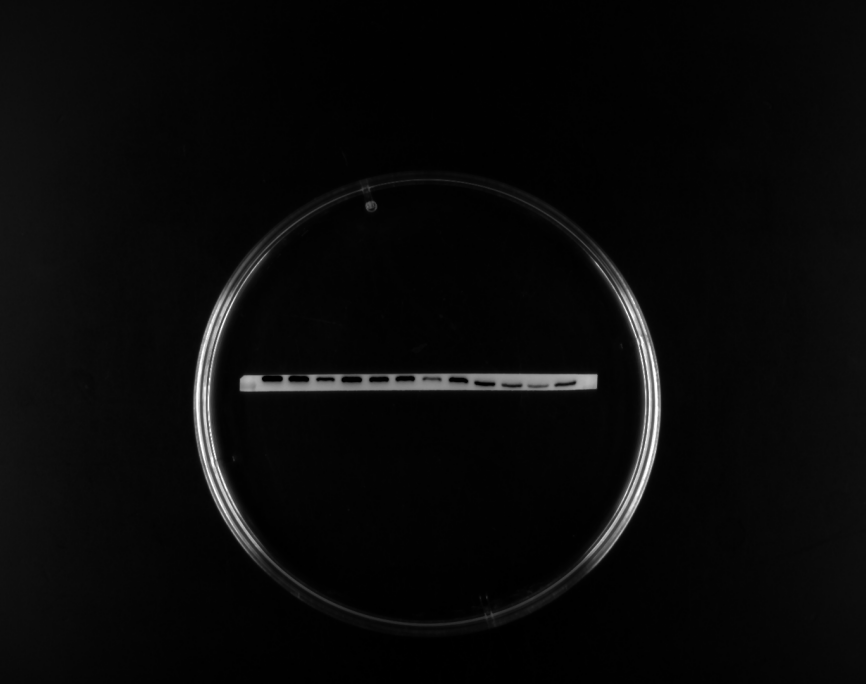
**

**LC3I/LC3II**

**
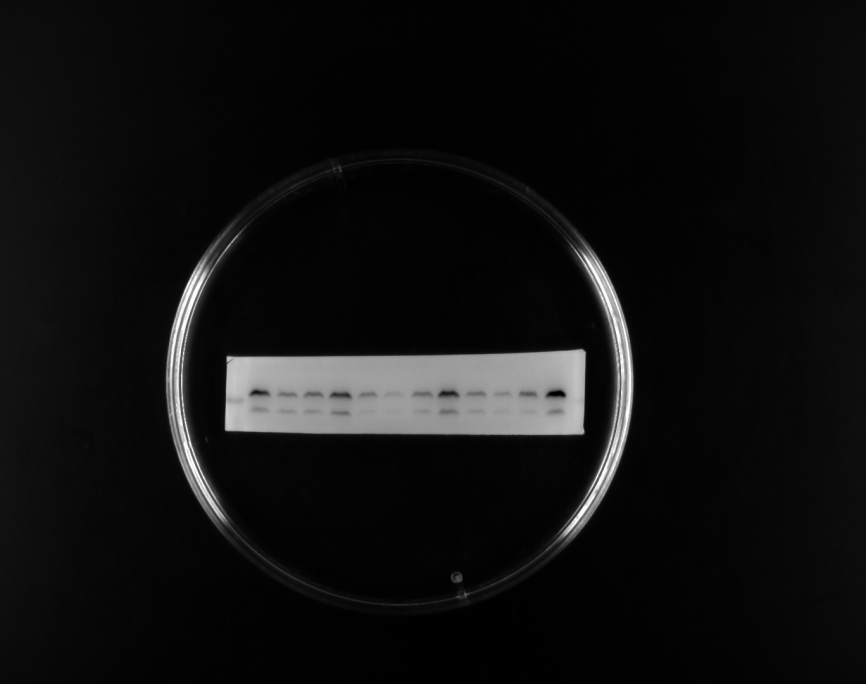
**

**LAPTM5**

**
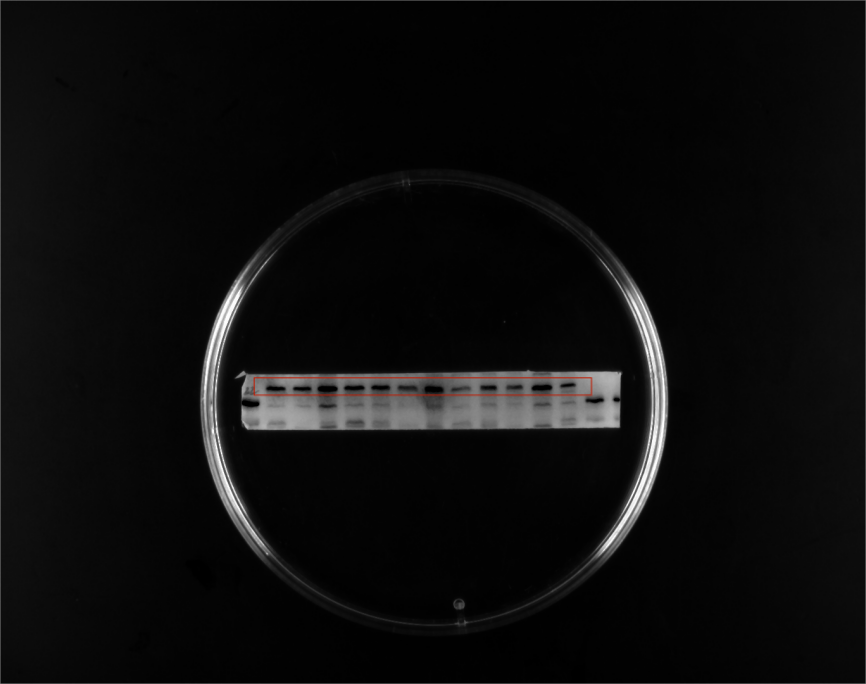
**

**β-Actin**

**
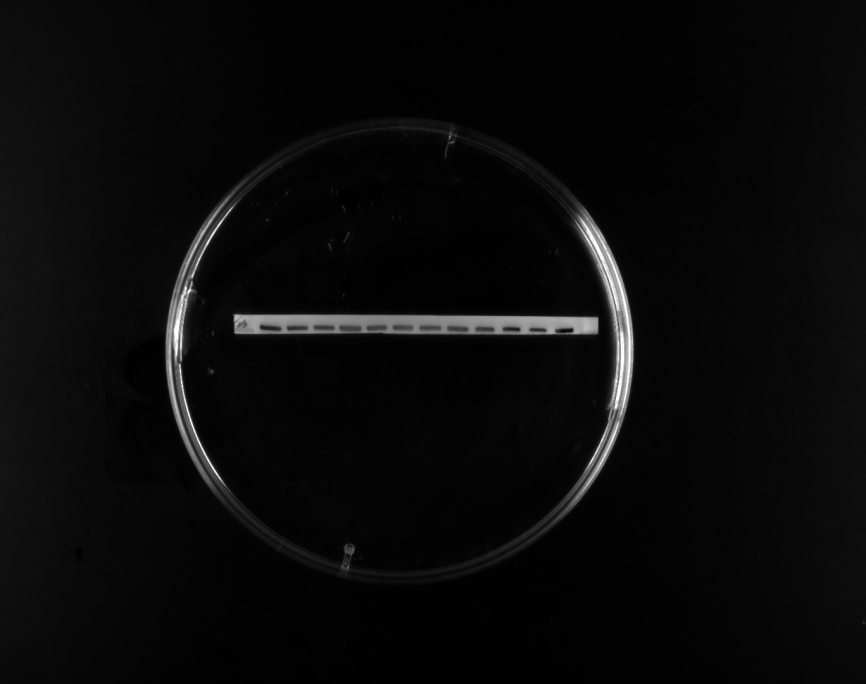
**

**Fig 4G**

**p62**

**
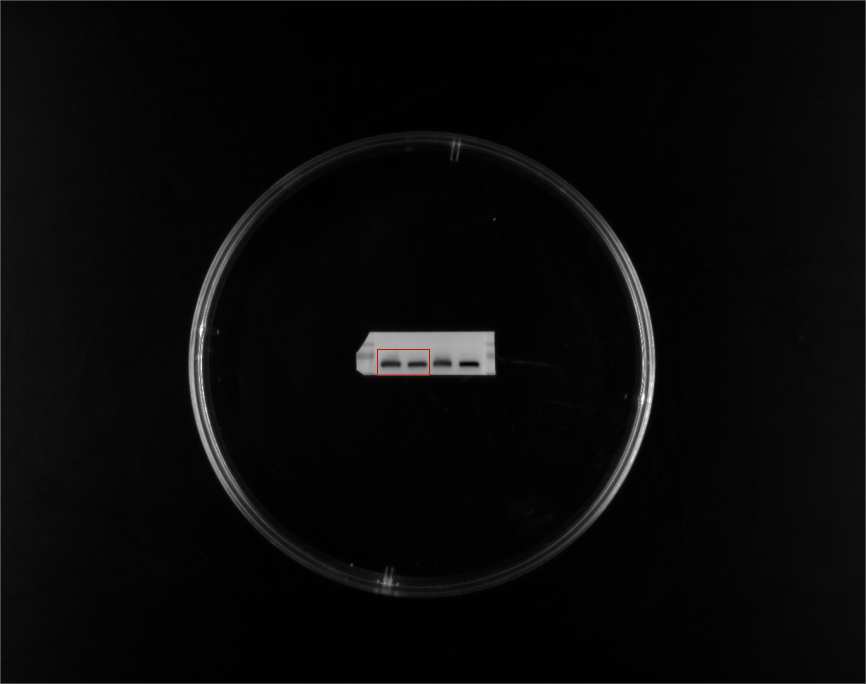
**

**LC3I/LC3II**

**
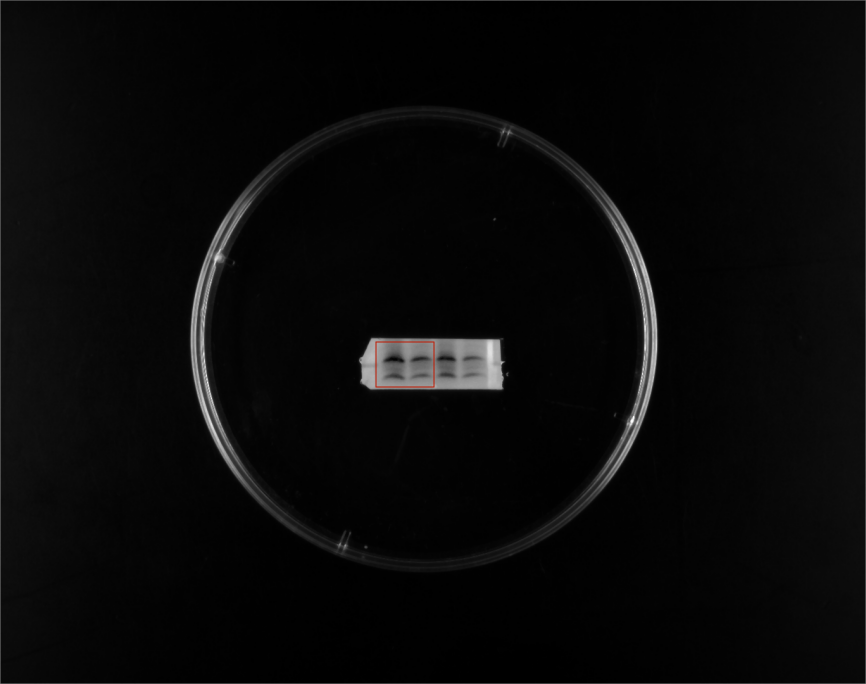
**

**LAPTM5**

**
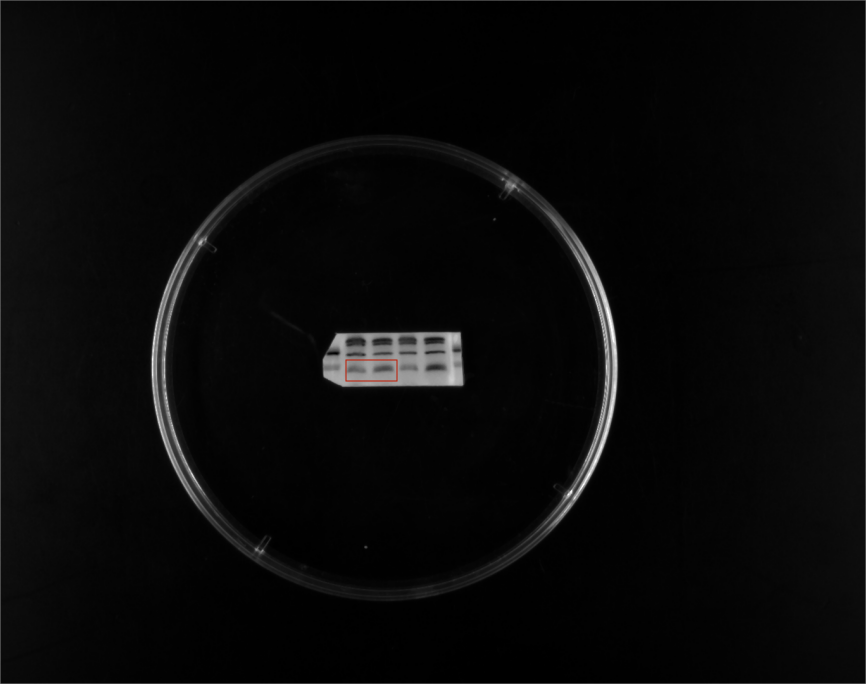
**

**β-Actin**

**
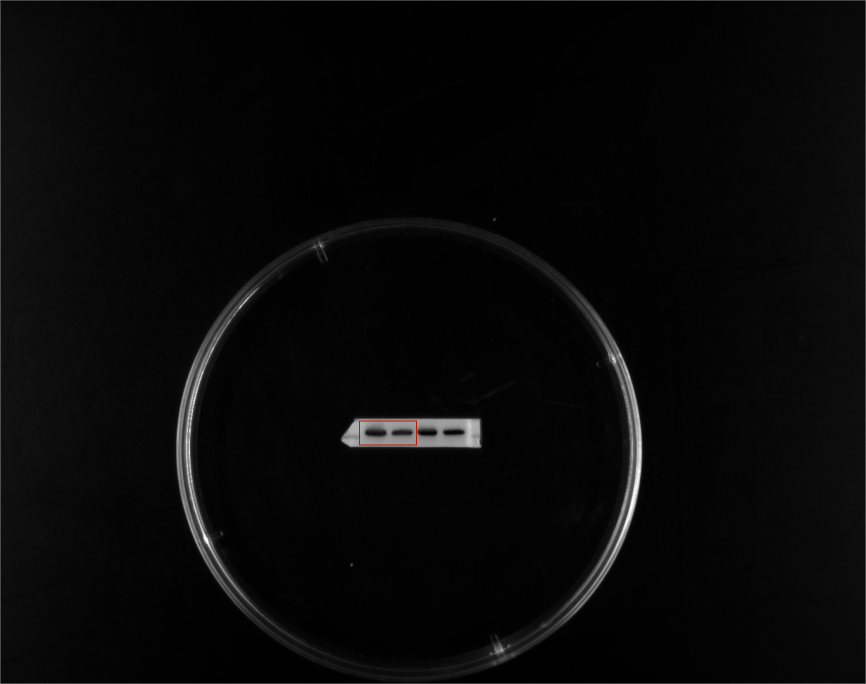
**

**Fig 4H**

**p62**

**
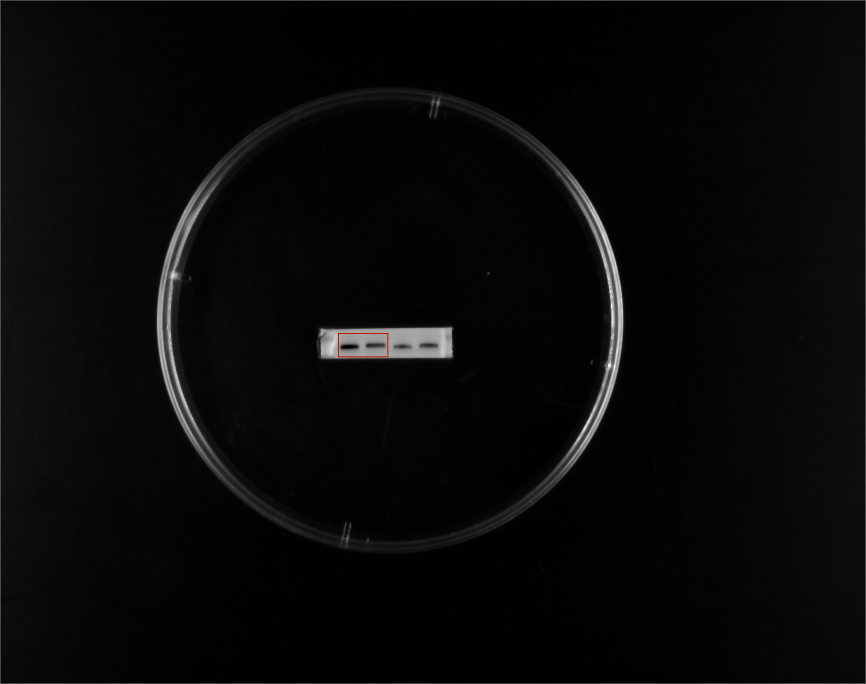
**

**LC3I/LC3II**

**
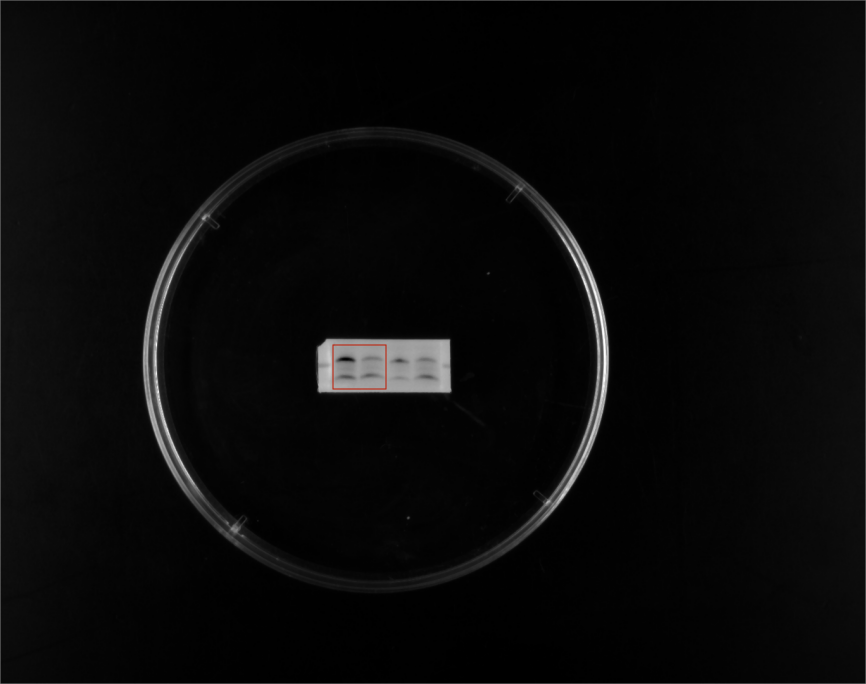
**

**LAPTM5**

**
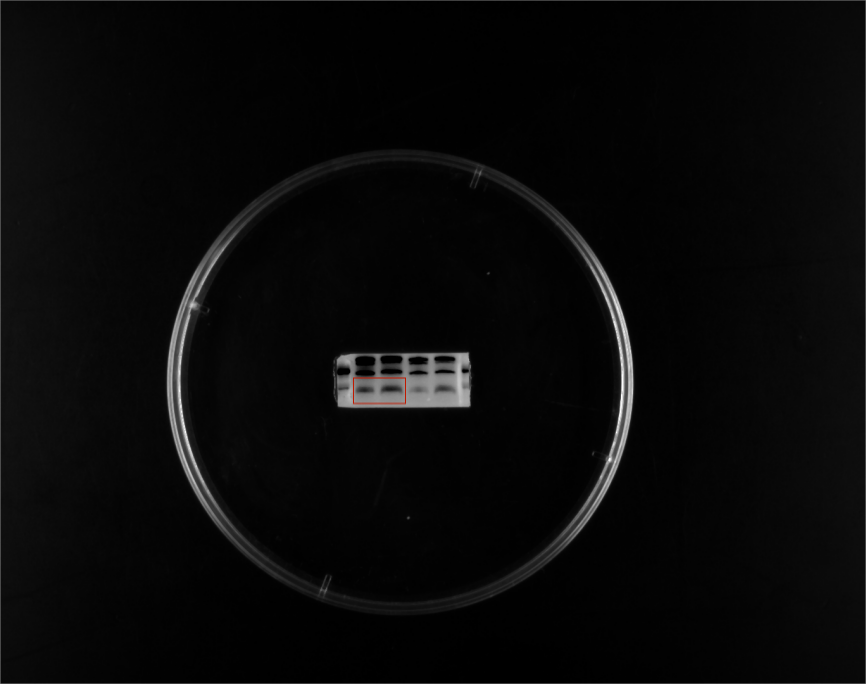
**

**β-Actin**

**
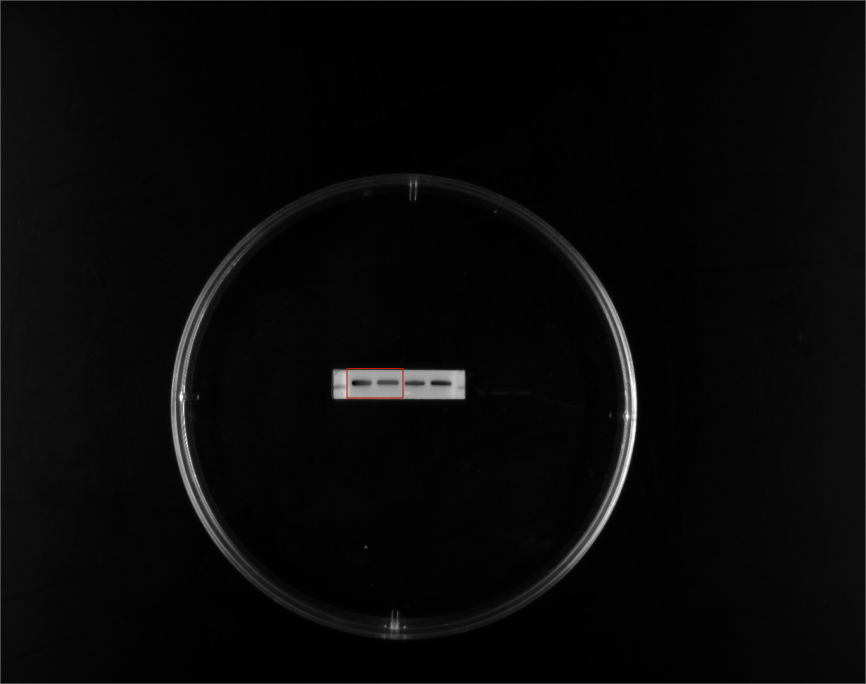
**

**Fig 4I**

**p62**

**
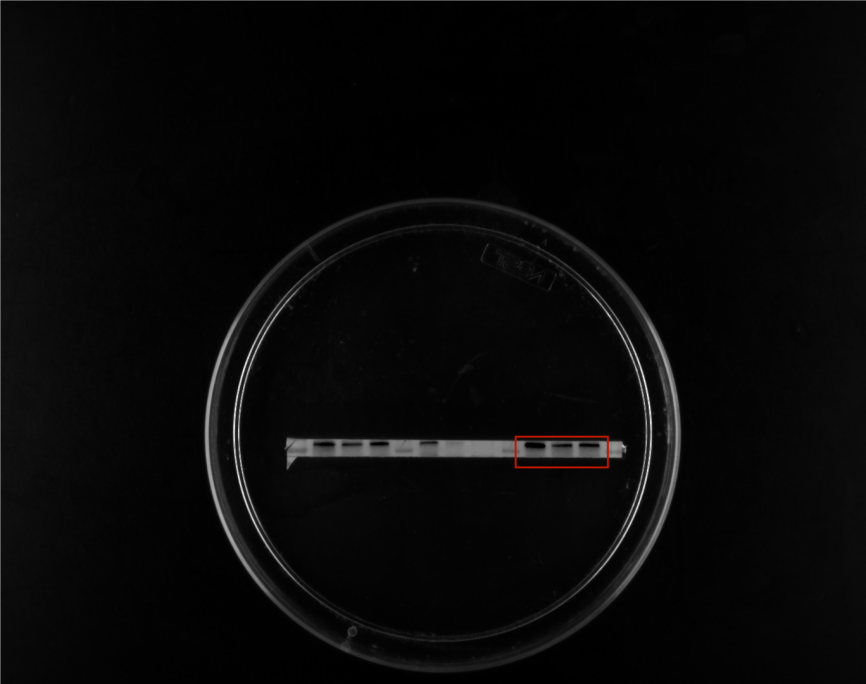
**

**β-Actin for p62**

**
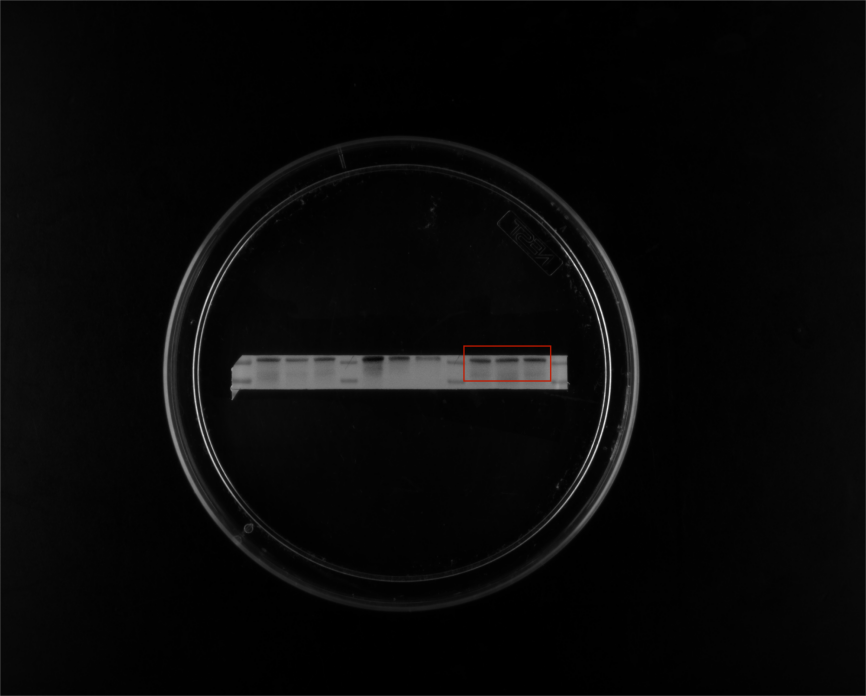
**

**LC3I/LC3II**

**
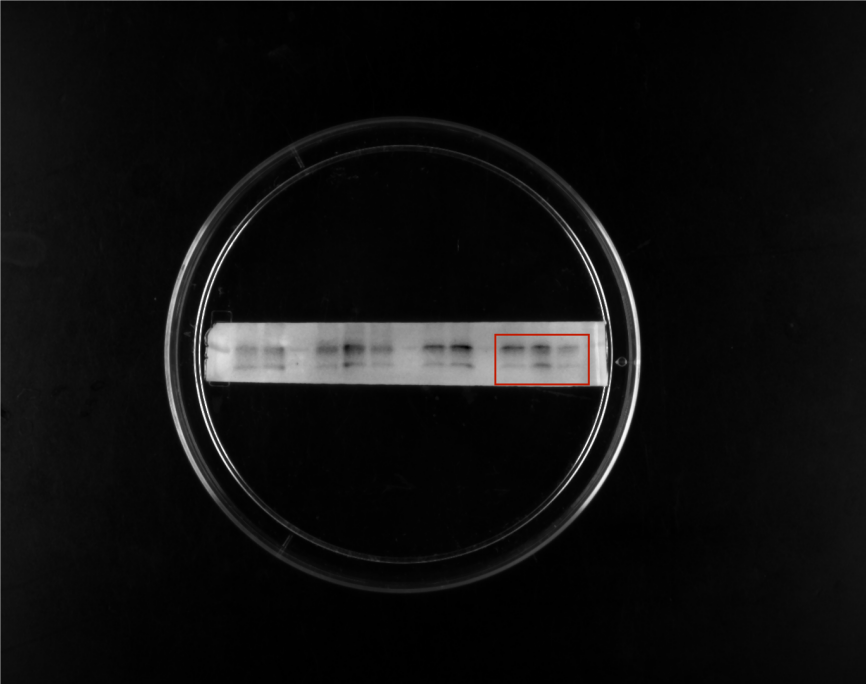
**

**β-Actin for LC3I/LC3II**

**
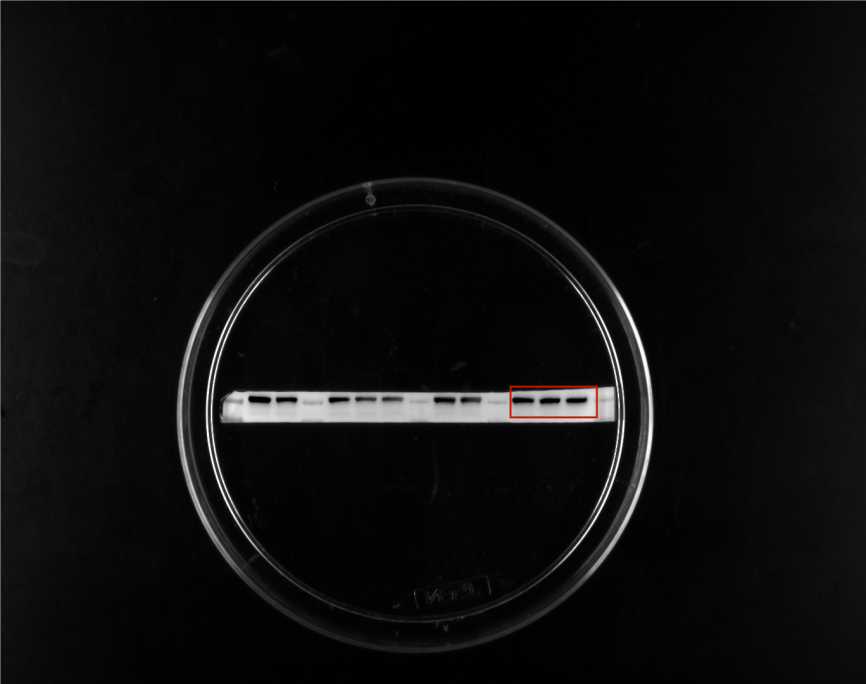
**

**LAPTM5**

**
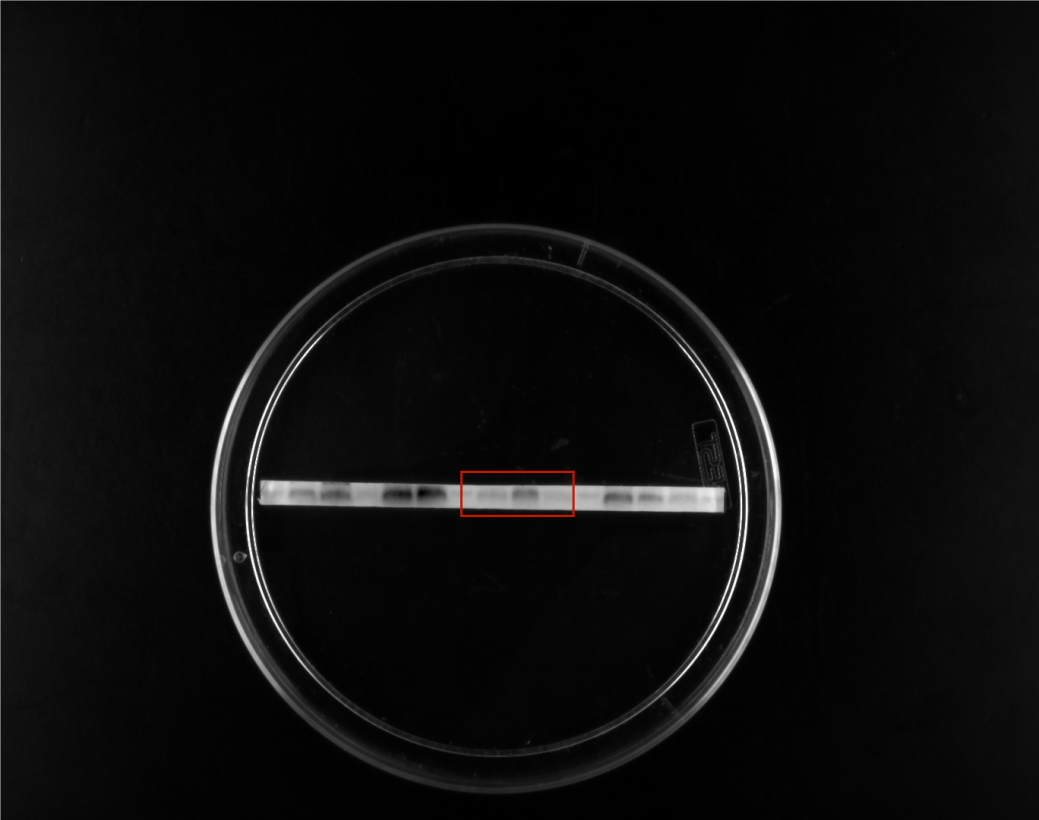
**

**β-Actin for LAPTM5**

**
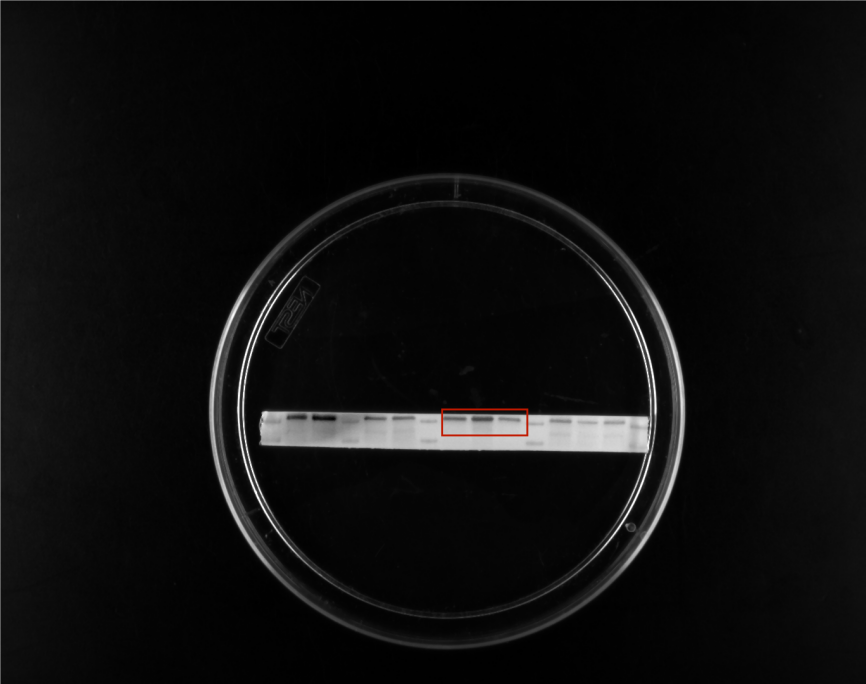
**

**Fig 4J**

**p62**

**
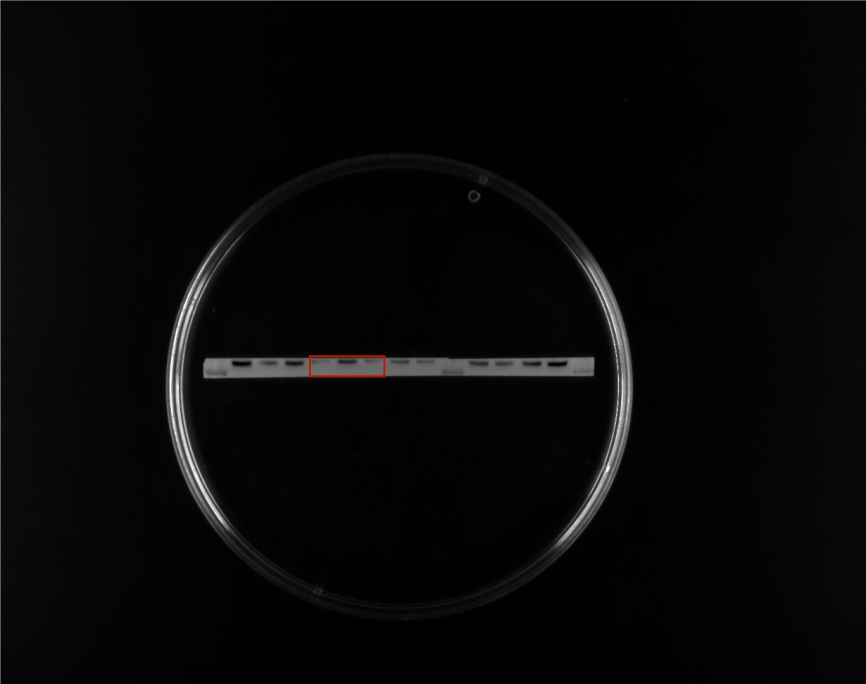
**

**LC3I/LC3II**

**
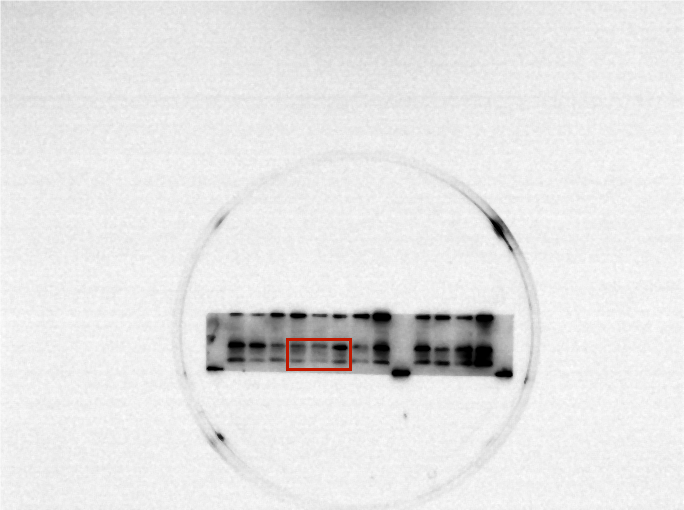
**

**β-Actin for LC3I/LC3II and p62**

**
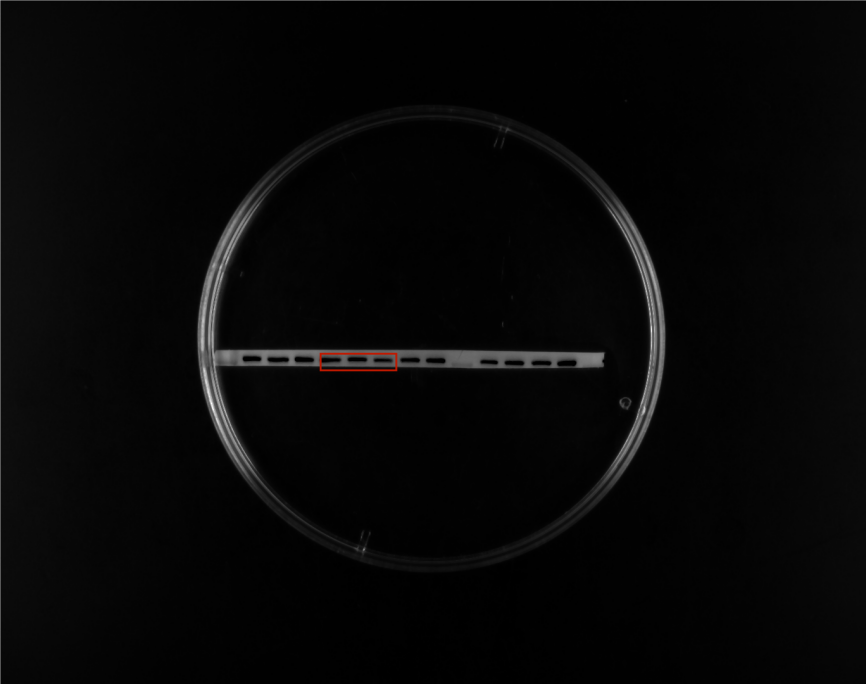
**

**LAPTM5**

**
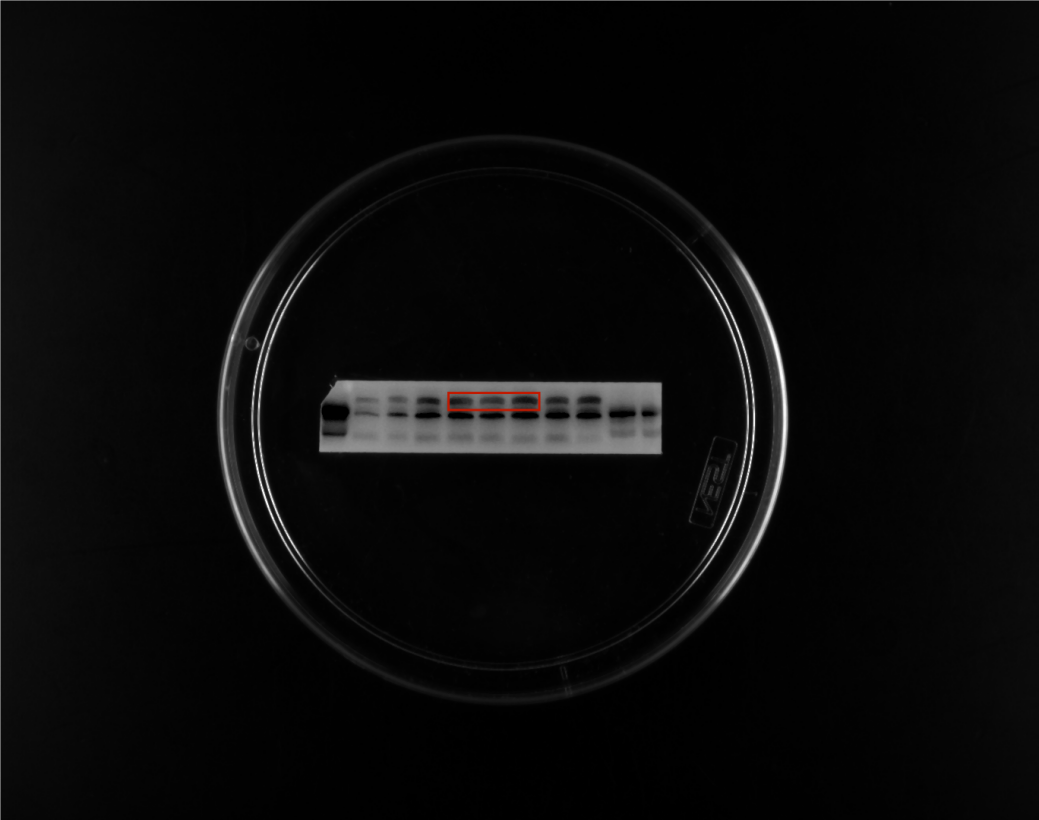
**

**β-Actin for LAPTM5**

**
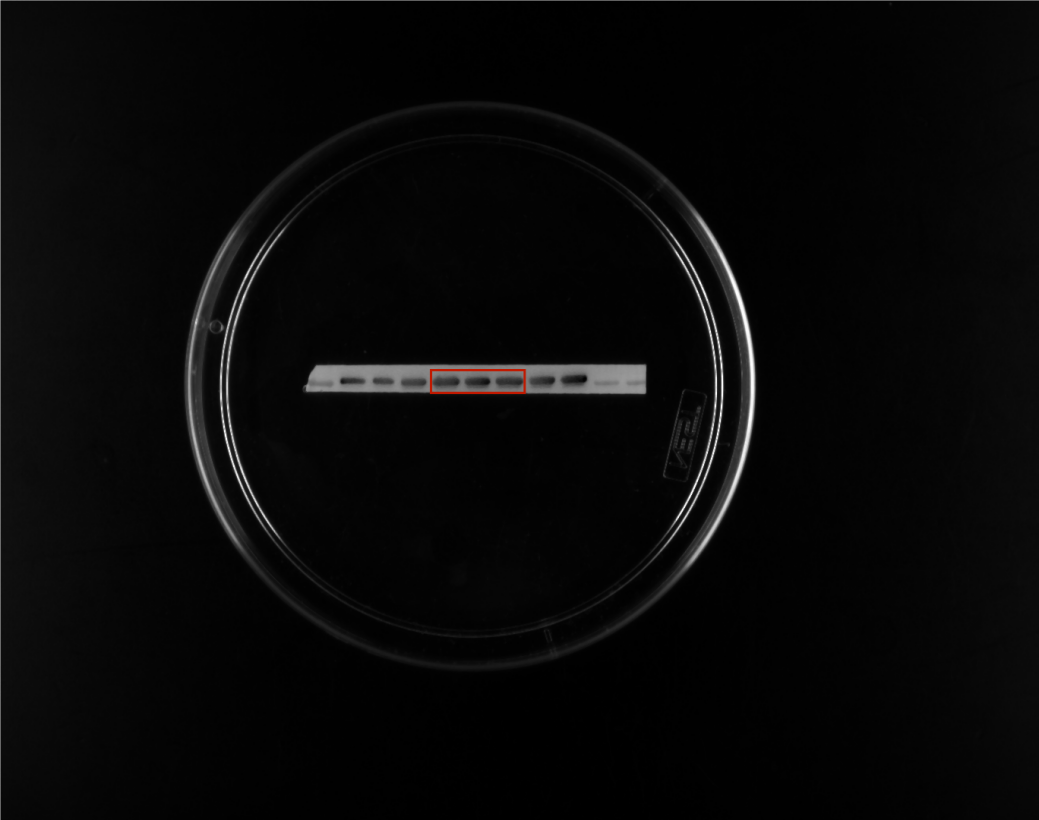
**

**Anti-Flag**

**
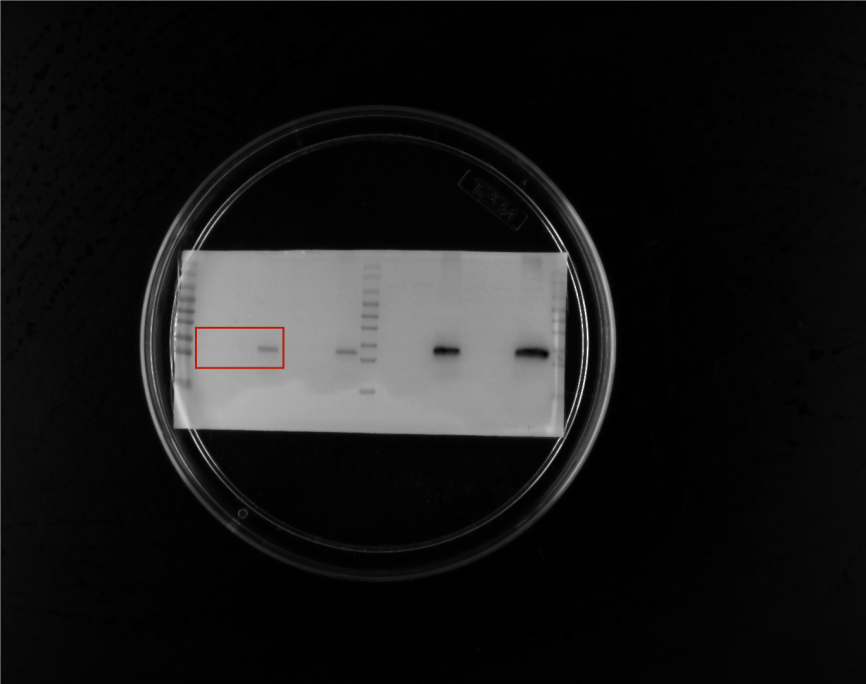
**

**β-Actin for Anti-Flag**

**
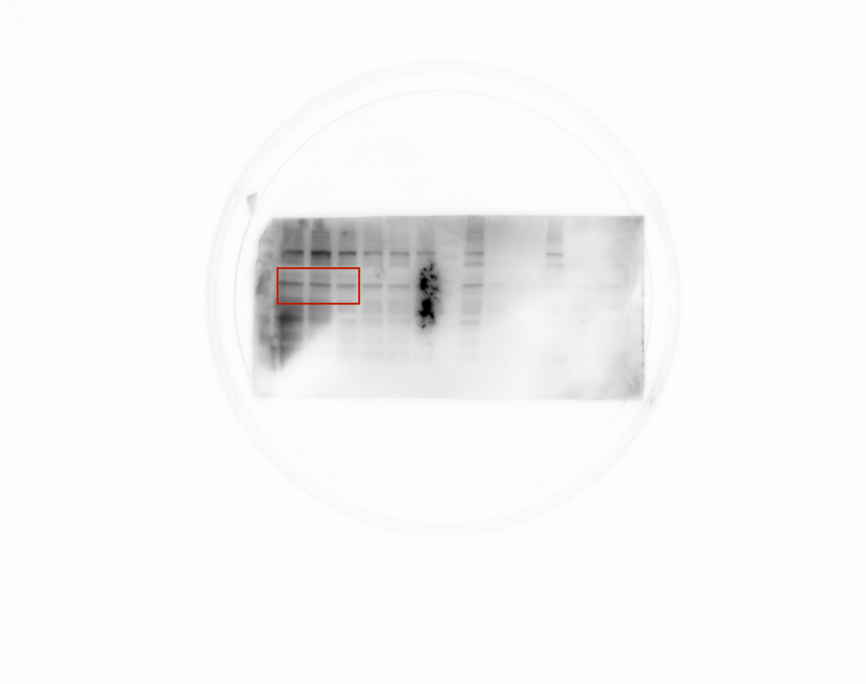
**

**Fig 4K**

**p62**

**
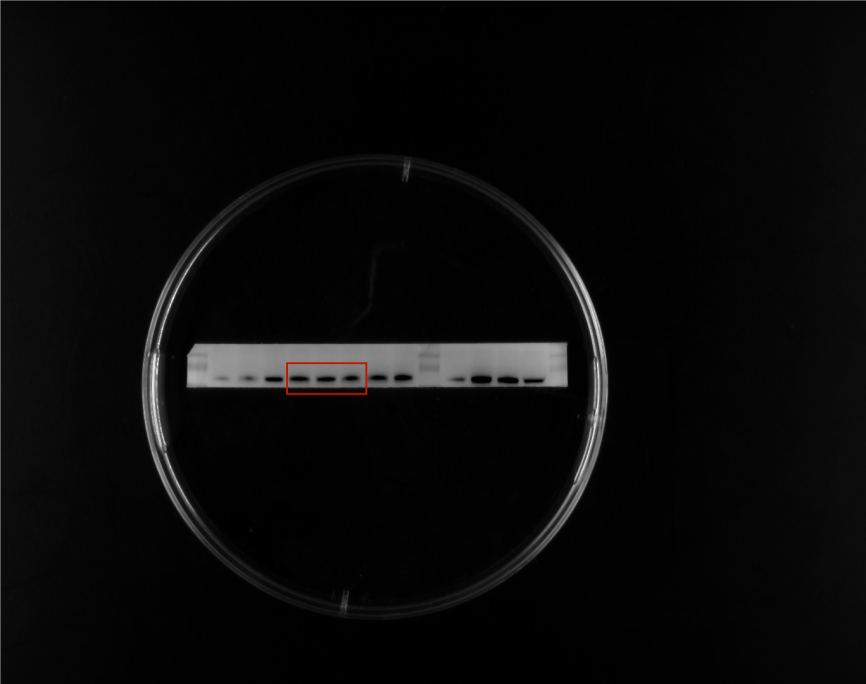
**

**LC3I/LC3II**

**
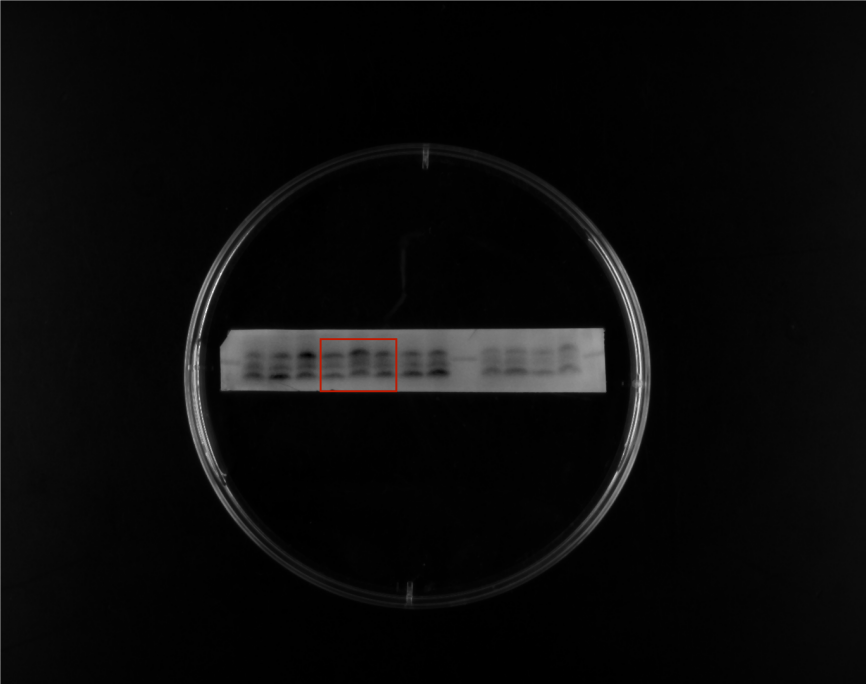
**

**LAPTM5**

**
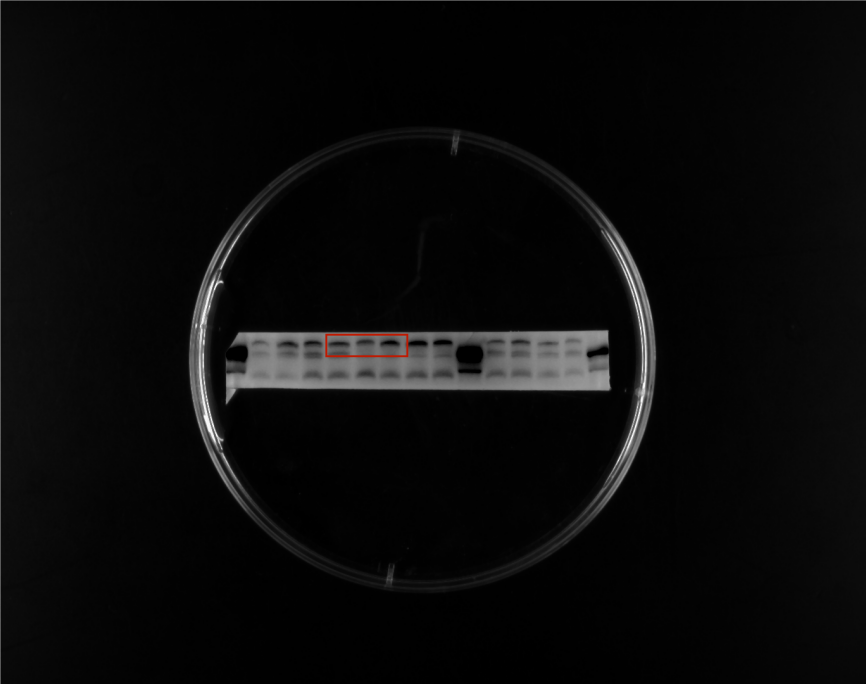
**

**β-Actin**

**
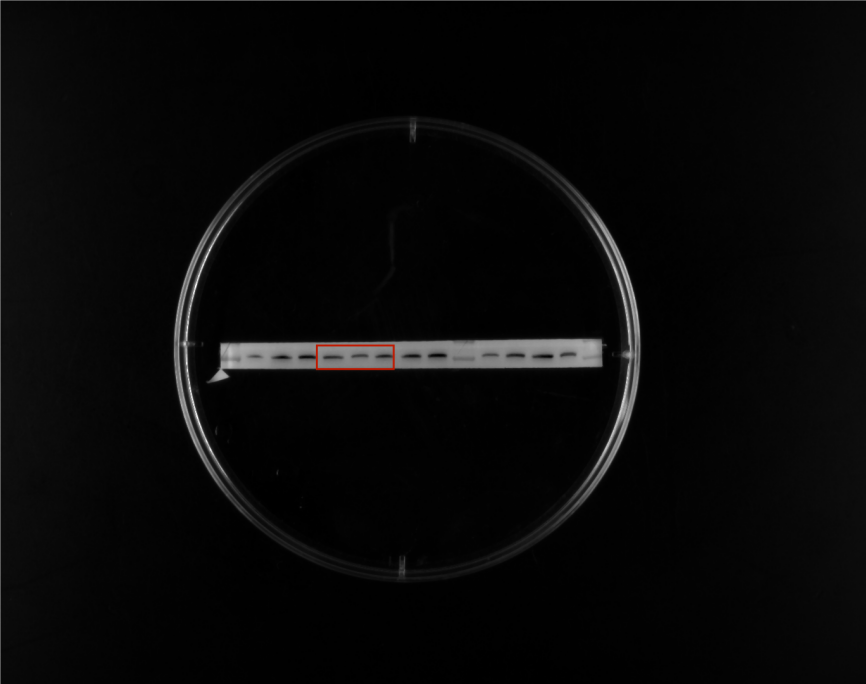
**

**Anti-Flag**

**
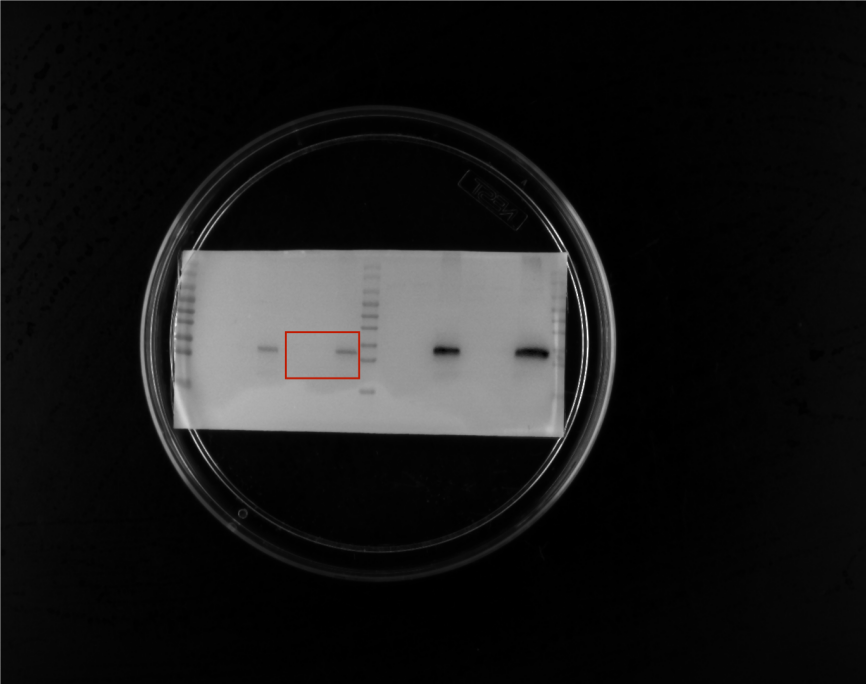
**

**β-Actin for Anti-Flag**

**
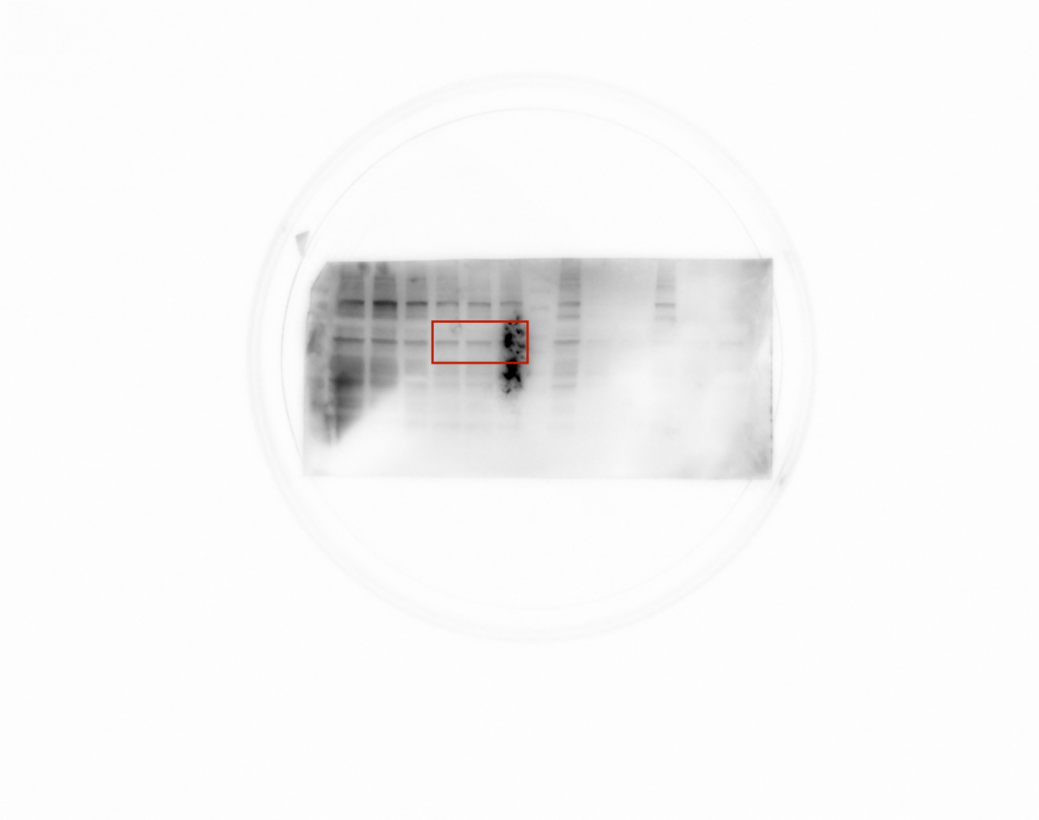
**

**Fig 5G**

**LAMP1**

**
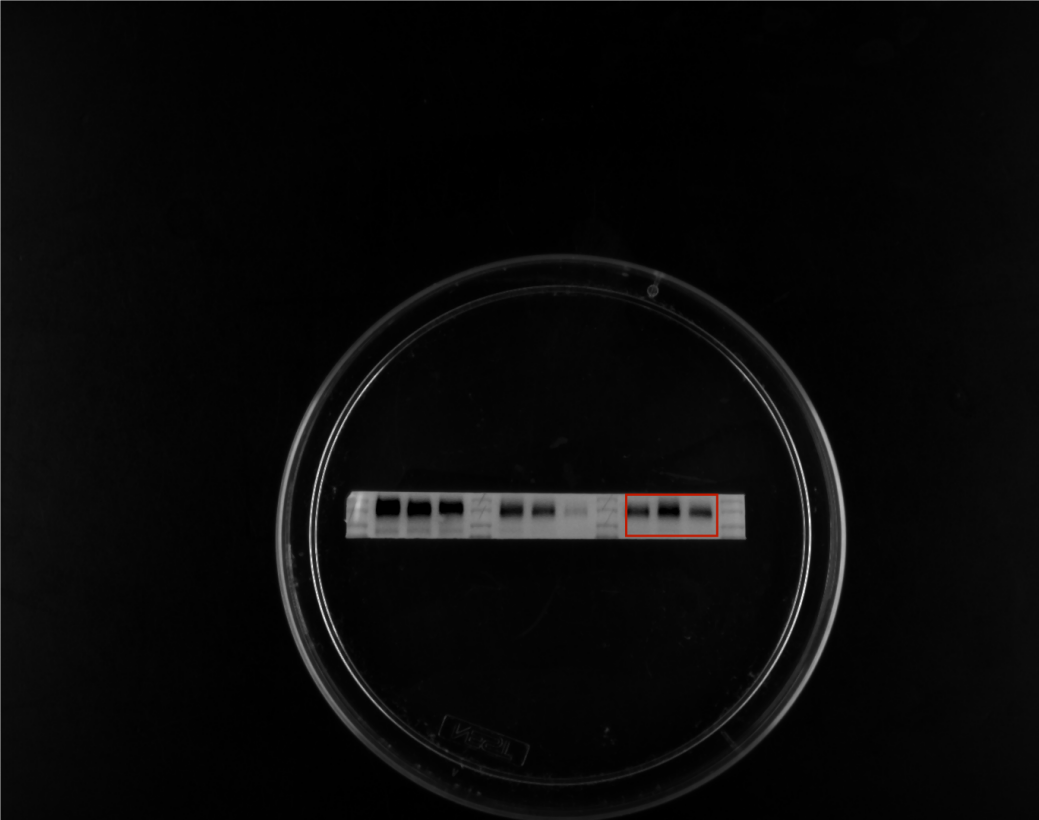
**

**LAPTM5**

**
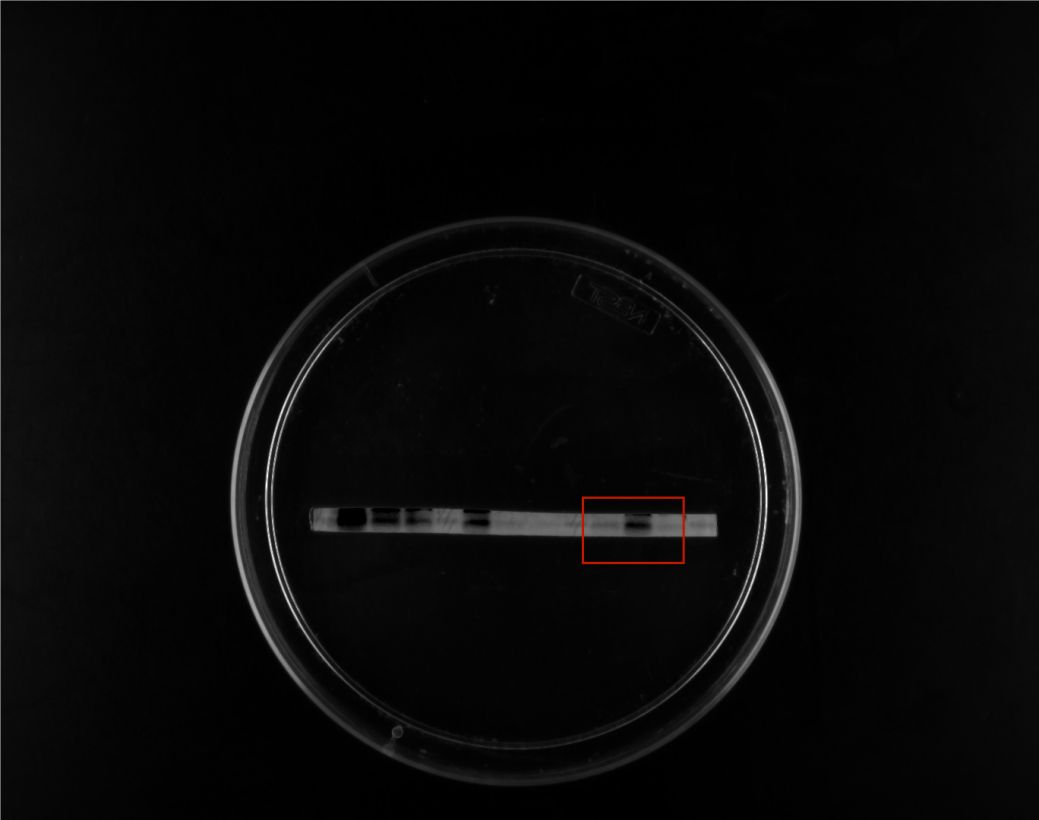
**

**β-Actin for LAMP1 and LAPTM5**

**
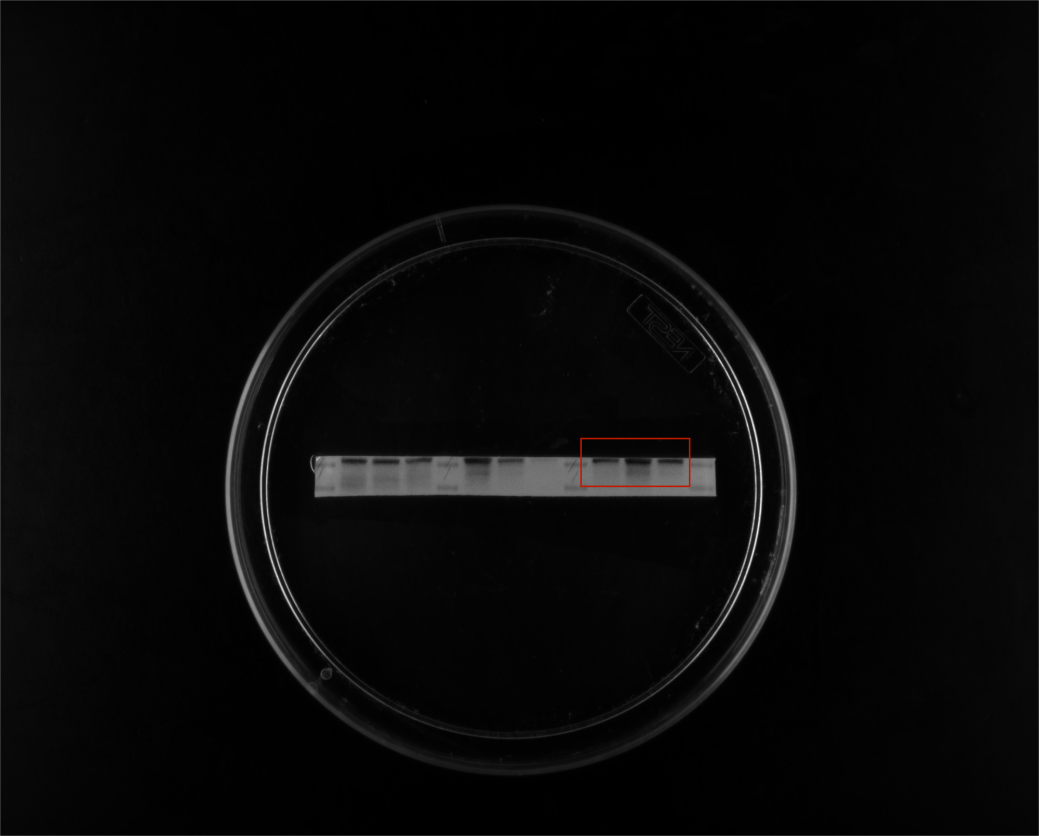
**

**LAMP2**

**
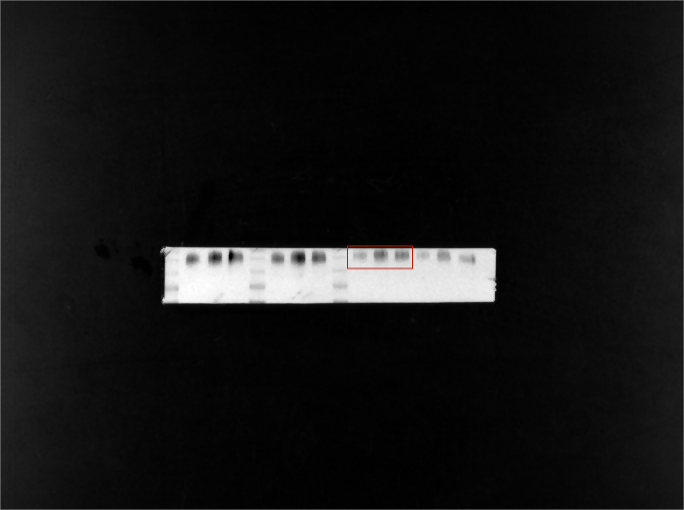
**

**β-Actin for LAMP2**

**
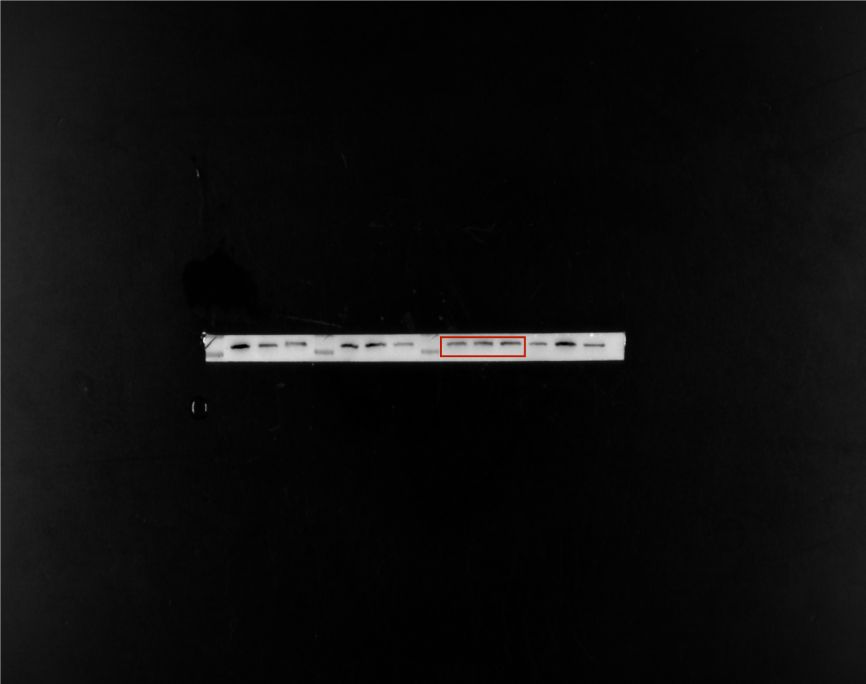
**

**Fig 5H**

**LAMP1**

**
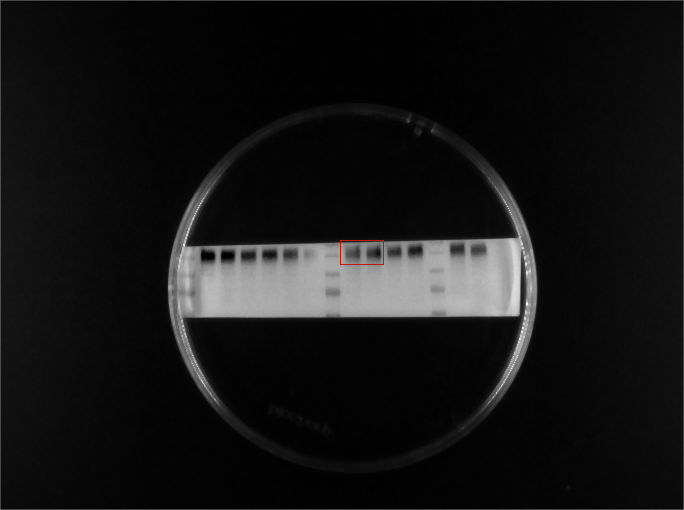
**

**β-Actin for LAMP1**


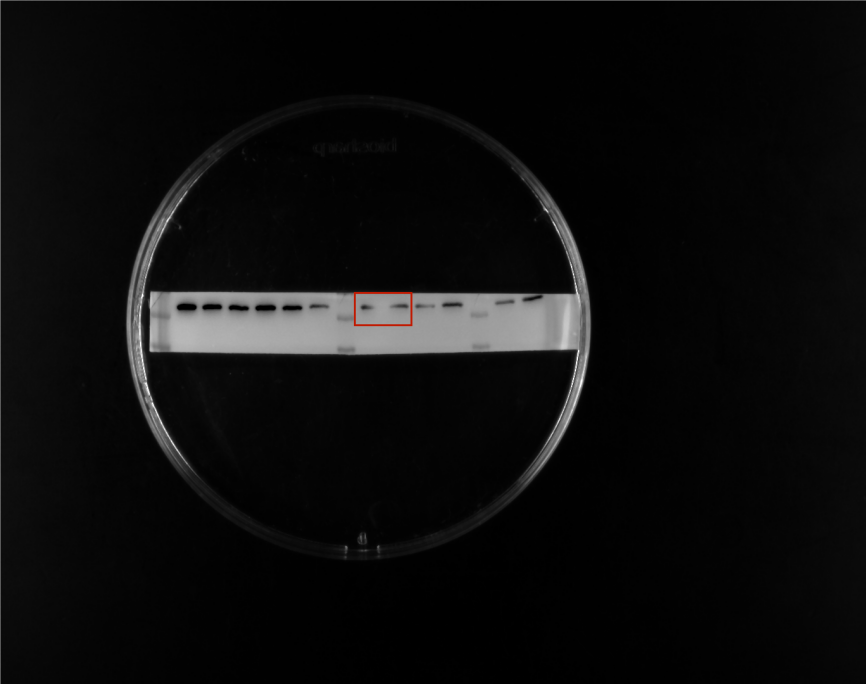


**LAMP2**

**
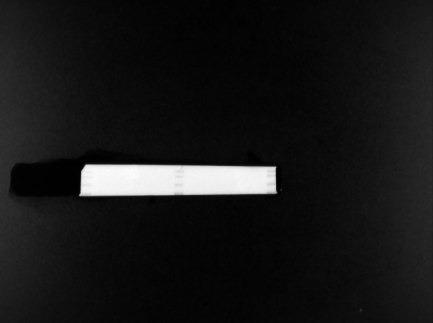

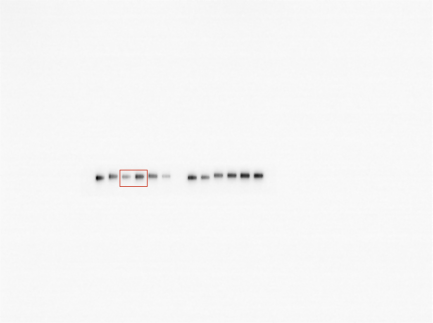
**

**β-Actin for LAMP2**

**
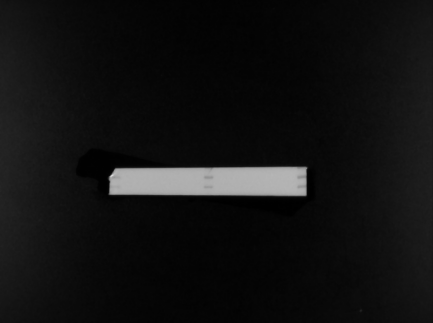

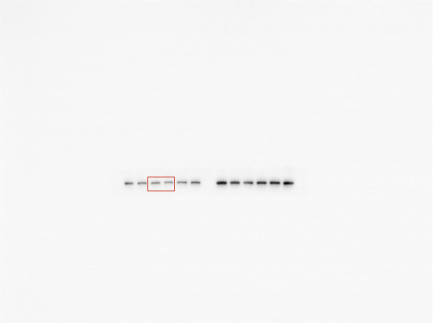
**

**LAPTM5**

**
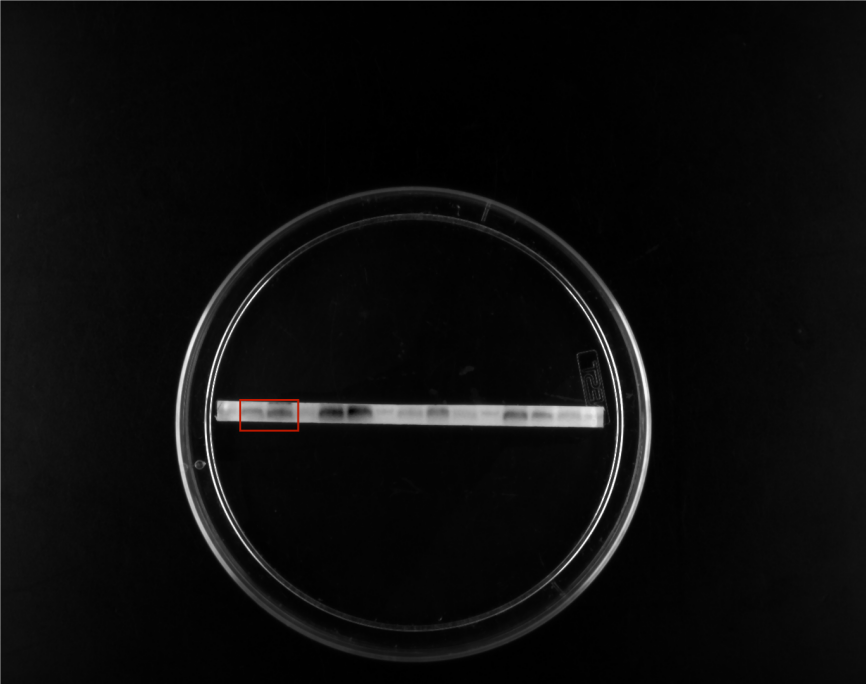
**

**β-Actin for LAPTM5**

**
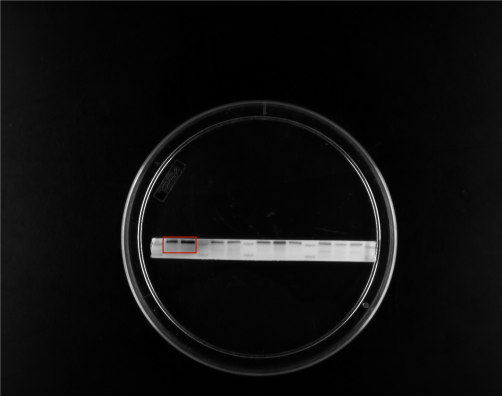
**

**Fig 5I**

**LAMP1**

**
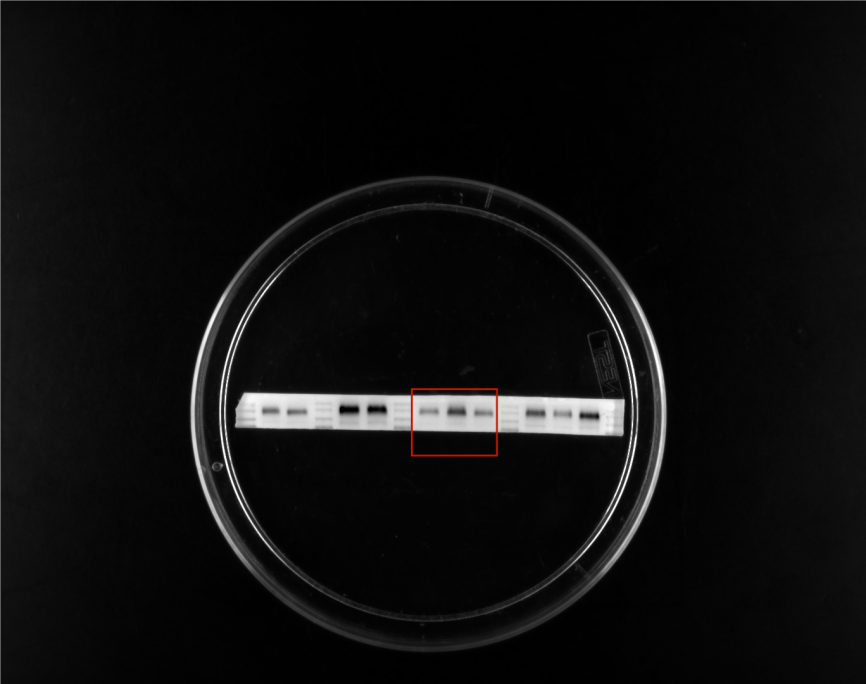
**

**β-Actin for LAMP1**

**
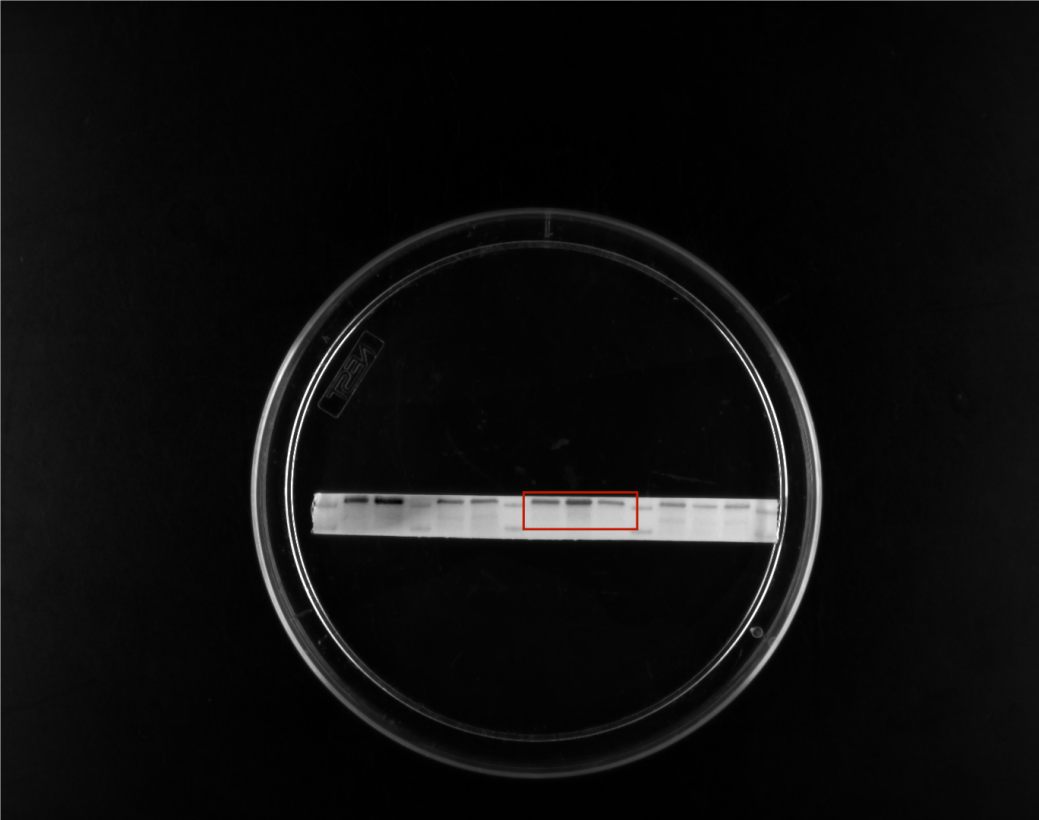
**

**LAMP2**

**
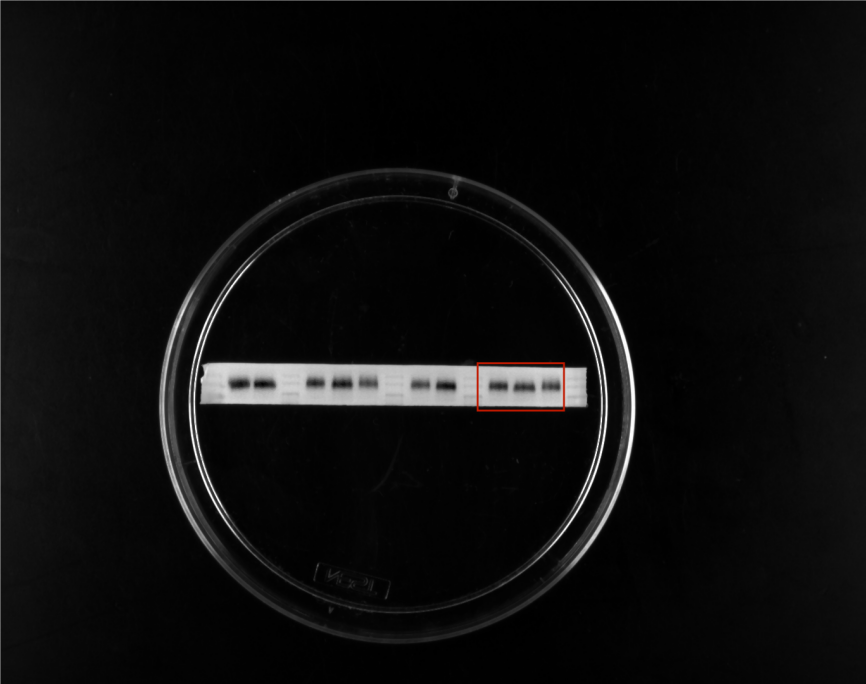
**

**LAPTM5**

**
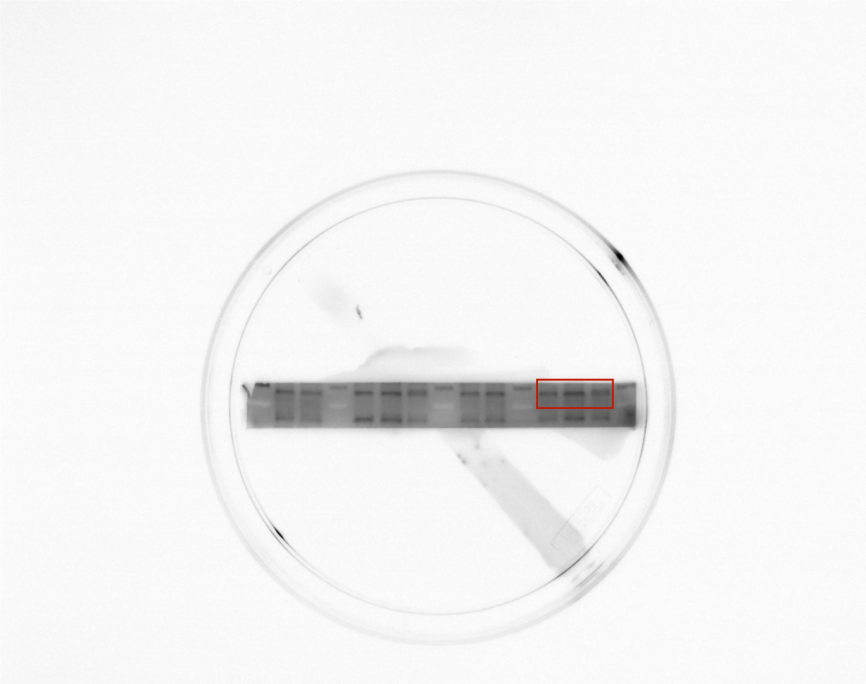
**

**β-Actin for LAMP2 and LAPTM5**

**
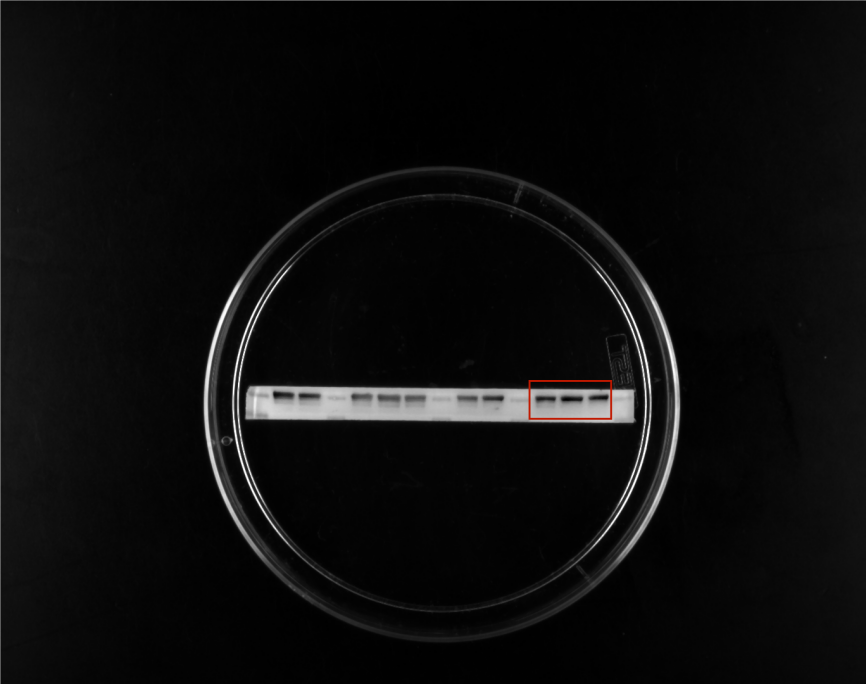
**

**Fig 5J**

**LAMP1**

**
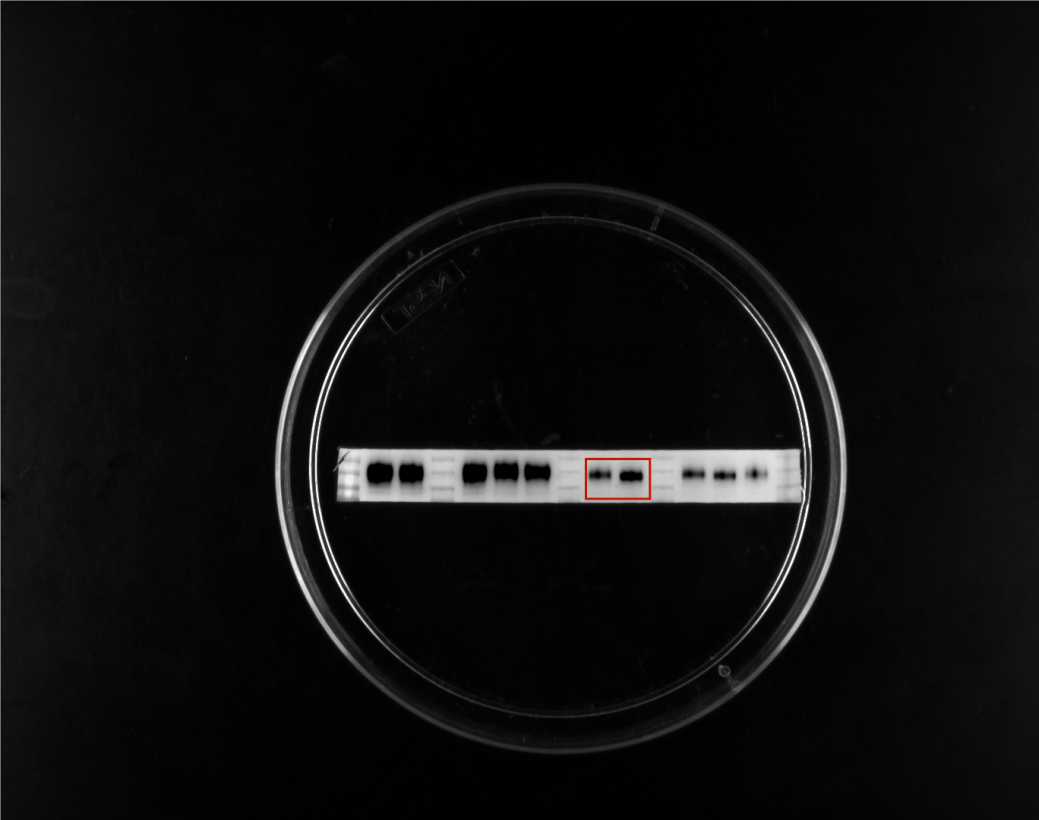
**

**β-Actin for LAMP1**

**
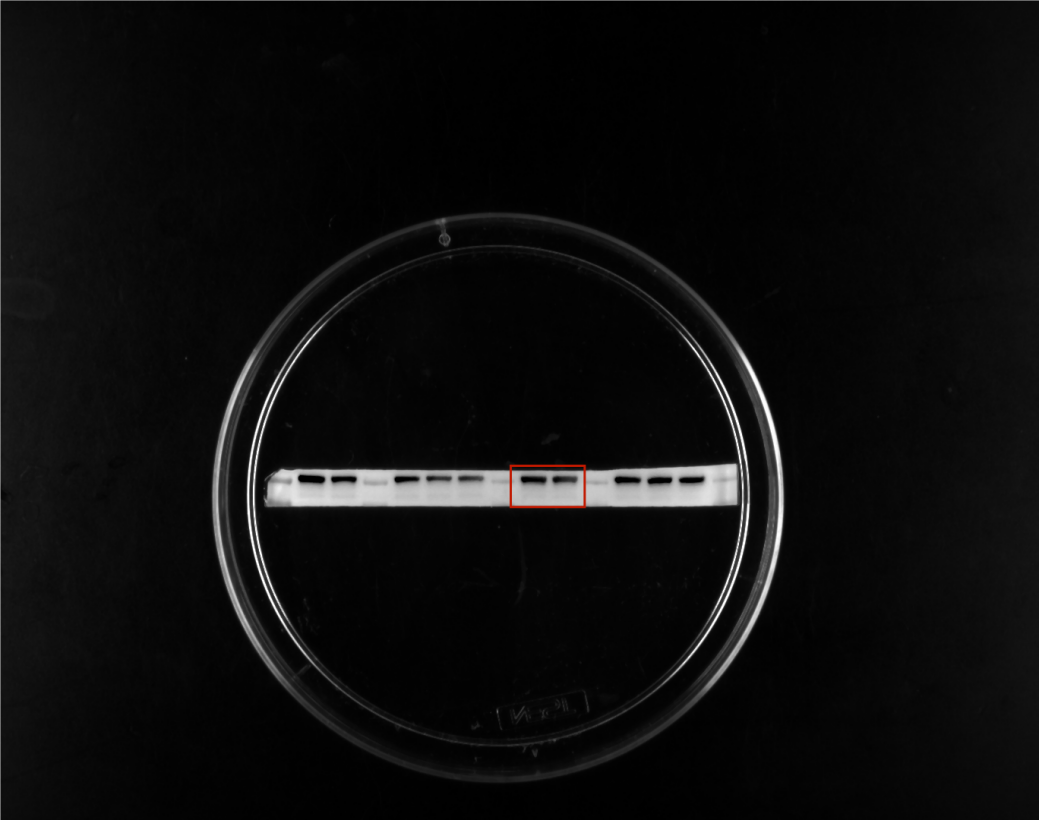
**

**LAMP2**

**
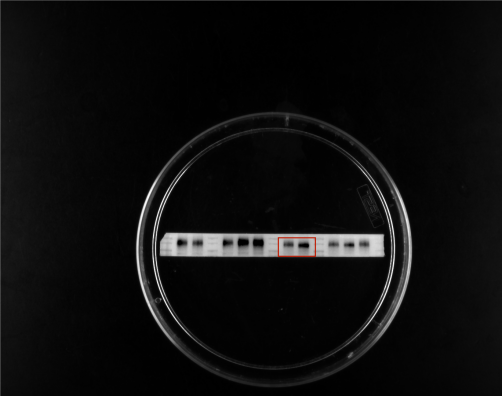
**

**LAPTM5**

**
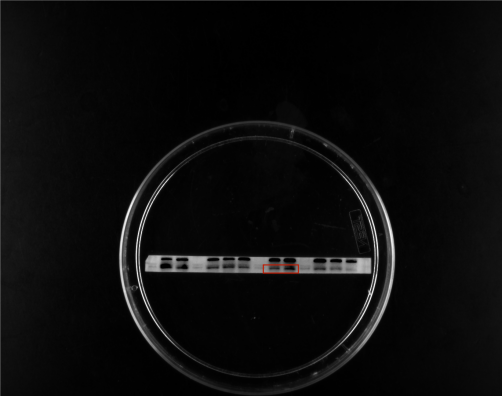
**

**β-Actin for LAMP2 and LAPTM5**

**
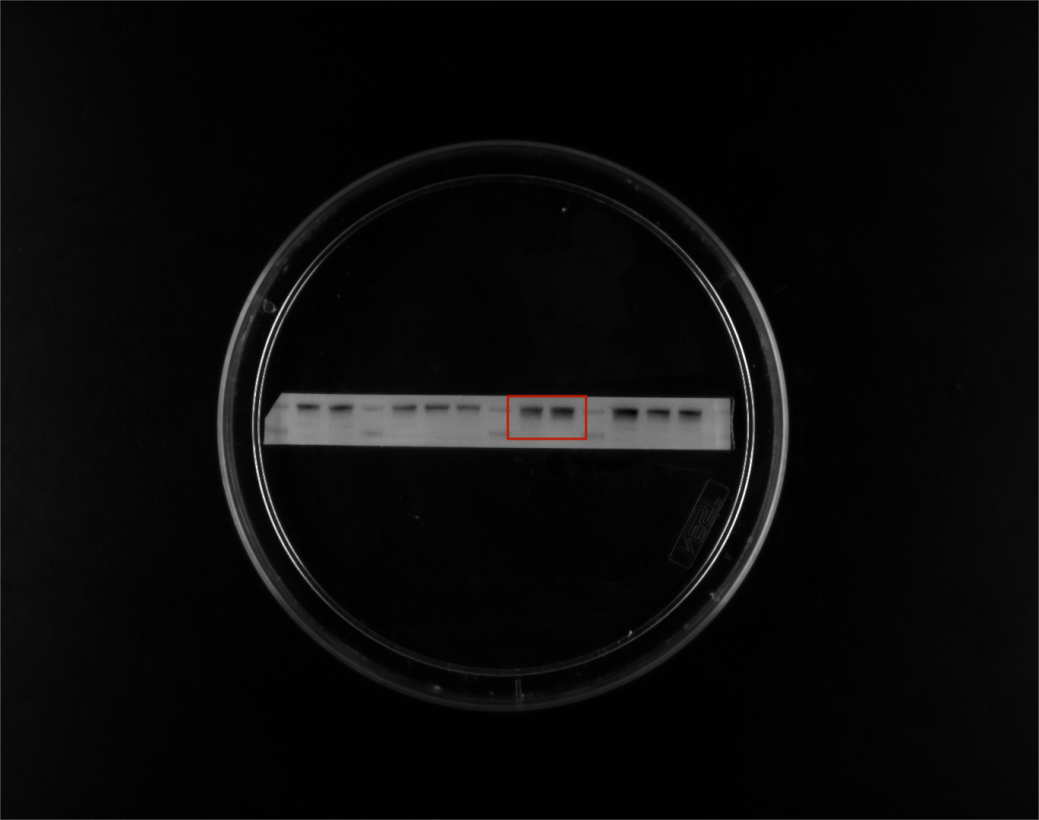
**

**Fig.S3D**

**PARP**

**
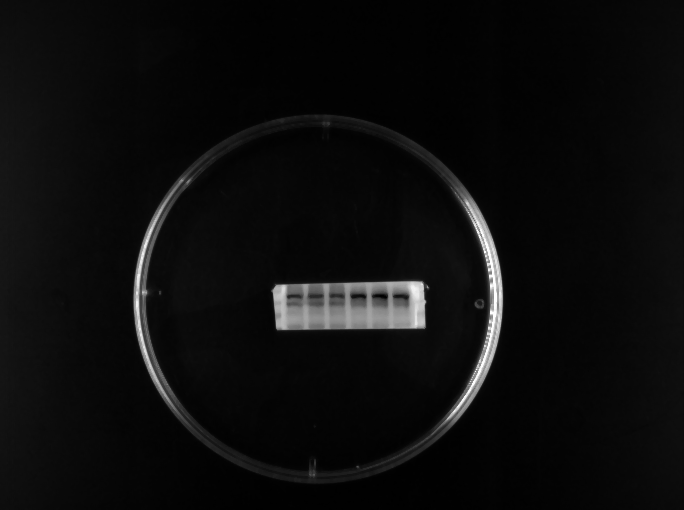
**

**Pro-CASP3/Cld-CASP3**

**
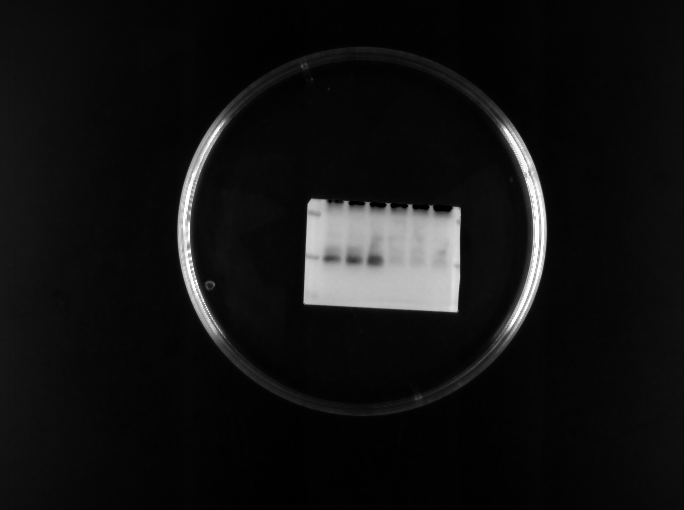
**

**β-Actin**

**
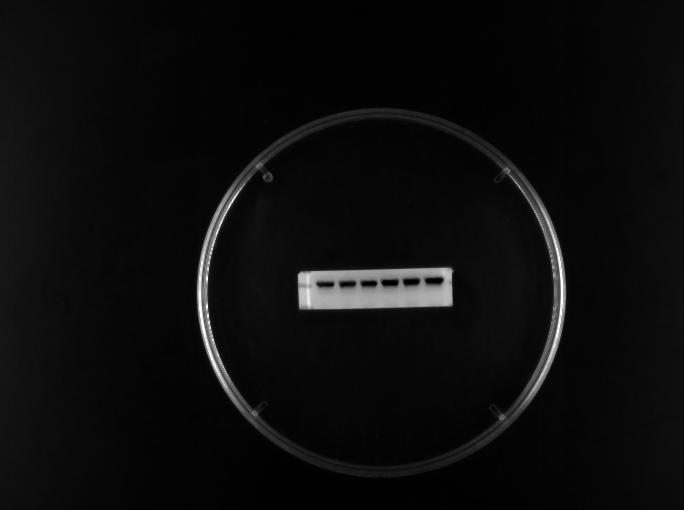
**
